# Guanidine Derivatives Containing the Chalcone Skeleton Are Potent Antiproliferative Compounds against Human Leukemia Cells

**DOI:** 10.3390/ijms232415518

**Published:** 2022-12-08

**Authors:** Francisco Estévez-Sarmiento, Ester Saavedra, Ignacio Brouard, Jesús Peyrac, Judith Hernández-Garcés, Celina García, José Quintana, Francisco Estévez

**Affiliations:** 1 Departamento de Bioquímica y Biología Molecular, Fisiología, Genética e Inmunología, Instituto Universitario de Investigaciones Biomédicas y Sanitarias (IUIBS), Grupo de Química Orgánica y Bioquímica, Universidad de Las Palmas de Gran Canaria, Unidad Asociada al Consejo Superior de Investigaciones Científicas (CSIC), 35016 Las Palmas de Gran Canaria, Spain; 2 Instituto Canario de Investigación del Cáncer (ICIC), 35016 Las Palmas de Gran Canaria, Spain; 3 Instituto de Productos Naturales y Agrobiología, Consejo Superior de Investigaciones Científicas, 38206 La Laguna, Spain; 4 Instituto Universitario de Bio-Orgánica AG, Departamento de Química Orgánica, Universidad de La Laguna (Tenerife), 38200 San Cristóbal de La Laguna, Spain

**Keywords:** apoptosis, caspases, cell cycle, cytotoxicity, hybrid chalcones, guanidines

## Abstract

In this study, we investigated the effects of eleven synthetic guanidines containing the 1,3-diphenylpropenone core on the viabilities of six human cancer cells. The most cytotoxic compound against human cancer cells of this series contains a *N*-tosyl group and a *N*-methylpiperazine moiety **6f**. It was cytotoxic against leukemia cells (U-937, HL-60, MOLT-3, and NALM-6) with significant effects against Bcl-2-overexpressing U-937/Bcl-2 cells as well as the human melanoma SK-MEL-1 cell line. It exhibited low cytotoxicity against quiescent or proliferating human peripheral blood mononuclear cells. The IC_50_ value for the leukemia U-937 cells was 1.6 ± 0.6 µM, a similar value to that in the antineoplastic agent etoposide. The guanidine containing a *N*-phenyl substituent **6i** was also as cytotoxic as the guanidine containing the *N*-tosyl substituent and the *N*-methylpiperazine group **6f** against human U-937 leukemia cells and both synthetic guanidines were potent apoptotic inducers. Cell death was mediated by the activation of the initiator caspase-9 and the executioner caspase-3, and associated with the release of cytochrome *c*. These synthetic guanidines are potent cytotoxic compounds against several human leukemia cells and even the human melanoma cell line SK-MEL-1 and might be useful in the development of new strategies in the fight against cancer.

## 1. Introduction

The development of new hybrid cancer drugs by the combination of two or more biologically relevant “parent” molecules can improve bioactivity, specificity, and help overcome drug resistance. Incorporating a natural product’s privileged structure such as chalcone as one of the parent molecules is an excellent starting point to develop new drugs [1]. Chalcones, consisting of a diaryl propenone system, are the major precursors for the biosynthesis of flavonoids and can interact with several cancer drug targets, exhibiting promising in vitro and in vivo activities against both drug-susceptible and drug-resistant cancers [2]. The chalcone core is considered an appropriate chemical entity to implement rational fragment-based drug discovery strategies in the search of novel anticancer agents [3]. These chemical entities are highly suitable for further optimization based on systematic functionalization of the starting fragment core, taking into account their pharmacological and physicochemical properties. Recently, numerous chalcone hybrids have been prepared and evaluated as potential anticancer agents. Some of them have shown excellent potency against tumor cells in vitro and in vivo, indicating that these compounds may have therapeutic value [3,4].

Guanidines are versatile organosuperbases and are able to bind to carboxylates, phosphates, and metals. The guanidinium cation participates in special interactions between ligands and receptors or enzymes and substrates and may have important biological properties and chemical and pharmaceutical implications. They are of great interest in medicinal chemistry and constitute a key motif of many clinical drugs including the anti-diabetic drug dimethyldiguanide and the peptic ulcer drug cimetidine. Guanidine derivatives are also widely distributed in nature and have attracted a lot of attention due to their chemical diversity and broad biological activities [5,6].

The aim of this study was (i) to synthesize a series of benzyloxychalcones containing a trisubstituted guanidine functional group and (ii) to explore the effects of different substituents on the cytotoxic effects of guanidine functionality against six human cancer cell lines. These substituents included (i) the presence of a *p*-tosylsulfonamide or phenyl moiety on one of the nitrogen atoms of the guanidine functional group and (ii) the presence of different groups such as the isopropyl, diisopropyl, phenyl, or a heterocycle on another nitrogen atom of the guanidine functional group. In addition, we explored whether the most cytotoxic compounds induce cell death by apoptosis in leukemia cells.

## 2. Results

### 2.1. Synthesis

**Scheme 1:**
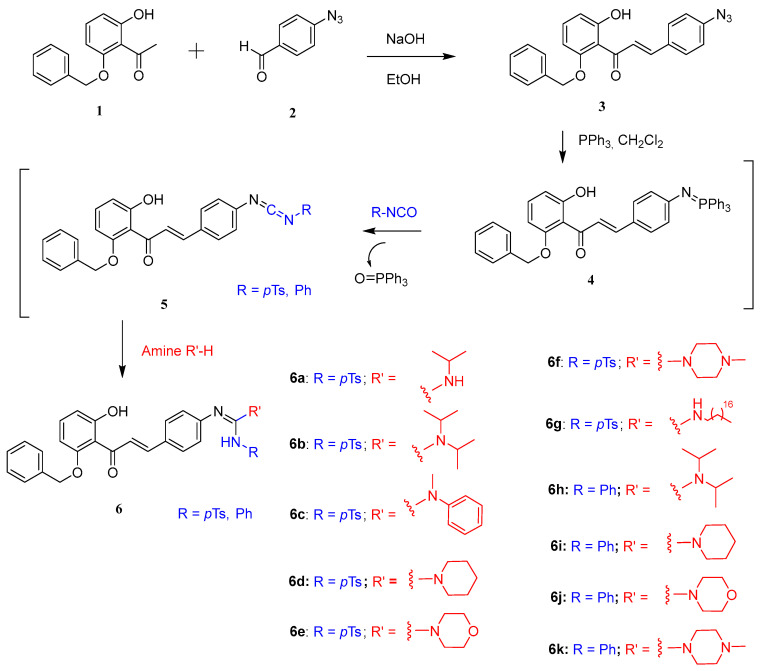
Synthesis of trisubstituted guanidine–chalcone hybrids.

In the present study, we explored the effects of a collection of 11 trisubstituted guanidine-chalcone hybrids on viability of six human cancer cell lines. These compounds were prepared in an efficient one-pot process in which three reactions were carried out in the same solvent through the sequential addition of three reagents as indicated below. This atom-economy procedure incorporates almost all atoms of starting materials and reagents into the final product by the attachment of chemical building blocks. The azidochalcone **3** was prepared in a straightforward manner by a standard aldolic condensation procedure combining the acetophenone **1** with the benzaldehyde **2**. The azidochalcone **3** was treated with triphenylphosphine until the complete formation of iminophosphorane **4**. The isocyanate was then added to the iminophosphorane compound to produce carbodiimide **5**, which was immediately transformed into guanidine **6** by adding different amines (Scheme 1). This synthesis is based on a similar one described previously by Foster et al. [1]. Table 1 shows the chemical structures of the synthesized guanidine–chalcone hybrids **6a**–**6k** and the starting reagents.

### 2.2. Hybrid Compounds Inhibit the Viability of Human Cancer Cells

The potential cytotoxicity of hybrid compounds containing the chalcone skeleton and a guanidine group was evaluated using several human cancer cells (Table 2). These cancer cells included the histiocytic lymphoma U-937, the acute promyelocytic leukemia HL-60, the acute lymphoblastic leukemia MOLT-3, the pre-B NALM-6, the cell line over-expressing the human Bcl-2 protein (U-937/Bcl-2), and the melanoma SK-MEL-1 cell line.

The studies of the effect on cell viability of this series of compounds revealed that in the case of hybrid compounds containing the *p*-tosylsulfonyl group, the cytotoxicity was dependent on the corresponding amine. As shown in Table 2, the isopropylamino derivative **6a** and the phenylmethylamino derivative **6c** were more potent than the corresponding diisopropylamino derivative **6b**. The cytotoxicity increased in the following order: diisopropylamino **6b** < isopropylamino **6a** ~ phenylmethylamino **6c**, except for MOLT-3 cells, in which the three compounds showed similar IC_50_ values and for NALM-6 in which the most cytotoxic compound was the isopropylamino derivative **6a**. There was an important change in cytotoxicity for compounds with a heterocycle structure as a substituent, according to whether the amino group was from a piperidine **6d**, a morpholine **6e,** or a *N*-methylpiperazine **6f**. In these experiments, etoposide and doxorubicin were used as positive controls. In general, the most cytotoxic compound was the hybrid compound **6f**. The greatest differences in cytotoxicity were observed in U-937 cells in which the order of cytotoxicity was: **6f** > **6e** > **6d**. In MOLT-3 cells, these three derivatives were almost equally potent with IC_50_ values between 1.5 µM and 3.3 µM, and in U-937/Bcl-2 cells, the morpholine derivative **6e** showed similar potency to the piperazine derivative **6f**. As shown in Table 2, the *N*-methylpiperazine derivative **6f** was the most cytotoxic compound in all cell lines assayed including the melanoma cell line SK-MEL-1. The IC_50_ values were between 1.5 µM and 4.5 µM, similar to the antineoplastic etoposide, which showed IC_50_ values of 1.8 ± 0.2 µM, 0.6 ± 0.2 µM, and 0.2 ± 0.1 µM for U-937, HL-60 and MOLT-3, respectively. However, the substitution of the cyclic amine **6f** for an amine containing a long (C18) aliphatic chain yielded a compound (hybrid compound **6g**) which was not cytotoxic against the cancer cell lines assayed, probably due to its low solubility.

In the case of *p*-tosylsulfonyl derivatives, the most potent compound contains a *N*-methylpiperazine **6f** and the order of potency was: *N*-methylpiperazine **6f** > morpholine **6e** > piperidine **6d**, mainly in U-937 and SK-MEL-1. However, compounds **6e** and **6f** showed similar IC_50_ values in HL-60, NALM-6, and U-937/Bcl-2, and compounds **6d**, **6e,** and **6f** were equally potent against MOLT-3.

Among the *N*-phenyl derivatives, compound **6i** containing a piperidine ring was, in general, the most cytotoxic compound with IC_50_ values that were either less than (U-937, HL-60, MOLT-3, and U-937/Bcl-2) or close to (NALM-6, SK-MEL-1) 10 µM. The substitution of the piperidine **6i** ring by a *N*-methylpiperazine **6k** ring determined a decrease in cytotoxicity. In contrast, the substitution of the piperidine **6i** by a morpholine **6j** increased the cytotoxicity against MOLT-3, NALM-6, and SK-MEL-1.

In general, over expression of Bcl-2 did not confer protection against hybrid compounds-induced cytotoxicity since the IC_50_ values were similar or even lower than that for the parental U-937 cells. In U-937/Bcl-2 cells, the *p*-tosyl derivatives containing the morpholine ring **6e**, the phenyl group **6c,** and *N*-methylpiperazine **6f** were equally potent showing IC_50_ values between 2.2 ± 0.8 µM and 4.2 ± 1.2 µM. In these cells, the *N*-phenyl guanidine derivative containing the piperidine ring **6i** was equally cytotoxic as it was against the parental U-937 cell line. Most compounds assayed in MOLT-3 cells were equally potent, showing IC_50_ values below 10 µM. The exceptions were the *p*-tosyl derivative containing an octadecyl substituent **6g** and the *N*-phenylguanidine containing the *N*-methylpiperazine ring **6k**. A similar trend was observed in NALM-6 cells since most compounds showed similar IC_50_ values. The exception was the *p*-tosyl derivative **6b**, which showed low cytotoxicity against NALM-6. The substitution of the *p*-tosyl-sulfonyl group **6b** by a phenyl group **6h** increased the cytotoxicity five-fold (IC_50_ = 27.0 ± 0.8 µM vs. IC_50_ = 5.4 ± 0.2 µM). The piperidine derivative (compound **6i**) was more potent than the *p*-tosylsulfonyl derivative (compound **6d**) in U-937, U-937/Bcl-2, HL-60, and SK-MEL-1 cells.

The structure–activity relationship (SAR) reveals that the introduction of the guanidine group enhances the cytotoxicity of the chalcone skeleton in comparison with the azidochalcone **3**, and the results of the cytotoxicity assays of *p*-tosylsulfonyl derivatives revealed that the most potent compound contains a *N*-methylpiperazine ring and the order of potency was *N*-methylpiperazine > morpholine > piperidine.

The cytotoxicity assays of the *N*-phenyl derivatives series revealed that: (i) the hybrid compound **6i** containing a piperidine ring was, in general, the most cytotoxic compound showing IC_50_ values below 10 µM, except in NALM-6 (IC_50_ = 10.8 ± 0.5 µM) and SK-MEL-1 (IC_50_ = 13.8 ± 1.2 µM) cells; (ii) the substitution of the piperidine **6i** by a morpholine ring **6j** decreased the cytotoxicity against human U-937, HL-60, and U-937/Bcl-2 cells; and (iii) the substitution of the piperidine ring **6i** by a *N*-methylpiperazine ring **6k** decreased the cytotoxicity against all cell lines assayed.

**Figure 1:**
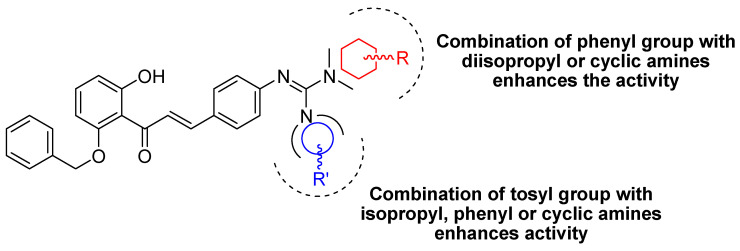
Structure–activity relationship of guanidines containing the 1,3-diphenylpropenone core against U-937 and HL-60 leukemic cells.

The SAR against U-937 and HL-60 leukemia cells of this series of hybrid compounds containing the chalcone skeleton and a guanidine group is displayed graphically in Figure 1.

**Figure 2:**
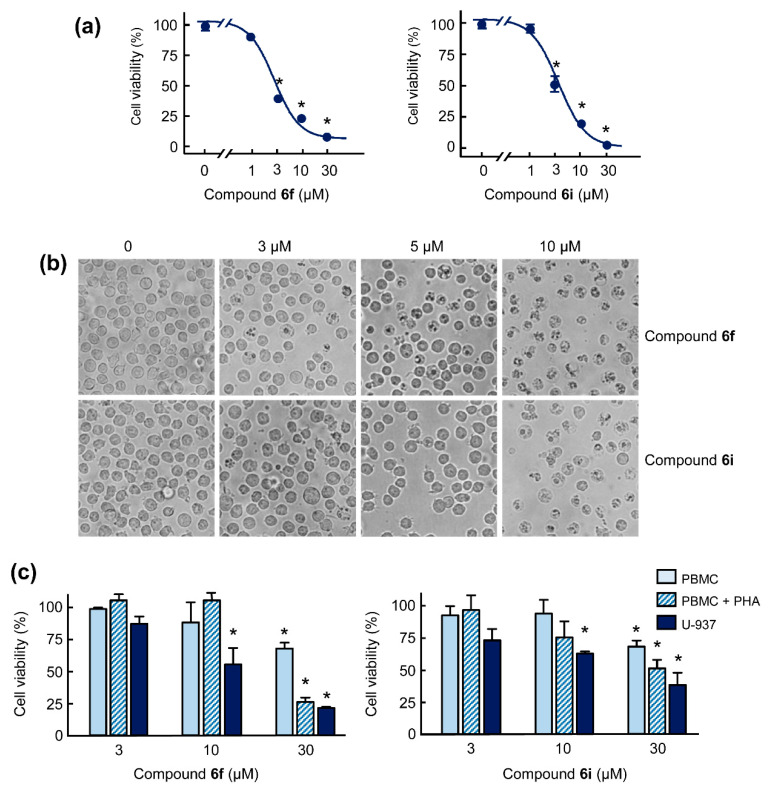
(**a**) Dose-response study of hybrid chalcone–guanidines on human U-937 leukemia cell viability. Cells were cultured in the presence of increasing concentrations of selected guanidines for 72 h, and thereafter cell viability was determined by the MTT assay. (**b**) Photomicrographs of morphological changes visualized with an inverted phase-contrast microscope after treatment of U-937 cells with the specified guanidine for 24 h. (**c**) Differential effects of guanidines on cell viability of normal peripheral blood mononuclear cells (PBMC) versus U-937 cells. Human leukemia and quiescent and phytohemagglutinin-activated PBMC (PBMC (+PHA)) cells from healthy human origin were cultured in the presence of the specified concentrations of guanidines for 24 h. Values represent means ± SE of three independent experiments each performed in triplicate. * *p* < 0.05, significantly different from the corresponding control.

The results suggest that the main determinant of cytotoxicity in hybrid compounds containing the *p*-toluensulfonyl group is the presence of a *N*-methylpiperazine ring and the most cytotoxic compound was the hybrid compound **6f** in the six cell lines assayed. As shown in Table 2, compound **6f** showed similar IC_50_ values for U-937 and HL-60 to the values obtained with etoposide, which was included as a positive control. The human leukemia MOLT-3 cells and the cell line overexpressing Bcl-2 (U-937/Bcl-2) were also sensitive to these hybrid compounds. Compound **6i** was also as cytotoxic as compound **6f** in both U-937 and HL-60 cells. Representative concentration-dependent inhibition of cell viability curves of compounds **6f** and **6i** against U-937 are shown in Figure 2a. As visualized by phase-contrast microscopy, these compounds induced significant morphological changes and an important reduction in the number of cells (Figure 2b). To determine the potential selectivity of hybrid compounds **6f** and **6i** against human leukemia cells, we compared the effect against human mononuclear cells (PBMC) obtained from healthy donors. Proliferating and quiescent lymphocytes showed lower cytotoxicity even at 10 µM hybrid compounds for 24 h. Fresh and proliferating PBMC were more resistant to the cytotoxic effects of these compounds (Figure 2c).

### 2.3. Hybrid Compounds Induce Changes in Cell Cycle in Human U-937 Leukaemia Cells

**Figure 3:**
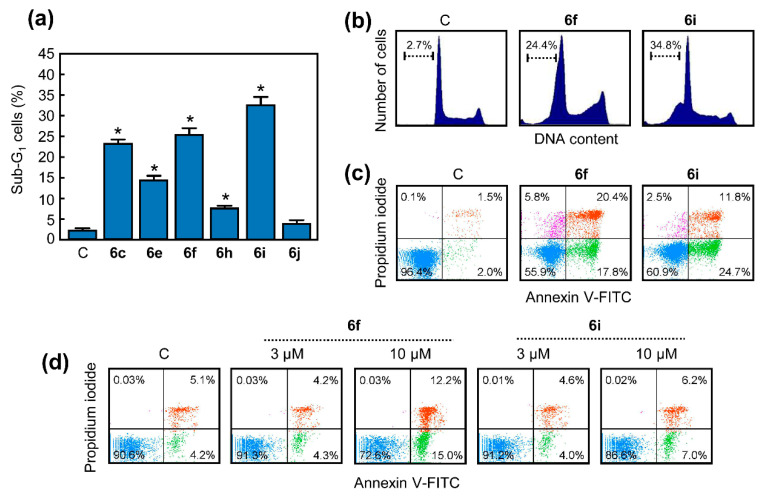
Increase in percentage of apoptotic cells (sub-G_1_ fraction) due to hybrid compounds and externalization of phosphatidylserine by compounds **6f** and **6i** in U-937 cells. (**a**) Cells were incubated with 10 µM of specified chalcone–guanidines for 24 h and apoptotic cells were quantified as percentage of cells in the sub-G_1_ region using flow cytometry after propidium iodide staining. * indicates *p* < 0.05 for comparison with untreated control, C. (**b**) Representative histograms of flow cytometry using propidium iodide staining of cells incubated in the presence of 10 µM of the indicated compound for 24 h. The percentage of sub-G_1_ cells is shown. (**c**) Cells were incubated as indicated in (**b**) and quantified by flow cytometry after double staining with annexin V-FITC and propidium iodide. Cells in the lower right quadrant show positive annexin V-FITC, which indicates phosphatidylserine translocation on the outside of the plasma membrane, and negative propidium iodide staining, which demonstrates intact cell membranes, both features of early apoptosis. Cells appearing in the top right quadrant are double positive for annexin V-FITC and propidium iodide and are undergoing late apoptosis. Data are representative of three independent experiments. (**d**) PBMC were incubated with increasing concentrations of compounds **6f** and **6i** for 24 h and quantified by flow cytometry after double staining with annexin V-FITC and propidium iodide.

Although the compounds **6f** and **6i** were the most cytotoxic against human cancer cells, we were interested in knowing whether other compounds of this series were able to affect the cell cycle. To explore this, U-937 cells were incubated with 10 µM of selected compounds **6c**, **6e**, **6f**, **6h**, **6i,** and **6j** for 24 h, and analyzed by flow cytometry after propidium iodide staining. As shown in Table 3, guanidine **6e** induced an arrest at the G_2_-M phase of the cell cycle. The percentage of control cells in G_2_-M phase was approximately 14%, which increased to 27% and this was accompanied by a significant decrease in cells in the G_1_ phase. This suggests that the effects on viability may be caused at least in part by an arrest of the cell cycle. The percentage of sub-G_1_ (i.e., apoptotic cells) increased until 23.5% (~10-fold), 11.9% (~5-fold), 25.1% (~10-fold), and 31.6% (~13-fold) by compounds **6c**, **6e**, **6f**, and **6i**, respectively (Figure 3a). Representative histograms of flow cytometry after propidium iodide labelling are shown in Figure 3b. To confirm that the decrease in viability triggered by **6f** and **6i** was caused by apoptosis induction, treated cells were double stained with annexin V-FITC and propidium iodide and subjected to flow cytometry. The number of annexin V-FITC positive cells increased 11-fold (3.5% vs. 38%) and 10-fold (3.5% vs. 36%) for compounds **6f** and **6i**, respectively, in U-937 cells confirming that these specific guanidines are potent apoptosis inducers (Figure 3c). Interestingly, dose-response studies revealed that PBMC were more resistant toward compounds **6f** and **6i**. The number of annexin V-FITC positive cells hardly increased with respect to control after treatment with 3 µM of compounds **6f** and **6i**, but increased 3-fold (9.3% vs. 27%) and 1.4-fold (9.3% vs. 13%) with respect to control after treatment with 10 µM compounds **6f** and **6i**, respectively (Figure 3d).

### 2.4. Hybrid Compounds Induce Caspase Activation, PARP Cleavage, and Cytochrome c Release in Human U-937 Leukemia Cells

**Figure 4:**
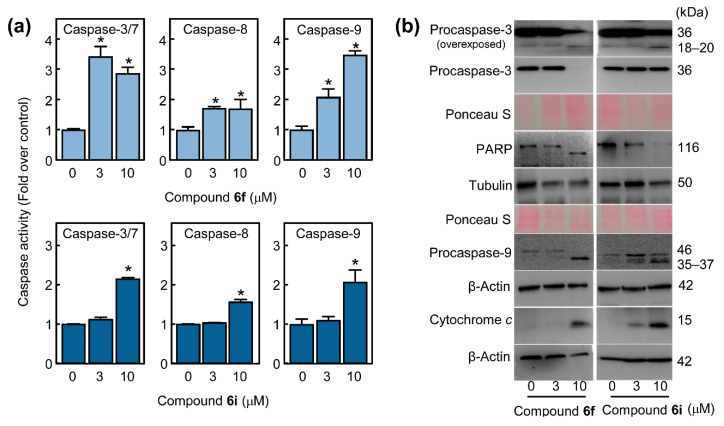
Processing of caspases and PARP (poly(ADP-ribose)polymerase) and mitochondrial cytochrome *c* release in the apoptosis induced by hybrid compounds on leukemia cells. (**a**) Caspase activation induced by guanidines. U-937 cells were treated with the indicated concentrations of compounds and cell lysates were assayed for caspase-3/7, -8, and -9 activities. Results are expressed as n–fold increases in caspase activity compared with the control. Values represent the means ± SE of three independent experiments each performed in triplicate. * indicates *p* < 0.05 for comparison with untreated control. (**b**) U-937 cells were incubated with the indicated concentrations of guanidines for 24 h and the cleavage of caspases and PARP and cytochrome *c* release were analyzed by immunoblotting. β-Actin and tubulin were used as loading controls. Equal loading was also controlled by staining the membranes with Ponceau S before antibody detection.

To determine whether the mechanism of cell death triggered by compounds **6f** and **6i** was associated with caspase activation, the enzyme activities of caspases-3/7, -8, and -9 were determined in lysates using tetrapeptide substrates. As shown in Figure 4a, maximal caspase-3/7 and -9 activities were detected after 24 h of treatment with 10 µM of both guanidines in U-937 cells, while caspase-8 was less activated. Caspase-3/7 and caspase-9 activities increased approximately three-fold and two-fold after treatment with guanidines **6f** and **6i**, respectively.

An indicator of caspase activation is the cleavage of poly(ADP-ribose)polymerase (PARP). This enzyme is involved in DNA repair and PARP cleavage is considered a hallmark of cell death. To explore whether guanidines **6f** and **6i** induced PARP cleavage, lysates of treated cells were analyzed by immunoblotting. As shown in Figure 4b, the full-length PARP protein dramatically decreased after 24 h of treatment with 10 µM of both guanidines indicating the processing of this protein. In accordance with PARP cleavage, the executioner caspase-3 was processed, as detected by the generation of a fragment of 18–20 kDa in the immunoblots. In a similar way as the enzymatic assays, the immunoblot experiments also revealed pro-caspase-9 processing suggesting that PARP cleavage might be attributable to pro-caspase-9 cleavage followed by caspase-3 activation.

Since caspase-9 activation is dependent on the release of cytochrome *c* from the intermembrane space of mitochondria to cytosol, we explored whether the cell death induced by selected guanidines involves cytochrome *c* release. To this end, dose-response experiments were performed and cytosolic fractions were analyzed by Western blot after 24 h of treatment with increasing concentrations of guanidines. As shown (Figure 4b), the level of cytochrome *c* increased in the cytosolic fraction in accordance with the processing and activation of pro-caspase-9. These results indicate that guanidines induce cytochrome *c* release and the processing and activation of caspases.

## 3. Discussion

In the present report, we synthesized a series of chalcone hybrids containing trisubstituted guanidines and explored the potential effects on human cancer cell viability. We found that the *p*-tosyl derivative containing a *N*-methylpiperazine **6f** was the most potent cytotoxic compound against the six cell lines assayed. It seems that the main determinant of this cytotoxicity is the nature of the heterocycle since the *N*-methylpiperazine derivative was more potent than the morpholine or piperidine derivatives. The second most potent inhibitor of cell viability, at least against leukemia cells, was the *N*-phenyl derivative **6i**. Interestingly, dose-response studies revealed that quiescent PBMC and proliferating PBMC were more resistant than the human leukemia U-937 cell line against the cytotoxicity triggered by the most potent guanidines. It is a remarkable finding that PBMC show increased apoptosis resistance toward guanidines **6f** and **6i** since the increases in the percentages of annexin V-FITC positive cells compared with the control were lower than in U-937 cells. The effects on cell viability and the mechanisms involved in cell death induction of these specific compounds have not been investigated to date. Our results demonstrated that at least one of the guanidines, compound **6e** containing a *N*-tosyl group and a morpholine ring, blocks proliferation of leukemia cells by arresting the cells in the G_2_-M phase of the cell cycle. This suggests that compound **6e** might target cyclin-dependent kinase 1 (CDK1) which is a stimulator of cell cycle progression. CDK1 inhibition induces cell cycle arrest in the G_2_-M phase and apoptosis and targeting CDK1 has showed promising results for cancer treatment in different preclinical models [7]. Another possibility might be that compound **6e** can bind tubulin and therefore interfere with the mitotic process as described for chalcone derivatives [8]. Further research is needed to confirm the specific target/s of this compound.

Cell cycle analysis revealed that inhibition of cell viability by the other hybrid compounds (compounds **6c**, **6f**, **6h**, and **6i**) was not caused by a significant cell cycle arrest in any phase. The experiments presented here, using U-937 cells as a model, supported apoptosis as the main cytotoxicity pathway induced by two specific guanidines, **6f** and **6i**, containing the chalcone skeleton. This was supported by an increase in the percentage of sub-G_1_ and annexin V-FITC positive cells, caspase activation, and PARP cleavage. During apoptosis, caspases cause PARP cleavage and inactivation [9]. PARP protein is normally involved in DNA repair and is a known substrate for caspase-3. Moreover, these guanidines induced a concentration-dependent release of mitochondrial cytochrome *c* and this was in accordance with the activation of caspase-9 and caspase-3 determined by the enzymatic analysis and the immunoblots experiments. Mitochondrial outer membrane permeabilization is the critical step for the intrinsic pathway of apoptosis [10]. This pathway involves the cytosolic release of apoptogenic factors that normally reside in the mitochondrial intermembrane space such as cytochrome *c* [11]. These results suggest that cell death induction triggered by these specific compounds occurs via the intrinsic apoptotic pathway.

It is known that overexpression of Bcl-2 protein protects the cells because it prevents the release of the pro-apoptotic protein cytochrome *c* from the mitochondria to the cytosol, apoptosome formation, and the consequent activation of caspase-9 [12]. Bcl-2 is frequently overexpressed in lymphomas and leukemias [13]. Therefore, this protein is a potential target in therapy. Previous reports have shown that multisite Bcl-2 phosphorylation induced by anti-mitotic drugs may inhibit Bcl-2 [14]. For example, helenalin, a naturally occurring sesquiterpene lactone, is able to induce cell death in cells overexpressing Bcl-2 and this effect could be due to the inactivation of Bcl-2 protein or, alternatively, this compound could trigger a pathway that bypasses mitochondria [15]. In addition, etoposide, but not staurosporine, bypasses the chemoresistance conferred by Bcl-2 in Hep3B hepatoma cells [16]. Our experiments demonstrate that guanidines are cytotoxic against human leukemia U-937 cells that overexpress Bcl-2. These results strongly suggest that selected guanidines kill cancer cells mainly through induction of apoptotic cell death. Future studies will be necessary to determine whether additional pathways of cell death are involved in U-937 cells.

## 4. Materials and Methods

### 4.1. Reagents

Compounds used as starting material and reagents were obtained from Aldrich Chemical Co. (St. Louis, MO, USA) or other chemical companies and utilized without further purification. ^1^H and ^13^C NMR spectra were obtained on a Bruker Ascen 500 spectrometer model with standard pulse sequences operating at 500 MHz in ^1^H and 125 MHz in ^13^C NMR. CDCl_3_ was used as the solvent. Chemical shifts (δ) are given in ppm upfield from tetramethylsilane as internal standard, and coupling constants (*J*) are reported in hertz. EIMS and HREIMS were recorded on a Micromass model Autospec (70 eV) spectrometer. Column chromatography was carried out on silica gel 60 (Merck 230–400 mesh) and analytical thin layer chromatography (TLC) was performed using silica gel aluminum sheets. The starting materials 1-(2-(benzyloxy)-6-hydroxyphenyl)ethan-1-one (**1**) and 4-azidobenzaldehyde (**2**) were synthesized following the procedures described in the literature [17,18]. Acrylamide, bisacrylamide, ammonium persulfate, and *N*,*N*,*N′*,*N′*-tetramethylethylenediamine were from Bio-Rad (Hercules, CA, USA). Antibodies for caspase-3 (#ADI-AAP-113, 1:2000 dilution) and for caspase-9 (#9502) were purchased from Stressgen-ENZO (Victoria, BC, Canada) and Cell Signaling Technology (Beverly, MA, USA), respectively. Anti-PARP (poly(ADP-ribose) polymerase, #551024, 1:5000 dilution) and anti-cytochrome *c* (#556433, 1:1000 dilution) were from BD Pharmingen (San Diego, CA, USA). Monoclonal anti-β-actin (#A2228, 1:3000 dilution) and anti-tubulin (#2125, 1:1000 dilution) were from Sigma (Saint Louis, MO, USA) and Cell Signaling Technology (Beverly, MA, USA), respectively. Horseradish peroxidase-conjugated secondary antibodies (NA9310 and NA9340, 1:10,000 dilution) were from GE Healthcare Bio-Sciences AB (Little Chalfont, U.K.). PVDF membranes were from Millipore (Temecula, CA). All other chemicals were obtained from Sigma (Saint Louis, MO, USA).

### 4.2. General Procedure for the Synthesis of Hybrid Molecules

#### 4.2.1. Synthesis of Chalcone 3

A mixture of the 1-(2-(benzyloxy)-6-hydroxyphenyl)ethan-1-one (5–10 mmol, 1 equiv.) and the corresponding 4-azidobenzaldehyde (1 equiv.) in EtOH (20–40 mL) was stirred at room temperature and a 50% aqueous solution of NaOH (5–8 mL) was added. The reaction mixture was stirred at room temperature until the starting materials were consumed. HCl (10%) was then added until neutrality was reached. Precipitated chalcone was filtrated and purified using column chromatography (60% yield).

#### 4.2.2. General Method One-Pot Synthesis of Guanidines (**6a**–**6k**)

Triphenylphosphine (1.1 equiv.) was added at room temperature to a solution of azido-chalcone 3 (1 equiv.) in CH_2_Cl_2_. After 60 min, the isocyanate reagent (1.1 equiv.) was added. The reaction was followed by TLC until total consumption of the iminophosphorane. The appropriated amine (2 equiv.) was then added and the mixture was allowed to react for an additional 1 h. CH_2_Cl_2_ was removed in vacuo, and the residue was purified on silica gel MeOH–CH_2_Cl_2_ (5:95) to give the desired product a 40–75% yield. In some cases, additional preparative TLC developed with CH_2_Cl_2_–MeOH (95:5) was required. ^1^H-NMR, ^13^C-NMR and mass spectra of synthetic compounds are shown in Figures S1–S48 in Supplementary Materials.

### 4.3. Cell Culture and Cytotoxicity Assays

U-937, HL-60, MOLT-3, NALM-6, and SK-MEL-1 cells were from DSMZ (German Collection of Microorganisms and Cell Cultures, Braunschweig, Germany). U-937/Bcl-2 cells were kindly provided by Dr. Jacqueline Bréard (INSERM U749, Faculté de Pharmacie Paris-Sud, Châtenay-Malabry, France). The U-937 is a pro-monocytic, human promyelocytic leukemia cell line that was isolated from a histiocytic lymphoma. HL-60 is a human acute myeloid leukemia cell line. MOLT-3 is a human acute lymphoblastic leukemia cell line. NALM-6 is a human B cell precursor leukemia. SK-MEL-1 is a human melanoma cell line growing singly or in clumps (occasional giant, polynucleated cell) in suspension. Cells were cultured in RPMI 1640 medium containing 10% (*v*/*v*) heat-inactivated fetal bovine serum, 100 μg/mL streptomycin, and 100 U/mL penicillin, incubated at 37 °C in a humidified atmosphere containing 5% CO_2_ as described [19]. The doubling times of the cell lines were 24 h in HL-60, 30 h in U-937, U-937/Bcl-2, 40 h in MOLT-3 and NALM-6, and several days in SK-MEL-1 cells. Human peripheral blood mononuclear cells (PBMC) were isolated from heparin-anticoagulated blood of healthy volunteers by centrifugation with Ficoll-Paque Plus (GE Healthcare Bio-Sciences AB, Uppsala, Sweden). PBMC were also stimulated with phytohemagglutinin (2 µg/mL) for 48 h before the experimental treatment. The trypan blue exclusion method was used for counting the cells by a hemocytometer with 95% viability in all the experiments. Compounds were dissolved in dimethyl sulfoxide (DMSO) and kept under dark conditions at 25 °C. Before each experiment, the compounds were dissolved in culture media at 37 °C and the final concentration of DMSO did not exceed 0.3% (*v*/*v*). The cytotoxicity of the synthetic compounds was evaluated by colorimetric 3-(4,5-dimethyl-2-thiazolyl-)-2,5-diphenyl-2*H*-tetrazolium bromide (MTT) assay as previously described [20]. Briefly, cells (5000 per well) were incubated with increasing concentrations of compounds for 72 h into a 96-well plate. Then, the supernatants were removed and 0.5 mg/mL MTT was added and incubated for 4 h at 37 °C. Sodium dodecyl sulfate (10% *w*/*v*) in 0.05 M HCl was added to solubilize the reaction products and the absorbance was measured at 570 nm with a reference wavelength of 570 nm using an ELISA reader (Bio-Rad, Hercules, CA, USA). The IC_50_ values (concentrations inducing a 50% inhibition of cell viability) were determined graphically for each experiment by a nonlinear regression using the curve fitting routine implemented within the software Prism 5.0 (GraphPad, La Jolla, CA, USA).

### 4.4. Quantification of Sub-G_1_ Cells and Analysis of Cell Cycle and Apoptosis by Flow Cytometry

Cells (2.5 × 10^5^) were centrifuged for 10 min at 500× *g*, washed with cold PBS, fixed with ice-cold 75% ethanol and stored at −20 °C overnight. Samples were then centrifuged at 500× *g* for 10 min at 4 °C, washed with PBS, resuspended in 200 μL of PBS containing 100 μg/mL RNase and 50 μg/mL propidium iodide and incubated for 1 h in the dark. The DNA content was analyzed by flow cytometry with a BD FACSVerse^TM^ cytometer (BD Biosciences, San Jose, CA, USA). Apoptosis was also evaluated by flow cytometric analysis of double-staining annexin V-FITC and propidium iodide cells as previously described [21].

### 4.5. Assay of Caspase Activity

Caspase activity was determined by measuring proteolytic cleavage of specific chromogenic substrates as previously described [22]. Briefly, cells were treated with the specified guanidines for 24 h, harvested by centrifugation (1000× *g* for 5 min at 4 °C), washed twice with PBS, resuspended in lysis buffer (50 mM HEPES, pH 7.4, 0.1 mM EDTA, 1 mM dithiothreitol, and 0.1% Chaps), spun (17,000× *g* for 10 min at 4 °C) and the supernatants were normalized by protein concentration and used to determine enzyme activity. The net increase in absorbance at 405 nm after incubation at 37 °C was indicative of caspase activity. The colorimetric substrates were DEVD-*p*NA (for caspase-3 like protease activity), IETD-*p*NA (for caspase-8 activity), and LEHD-*p*NA (for caspase-9 activity).

### 4.6. Western Blot Analysis

For whole cell lysates, cells were harvested by centrifugation (500× *g*, 10 min, 4 °C), washed twice with PBS, and pellets were resuspended in lysis buffer (1% Triton X-100, 10 mM sodium fluoride, 2 mM EDTA, 20 mM Tris-HCl (pH 7.4), 2 mM tetrasodium pyrophosphate, 10% glycerol, 137 mM NaCl, and 20 mM sodium β-glycerophosphate), with the protease inhibitors phenylmethylsulfonyl fluoride (PMSF, 1 mM), aprotinin, leupeptin, and pepstatin A (5 μg/mL each) and kept on ice for 15 min. Cells were sonicated on ice 5 times (5 s each, with intervals between each sonication of 5 s) with a Braun Labsonic 2000 microtip sonifier and centrifuged (11,000× *g*, 10 min, 4 °C). For subcellular fractionation, cells were washed twice with PBS and then resuspended in ice-cold buffer (20 mM HEPES (pH 7.5), 250 mM sucrose, 10 mM KCl, 1.5 mM MgCl_2_, 1 mM EDTA, 1 mM EGTA, and 1 mM dithiothreitol containing protease inhibitors (0.1 mM PMSF and 1 µg/mL leupeptin, aprotinin, and pepstatin A)). After 15 min on ice, cells were lysed by pushing them several times through a 29-gauge needle, and the lysate was centrifuged at 1000× *g* for 5 min at 4 °C. The resulting supernatant fraction was centrifuged at 15,000× *g* for 20 min at 4 °C, and the resulting supernatant was used as the cytosolic fraction. The Bradford method was used to determine protein concentration. The samples loaded in sodium dodecyl sulfate-polyacrylamide gel (from 7.5 to 15% depending on the molecular weight of interest) were prepared with the same protein amount and boiled for 5 min. The proteins were transferred to poly(vinylidene difluoride) membranes for 20 h at 20 V. The membranes were blocked with 10% nonfat milk in Tris-buffered saline (50 mM Tris-HCl (pH 7.4), 150 mM NaCl) containing 0.1% Tween-20 (TBST) for 1 h, followed by incubation with specific antibodies against caspase-3, PARP, caspase-9, β-actin, tubulin, and cytochrome *c* overnight at 4 °C. Membranes were washed three times with TBST and incubated for 1 h with the specific secondary antibody and the antigen-antibodies complexes were visualized by enhanced chemiluminescence using the manufacturer’s protocol.

### 4.7. Statistical Analysis

Statistical differences between means were tested using (i) Student’s *t*-test (two samples) or (ii) one-way analysis of variance (ANOVA) (three or more samples) with a posteriori pairwise comparisons of means carried out using Tukey’s test. A significance level of *p* < 0.05 was used.

## 5. Conclusions

In summary, we designed and synthesized a series of trisubstituted guanidines containing a 1,3-diphenylpropenone core and different substituents on the guanidine functional group taking advantage of a one-pot procedure involving three sequential reactions. The SARs revealed that (i) the presence of a piperazine ring in *p*-toluensulfonyl guanidines generated a more cytotoxic response than the corresponding isopropylamino, diisopropylamino, piperidine, morpholine, or phenylamino derivatives and (ii) the presence of a piperidine ring in *N*-phenylguanidines enhanced the cytotoxicity in comparison with the corresponding diisopropylamino, morpholine, and *N*-methylpiperazine derivatives. The most cytotoxic compounds were the *p*-toluensulfonylguanidine containing a *N*-methylpiperazine and the *N*-methyl-*N*-phenylguanidine containing a piperidine ring against the human leukemia cells. Both compounds showed less cytotoxicity against human peripheral blood mononuclear cells, suggesting that they may have potential therapeutic value. These compounds induce cell death by apoptosis, which was associated with cytochrome *c* release, caspase activation, and PARP cleavage while the over-expression of the anti-apoptotic protein Bcl-2 did not block cell viability inhibition.

## Figures and Tables

**Table 1:** Azidochalcone, isocyanates, and amines as starting reagents to generate the corresponding guanidine–chalcone hybrids.

Chalcone	Isocyanate	Amine	Product
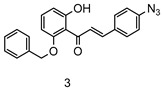	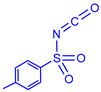	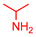	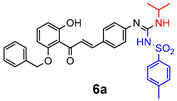
		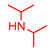	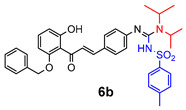
		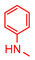	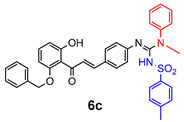
		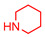	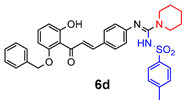
		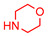	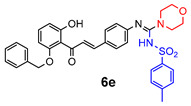
		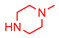	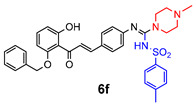
		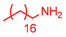	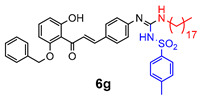
	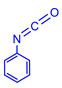	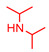	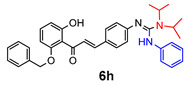
		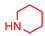	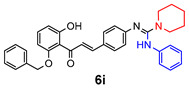
		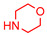	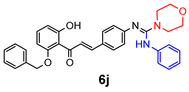
		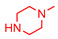	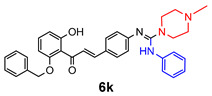

**Table 2:** Effects on cell viability of hybrid compounds on human cancer cells.

	IC_50_ (µM)					
Compound	U-937	HL-60	MOLT-3	NALM-6	U-937/Bcl-2	SK-MEL-1
**3**	-	-	7.4 ± 0.4	11.6 ± 1.6	-	-
**6a**	11.4 ± 6.5	3.7 ± 1.6	2.9 ± 1.1	2.9 ± 0.5	2.9 ± 0.2	-
**6b**	18.5 ± 9.6	20.5 ± 9.5	2.0 ± 0.7	27.0 ± 0.8	11.7 ± 7.0	-
**6c**	9.5 ± 5.3	4.2 ± 0.5	2.5 ± 0.2	9.9 ± 3.1	4.2 ± 1.2	-
**6d**	-	28.5 ± 14.3	3.3 ± 0.6	6.6 ± 1.2	24.6 ± 5.4	-
**6e**	5.4 ± 2.1	3.8 ± 1.1	3.2 ± 1.5	3.1 ± 0.5	2.3 ± 0.9	14.3 ± 5.7
**6f**	1.6 ± 0.6	1.6 ± 1.0	1.5 ± 0.0	2.7 ± 0.0	2.2 ± 0.8	4.5 ± 0.4
**6g**	-	-	-	-	-	-
**6h**	10.6 ± 2.6	5.2 ± 0.9	4.3 ± 0.5	5.4 ± 0.2	14.0 ± 6.6	5.2 ± 1.0
**6i**	3.5 ± 1.1	3.2 ± 0.6	7.1 ± 1.3	10.8 ± 0.5	5.1 ± 1.8	13.8 ± 1.2
**6j**	8.6 ± 1.1	6.2 ± 1.7	1.8 ± 0.5	4.1 ± 0.5	11.7 ± 3.4	5.4 ± 0.8
**6k**	14.0 ± 1.1	11.4 ± 5.5	21.7 ± 6.5	24.8 ± 3.3	14.0 ± 5.1	-
**Etoposide**	1.8 ± 0.2	0.6 ± 0.2	0.2 ± 0.1	ND	ND	9.0 ± 3.0
**Doxorubicin**	0.1 ± 0.0	ND	ND	ND	ND	0.3 ± 0.1

Cells were cultured for 72 h and the IC_50_ values were determined as described in Section 4. Data are expressed as means ± SEM from the dose-response curves of 3–5 independent experiments with three determinations in each. - means not active, IC_50_ values > 30 µM. ND, not determined.

**Table 3:** Effect of guanidines on cell cycle phases distribution in U-937 leukemia cells.

	%Sub-G_1_	%G_1_	%S	%G_2_-M
Control	2.4 ± 0.4	54.4 ± 0.4	29.0 ± 0.7	14.2 ± 0.2
**6c**	23.5 ± 1.2 *	37.4 ± 0.3 *	27.0 ± 0.7	11.4 ± 0.4
**6e**	11.9 ± 0.2 *	36.5 ± 2.3 *	23.6 ± 0.5 *	27.3 ± 2.0 *
**6f**	25.1 ± 2.6 *	35.2 ± 3.8 *	28.3 ± 0.2	10.8 ± 1.6
**6h**	7.2 ± 1.1 *	53.7 ± 0.4	29.5 ± 0.3	9.2 ± 0.5 *
**6i**	31.6 ± 4.5 *	35.9 ± 2.1 *	23.6 ± 2.4 *	8.4 ± 0.1 *
**6j**	4.7 ± 0.6	51.3 ± 1.1	32.0 ± 0.4	11.5 ± 0.1

Cells were cultured with 10 µM of the indicated compounds for 24 h and the cell cycle phase distributions were determined by flow cytometry. The values are means ± S.E. of two independent experiments with three determinations in each. Asterisks indicate a significant difference (*p* < 0.05) compared with the corresponding controls.

## Data Availability

Data is contained within the article or Supplementary Materials.

## Supplementary Materials

The following supporting information can be downloaded at: https://www.mdpi.com/article/10.3390/ijms232415518/s1. The following are available online, spectral and analytical data for hybrid compounds.
