# Supplementary material for: Guanidine Derivatives Containing the Chalcone Skeleton Are Potent Antiproliferative Compounds against Human Leukemia Cells
Source: Int J Mol Sci. 2022 Dec 8;23(24):15518. doi: 10.3390/ijms232415518 (PMC9779571; doi:10.3390/ijms232415518)
Supplement: Supplementary file 1 [file ijms-23-15518-s001.zip › ijms-2036040-supplementary.pdf]

Supplementary Materials

Guanidine Derivatives Containing the Chalcone Skeleton Are Potent Antiproliferative Compounds against Human Leukemia Cells

Francisco Estévez-Sarmiento [1,*], Ester Saavedra [1,2], Ignacio Brouard [3], Jesús Peyrac [3], Judith Hernández-Garcés [4], Celina García [4], José Quintana [1] and Francisco Estévez [1]

1 Departamento de Bioquímica y Biología Molecular, Fisiología, Genética e Inmunología, Instituto Universitario de Investigaciones Biomédicas y Sanitarias (IUIBS), Grupo de Química Orgánica y Bioquímica, Universidad de Las Palmas de Gran Canaria, Unidad Asociada al Consejo Superior de Investigaciones Científicas (CSIC), 35016 Las Palmas de Gran Canaria, Spain
2 Instituto Canario de Investigación del Cáncer (ICIC), 35016 Las Palmas de Gran Canaria, Spain
3 Instituto de Productos Naturales y Agrobiología, Consejo Superior de Investigaciones Científicas, 38206 La Laguna, Spain
4 Instituto Universitario de Bio-orgánica AG, Departamento de Química Orgánica, Universidad de La Laguna (Tenerife), 38206 San Cristóbal de La Laguna, Spain

* Correspondence: francisco.estevez103@alu.ulpgc.es or festevez1985@gmail.com;
Tel.: +34-928-451443; Fax: +34-928-451441

Table of Contents

1. NMR spectroscopic and mass-spectrometric data

2. NMR and Mass Spectra:

Figure S1: ^{1}H-NMR (500 MHz, $CDCl_3$) Spectrum of Compound **3**

Figure S2: ^{13}C-NMR (125 MHz, $CDCl_3$) Spectrum of Compound **3**

Figure S3: HRESI-MS Spectrum of Compound **3**

Figure S4: ESI-MS Spectrum of Compound **3**

Figure S5: ^{1}H-NMR (500 MHz, $CDCl_3$) Spectrum of Compound **6a**

Figure S6: ^{13}C-NMR (125 MHz, $CDCl_3$) Spectrum of Compound **6a**

Figure S7: HRESI-MS Spectrum of Compound **6a**

Figure S8: ESI-MS Spectrum of Compound **6a**

Figure S9: ^{1}H -NMR (500 MHz, $CDCl_3$) Spectrum of Compound **6b**

Figure S10: ^{13}C-NMR (125 MHz, $CDCl_3$) Spectrum of Compound **6b**

Figure S11: HRESI-MS Spectrum of Compound **6b**

Figure S12: ESI-MS Spectrum of Compound **6b**

Figure S13: ^{1}H-NMR (500 MHz, $CDCl_3$) Spectrum of Compound **6c**

Figure S14: ^{13}C-NMR (125 MHz, $CDCl_3$) Spectrum of Compound **6c**

Figure S15: HRESI-MS Spectrum of Compound **6c**

Figure S16: ESI-MS Spectrum of Compound **6c**

Figure S17: ^{1}H-NMR (500 MHz, $CDCl_3$) Spectrum of Compound **6d**

Figure S18: ^{13}C-NMR (125 MHz, $CDCl_3$) Spectrum of Compound **6d**

Figure S19: HRESI-MS Spectrum of Compound **6d**

Figure S20: ESI-MS Spectrum of Compound **6d**

Figure S21: ^{1}H-NMR (500 MHz, $CDCl_3$) Spectrum of Compound **6e**

Figure S22: ^{13}C-NMR (125 MHz, $CDCl_3$) Spectrum of Compound **6e**

Figure S23: HRESI-MS Spectrum of Compound **6e**

Figure S24: ESI-MS Spectrum of Compound **6e**

Figure S25: ^{1}H-NMR (500 MHz, $CDCl_3$) Spectrum of Compound **6f**

Figure S26: ^{13}C-NMR (125 MHz, $CDCl_3$) Spectrum of Compound **6f**

Figure S27: HRESI-MS Spectrum of Compound **6f**

Figure S28: ESI-MS Spectrum of Compound **6f**

Figure S29: ^{1}H-NMR (500 MHz, $CDCl_3$) Spectrum of Compound **6g**

Figure S30: ^{13}C -NMR (125 MHz, $CDCl_3$) Spectrum of Compound **6g**

Figure S31: HRESI-MS Spectrum of Compound **6g**

Figure S32: ESI-MS Spectrum of Compound **6g**

Figure S33: ^{1}H-NMR (500 MHz, $CDCl_3$) Spectrum of Compound **6h**

Figure S34: ^{13}C-NMR (125 MHz, $CDCl_3$) Spectrum of Compound **6h**

Figure S35: HRESI-MS Spectrum of Compound **6h**

Figure S36: ESI-MS Spectrum of Compound **6h**

Figure S37: ^{1}H-NMR (500 MHz, $CDCl_3$) Spectrum of Compound **6i**

Figure S38: ^{13}C-NMR (125 MHz, $CDCl_3$) Spectrum of Compound **6i**

Figure S39: HRESI-MS Spectrum of Compound **6i**

Figure S40: ESI-MS Spectrum of Compound **6i**

Figure S41: ^{1}H-NMR (500 MHz, $CDCl_3$) Spectrum of Compound **6j**

Figure S42: ^{13}C-NMR (125 MHz, $CDCl_3$) Spectrum of Compound **6j**

Figure S43: HRESI-MS Spectrum of Compound **6j**

Figure S44: ESI-MS Spectrum of Compound **6j**

Figure S45: ^{1}H-NMR (500 MHz, $CDCl_3$) Spectrum of Compound **6k**

Figure S46: ^{13}C-NMR (125 MHz, $CDCl_3$) Spectrum of Compound **6k**

Figure S47: HRESI-MS Spectrum of Compound **6k**

Figure S48: ESI-MS Spectrum of Compound **6k**

1. NMR spectroscopic and mass-spectrometric data

*(E)-3-(4-azidophenyl)-1-(2-(benzyloxy)-6-hydroxyphenyl)prop-2-en-1-one (**3**):*

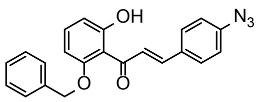

^{1}H-NMR (500 MHz, $CDCl_3$) δ 13.53 (s, 1H); 7.82 (d, *J* = 15.6 Hz, 1H); 7.69 (d, *J* = 15.6 Hz, 1H); 7.55-7.46 (m, 2H); 7.46-7.36 (m, 4H); 7.11-7.02 (m, 2H); 6.88-6.79 (m, 2H); 6.65 (dd, *J* = 8.4, 1.0 Hz, 1H); 6.54 (dd, *J* = 8.3, 1.1 Hz, 1H); 5.12 (s, 2H). ^{13}C-NMR (125 MHz, $CDCl_3$) δ 194.2, 165.6, 160.2, 142.1, 141.6, 136.1, 135.6, 132.0, 130.0, 128.9, 128.7, 128.6, 127.2, 119.2, 111.7, 111.4, 102.2, 71.4. HRMS (ESI-FT-ICR) m/z: 394.1165 $[M+Na]^+$; calcd. for $C_{22}H_{17}N_3NaO_3$: 394.1168.

*N-((Z)-N′-(4-((E)-3-(2-(benzyloxy)-6-hydroxyphenyl)-3-oxoprop-1-en-1-yl)phenyl)-N-isopropylcarbamimidoyl)-4-methylbenzenesulfonamide (**6a**):*

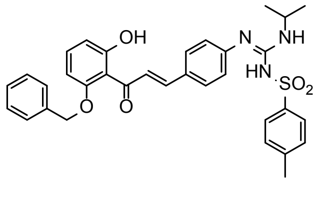

^{1}H-NMR (500 MHz, $CDCl_3$) δ 13.50 (s, 1H), 9.01 (s, 2H), 7.91-7.79 (m, 3H), 7.67 (d, *J* = 15.5 Hz, 1H), 7.51 (d, *J* = 6.4 Hz, 2H), 7.48-7.36 (m, 4H), 7.29 (d, *J* = 8.0 Hz, 2H), 7.05 (d, *J* = 8.2 Hz, 2H), 6.95 (d, *J* = 8.0 Hz, 2H), 6.66 (d, *J* = 8.4 Hz, 1H), 6.56 (d, *J* = 8.2 Hz, 1H), 5.13 (s, 2H), 4.58 (s, 1H), 4.14 (bm, 1H), 2.42 (s, 3H), 1.12 (d, *J* = 6.5 Hz, 6H). ^{13}C-NMR (125 MHz, $CDCl_3$) δ 194.1, 165.7, 160.3, 152.6, 142.1, 141.4, 140.9, 136.9, 136.3, 135.6, 133.8, 130.0, 129.2, 128.9, 128.9, 128.7, 128.4, 126.0, 124.9, 111.6, 111.5, 102.2, 71.5, 43.7, 22.7, 21.5. HRMS (ESI-FT-ICR) m/z: 606.2034 $[M+Na]^+$; calcd. for $C_{33}H_{33}N_3NaO_5S$: 606.2039. (65% yield).

*N-((Z)-N′-(4-((E)-3-(2-(benzyloxy)-6-hydroxyphenyl)-3-oxoprop-1-en-1-yl)phenyl)-N,N-diisopropylcarbamimidoyl)-4-methylbenzenesulfonamide (**6b**):*

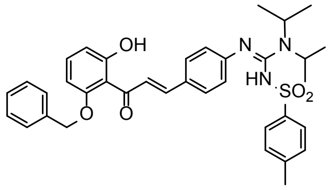

^{1}H-NMR (500 MHz, $CDCl_3$) δ 13.61 (s, 1H), 8.16 (s, 1H), 7.82 (d, *J* = 15.6 Hz, 1H), 7.73 (d, *J* = 8.2 Hz, 2H), 7.67 (d, *J* = 15.5 Hz, 1H), 7.55-7.48 (m, 2H), 7.41 (d, *J* = 8.4 Hz, 1H), 7.38 (dd, *J* = 4.9, 2.2 Hz, 3H), 7.15 (d, *J* = 7.6 Hz, 2H), 6.92 (d, *J* = 8.5 Hz, 2H), 6.66 (dd, *J* = 8.4, 1.0 Hz, 1H), 6.61-6.52 (m, 3H), 5.12 (s, 2H), 3.68 (p, *J* = 6.7 Hz, 2H), 2.35 (s, 3H), 1.18 (d, *J* = 6.7 Hz, 12H). ^{13}C-NMR (125 MHz, $CDCl_3$) δ. 194.2, 165.6, 160.2, 155.3, 142.4, 142.1, 142.0, 140.4, 136.1, 135.7, 131.5, 129.7, 129.1, 128.9, 128.8, 128.6, 126.9, 126.4, 121.5, 111.6, 111.5, 102.2, 71.4, 49.2, 21.5, 20.8. HRMS (ESI-FT-ICR) m/z: 648.2509 $[M+Na]^+$; calcd. for $C_{36}H_{39}N_3NaO_5S$: 648.2508. (72% yield).

*N-((Z)-N′-(4-((E)-3-(2-(benzyloxy)-6-hydroxyphenyl)-3-oxoprop-1-en-1-yl)phenyl)-N-methyl-N-phenylcarbamimidoyl)-4-methylbenzenesulfonamide (**6c**):*

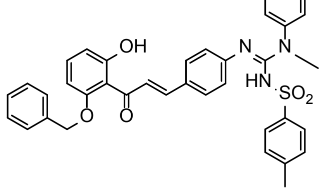

^{1}H-NMR (500 MHz, $CDCl_3$) δ 13.47 (s, 1H); 8.76 (s, 1H); 7.84 (d, *J* = 8.3 Hz, 1H); 7.82 (d, *J* = 8.3 Hz, 1H); 7.70 (d, *J* = 15.5 Hz, 1H); 7.53 (d, *J* = 15.6 Hz, 1H); 7.50-7.43 (m, 2H); 7.39 (t, *J* = 8.3 Hz, 1H); 7.36-7.33 (m, 2H); 7.33-7.29 (m, 1H); 7.29-7.26 (m, 2H); 7.04 (dd, *J* = 8.4, 7.2 Hz, 2H); 6.99-6.90 (m, 1H); 6.85 (dd, *J* = 8.5, 1.2 Hz, 2H); 6.69 (d, *J* = 8.6 Hz, 2H); 6.64 (d, *J* = 8.5 Hz, 1H); 6.53 (d, *J* = 8.3 Hz, 1H); 6.39 (d, *J* = 8.6 Hz, 2H); 5.11 (s, 2H); 3.31 (s, 3H); 2.43 (s, 3H). ^{13}C-NMR (125 MHz, $CDCl_3$) δ 194.2, 165.5, 160.2, 156.3, 143.3, 142.6, 142.1, 140.2, 139.2, 135.6, 131.9, 129.7, 129.4, 129.0, 128.8, 128.6, 128.5, 127.3, 126.5, 126.4, 126.4, 125.7, 123.6, 111.7, 111.5, 102.3, 71.4, 41.1, 21.5. HRMS (ESI-FT-ICR) m/z: 654.2035 $[M+Na]^+$; calcd. for $C_{37}H_{33}N_3NaO_5S$: 654.2039. (60% yield).

*(Z)-N′-(4-((E)-3-(2-(benzyloxy)-6-hydroxyphenyl)-3-oxoprop-1-en-1-yl)phenyl)-N-tosylpiperidine-1-carboximidamide (**6d**):*

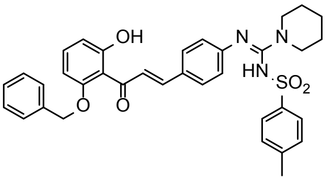

^{1}H-NMR (500 MHz, $CDCl_3$) δ 13.59 (s, 1H), 8.61 (s, 1H), 7.82 (d, *J* = 15.6 Hz, 1H), 7.74-7.69 (m, 2H), 7.66 (d, *J* = 15.6 Hz, 1H), 7.54-7.48 (m, 2H), 7.42-7.35 (m, 3H), 7.15 (d, *J* = 8.1 Hz, 2H), 6.95 (d, *J* = 8.6 Hz, 2H), 6.65 (dd, *J* = 8.4, 1.0 Hz, 1H), 6.60-6.53 (m, 3H), 5.13 (s, 2H), 3.28 (t, *J* = 5.3 Hz, 4H), 2.35 (s, 3H), 1.65 (s, 1H), 1.58 (m, 2H), 1.53 (m, 4H). ^{13}C-NMR (125 MHz, $CDCl_3$) δ 194.2, 165.6, 160.2, 154.8, 142.3, 142.2, 140.8, 140.3, 140.3, 136.1, 135.7, 131.1, 129.8, 129.3, 128.9, 128.7, 128.5, 126.9, 126.2, 119.8, 111.5, 102.2, 71.4, 48.3, 25.3, 24.0, 21.4. HRMS (ESI-FT-ICR) m/z: 632.2193 $[M+Na]^+$; calcd. for $C_{35}H_{35}N_3NaO_5S$: 632.2195. (68% yield).

*(Z)-N′-(4-((E)-3-(2-(benzyloxy)-6-hydroxyphenyl)-3-oxoprop-1-en-1-yl)phenyl)-N-tosylmorpholine-4-carboximidamide (**6e**):*

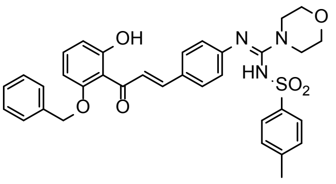

^{1}H-NMR (500 MHz, $CDCl_3$) δ 13.53 (s, 1H); 8.75 (s, 1H); 7.83 (d, *J* = 15.6 Hz, 1H); 7.73 (d, *J* = 8.2 Hz, 2H); 7.66 (d, *J* = 15.5 Hz, 1H); 7.56-7.47 (m, 2H); 7.44-7.34 (m, 4H); 7.19 (d, *J* = 8.1 Hz, 2H); 6.99 (d, *J* = 8.6 Hz, 2H); 6.70-6.60 (m, 3H); 6.55 (dd, *J* = 8.3, 1.0 Hz, 1H); 5.13 (s, 2H); 3.61 (t, *J* = 4.8 Hz, 4H); 3.34 (d, *J* = 4.9 Hz, 4H); 2.37 (s, 3H). ^{13}C-NMR (125 MHz, $CDCl_3$) δ 194.1, 165.6, 160.2, 155.0, 142.6, 141.8, 140.0, 136.2, 135.7, 131.8, 129.9, 129.4, 128.9, 128.7, 128.5, 127.4, 126.3, 120.3, 111.7, 111.5, 102.2, 71.4, 66.0, 47.3, 21.5. HRMS (ESI-FT-ICR) m/z: 634.1989 $[M+Na]^+$; calcd. for $C_{34}H_{33}N_3NaO_6S$: 634.1988. (73% yield).

*(Z)-N′-(4-((E)-3-(2-(benzyloxy)-6-hydroxyphenyl)-3-oxoprop-1-en-1-yl)phenyl)-4-methyl-N-tosylpiperazine-1-carboximidamide (**6f**):*

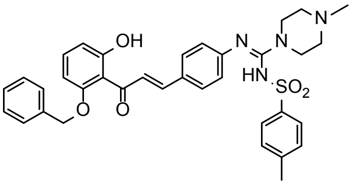

^{1}H-NMR (500 MHz, $CDCl_3$) δ 13.54 (s, 1H); 8.70 (s, 1H); 7.82 (d, *J* = 15.5 Hz, 1H); 7.72 (d, *J* = 8.3 Hz, 2H); 7.66 (d, *J* = 15.6 Hz, 1H); 7.56-7.46 (m, 2H); 7.43-7.35 (m, 4H); 7.17 (d, *J* = 8.1 Hz, 2H); 6.96 (d, *J* = 8.6 Hz, 2H); 6.65 (dd, *J* = 8.4, 1.0 Hz, 1H); 6.63-6.59 (m, 2H); 6.55 (dd, *J* = 8.3, 1.0 Hz, 1H); 5.12 (s, 2H); 3.36 (t, *J* = 5.0 Hz, 4H); 2.35 (d, *J* = 7.5 Hz, 7H); 2.26 (s, 3H). ^{13}C-NMR (125 MHz, $CDCl_3$) δ 193.1, 164.6, 159.2, 153.8, 141.4, 141.0, 139.3, 139.1, 135.1, 134.7, 130.4, 128.8, 128.3, 127.8, 127.7, 127.5, 126.2, 125.2, 119.1, 110.6, 110.4, 101.2, 70.4, 53.0, 45.8, 44.7, 20.4. HRMS (ESI-FT-ICR) m/z: 623.2317 [M-H]; calcd. for $C_{35}H_{35}N_4O_5S$: 623.2328. (75% yield).

*N-((Z)-N′-(4-((E)-3-(2-(benzyloxy)-6-hydroxyphenyl)-3-oxoprop-1-en-1-yl)phenyl)-N-octadecylcarbamimidoyl)-4-methylbenzenesulfonamide (**6g**):*

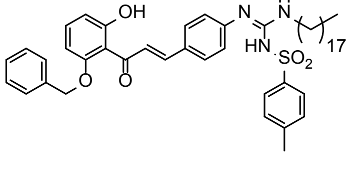

^{1}H-NMR (500 MHz, $CDCl_3$) δ 13.48 (s, 1H), 9.08 (s, 1H), 7.89-7.80 (m, 3H), 7.67 (d, *J* = 15.6 Hz, 1H), 7.55-7.48 (m, 2H), 7.47-7.36 (m, 4H), 7.28 (d, *J* = 8.0 Hz, 2H), 7.09-7.02 (m, 2H), 6.97 (s, 2H), 6.67 (dd, *J* = 8.4, 1.0 Hz, 1H), 6.56 (dd, *J* = 8.4, 1.1 Hz, 1H), 5.13 (s, 2H), 3.29 (q, *J* = 6.6 Hz, 2H), 2.42 (s, 3H), 1.45 (s, 2H), 1.24 (d, *J* = 12.2 Hz, 30H), 0.88 (t, *J* = 6.9 Hz, 3H). ^{13}C-NMR (125 MHz, $CDCl_3$) δ 194.16, 165.71, 160.32, 153.47, 142.16, 141.3, 140.89, 136.8, 136.35, 135.64, 134.1, 130.04, 129.26, 128.98, 128.87, 128.73, 126.09, 125.1, 111.65, 111.56, 102.25, 71.51, 41.77, 31.95, 30.95, 29.73, 29.68, 29.61, 29.50,

29.39, 29.34, 29.23, 26.75, 22.72, 21.52, 14.15. HRMS (ESI-FT-ICR) m/z: 816.4393 $[M+Na]^+$; calcd. for $C_{48}H_{63}N_3O_5NaS$: 816.4386. (72% yield).

*(E)-2-(4-((E)-3-(2-(benzyloxy)-6-hydroxyphenyl)-3-oxoprop-1-en-1-yl)phenyl)-1,1-diisopropyl-3-phenylguanidine (**6h**):*

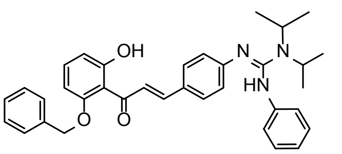

^{1}H-NMR (500 MHz, $CDCl_3$) δ 13.73 (s, 1H); 7.74 (d, *J* = 15.5 Hz, 1H); 7.69 (d, *J* = 15.5 Hz, 1H); 7.47 (dd, *J* = 8.0, 1.5 Hz, 2H); 7.35 (t, *J* = 8.3 Hz, 1H); 7.36-7.22 (m, 5H); 7.21-7.14 (m, 2H); 6.91 (m, 3H); 6.87-6.79 (m, 2H); 6.69-6.59 (m, 3H); 6.52 (d, *J* = 8.2 Hz, 1H); 5.10 (s, 2H); 3.91 (p, *J* = 6.8 Hz, 2H); 1.32 (d, *J* = 6.8 Hz, 12H). ^{13}C-NMR (125 MHz, $CDCl_3$) δ 194.2, 174.3, 165.5, 160.2, 150.4, 149.1, 144.4, 135.7, 135.5, 129.9, 129.0, 128.8, 128.5, 128.4, 127.9, 124.2, 122.0, 120.8, 119.6, 111.9, 111.4, 102.2, 71.3, 47.8, 21.5. HRMS (ESI-FT-ICR) m/z: 548.2914 [M]; calcd. for $C_{35}H_{38}N_3O_3$: 548.2913. (48% yield).

*(E)-N′-(4-((E)-3-(2-(benzyloxy)-6-hydroxyphenyl)-3-oxoprop-1-en-1-yl)phenyl)-N-phenylpiperidine-1-carboximidamide (**6i**):*

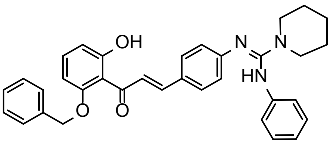

^{1}H-NMR (500 MHz, $CDCl_3$) δ 13.67 (s, 1H), 7.80 (d, *J* = 15.5 Hz, 1H), 7.73 (d, *J* = 15.5 Hz, 1H), 7.47 (d, *J* = 7.4 Hz, 2H), 7.36 (m, 2H), 7.20-7.34 (m, 5H), 7.06-6.84 (m, 5H), 6.77 (d, *J* = 8.1 Hz, 2H), 6.64 (dd, *J* = 8.4, 1.0 Hz, 1H), 6.53 (dd, *J* = 8.3, 1.0 Hz, 1H), 5.10 (s, 2H), 3.34 (s, 4H), 1.61 (brm, 6H). ^{13}C-NMR (125 MHz, $CDCl_3$) δ 194.2, 165.6, 160.2, 143.9, 135.7, 130.1, 129.4, 128.8, 128.8, 128.7, 128.5, 128.4, 124.9, 122.3, 111.8, 111.4, 102.1, 71.4, 47.7, 25.3, 24.6. HRMS (ESI-FT-ICR) m/z: 532.2604 [M]; calcd. for $C_{34}H_{34}N_3O_3$: 532.2600. (60% yield).

*(E)-N′-(4-((E)-3-(2-(benzyloxy)-6-hydroxyphenyl)-3-oxoprop-1-en-1-yl)phenyl)-N-phenylmorpholine-4-carboximidamide (**6j**):*

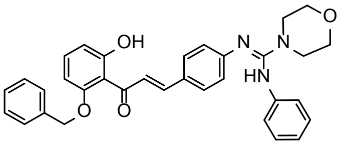

^{1}H-NMR (500 MHz, $CDCl_3$) δ 13.72 (s, 1H); 7.80 (d, *J* = 15.6 Hz, 1H), 7.71 (d, *J* = 15.5 Hz, 1H), 7.51-7.43 (m, 2H), 7.42-7.24 (m, 6H), 7.23-7.10 (m, 1H), 7.01 (m, 5H), 6.75 (d, J = 8.0 Hz, 2H), 6.64 (dd, *J* = 8.4, 1.0 Hz, 1H), 6.53 (dd, *J* = 8.3, 1.0 Hz, 1H), 5.10 (s, 2H), 3.72 (q, *J* = 4.7, 4.0 Hz, 4H), 3.42-3.32 (m, 4H). ^{13}C-NMR (125 MHz, $CDCl_3$) δ 194.2, 165.6, 160.2, 143.5, 135.8, 132.9, 132.1, 132.1, 132.0, 131.9, 131.9, 130.1, 129.5, 128.9, 128.7, 128.5, 128.5, 128.4, 122.9, 111.8, 111.4, 102.2, 71.4, 66.3, 47.0. HRMS (ESI-FT-ICR) m/z: 534.2397 [M]; calcd. for $C_{33}H_{32}N_3O_4$: 534.2393. (52% yield).

*(E)-N′-(4-((E)-3-(2-(benzyloxy)-6-hydroxyphenyl)-3-oxoprop-1-en-1-yl)phenyl)-4-methyl-N-phenylpiperazine-1-carboximidamide (**6k**):*

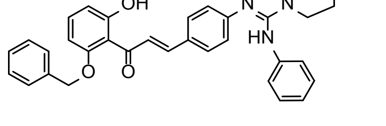

^{1}H-NMR (400 MHz, $CDCl_3$) δ 13.69 (s, 1H), 7.79 (d, *J* = 15.5 Hz, 1H), 7.70 (d, *J* = 15.5 Hz, 1H), 7.47 (d, *J* = 7.0 Hz, 2H), 7.40-7.15 (m, 7H), 7.06 (t, *J* = 7.4 Hz, 1H), 6.97 (m, 4H), 6.77 (d, *J* = 8.1 Hz, 2H), 6.63 (dd, *J* = 8.4, 1.0 Hz, 1H), 6.52 (dd, *J* = 8.4, 1.0 Hz, 1H), 5.09 (s, 2H), 3.43 (s, 4H), 2.46 (s, 4H), 2.34 (s, 3H). ^{13}C-NMR (101 MHz, $CDCl_3$) δ 194.2, 165.6, 160.2, 143.4, 135.8, 135.6, 130.0, 129.8, 129.5, 128.9, 128.8, 128.7, 128.6, 128.6, 128.6, 128.5, 125.6, 123.4, 119.9, 111.7, 111.4, 102.1, 71.4, 54.1, 46.6, 45.9. HRMS (ESI-FT-ICR) m/z: 547.2708 $[M^+]$; calcd. for $C_{34}H_{35}N_4O_3$: 547.2709. (58% yield).

2. NMR and Mass Spectra:

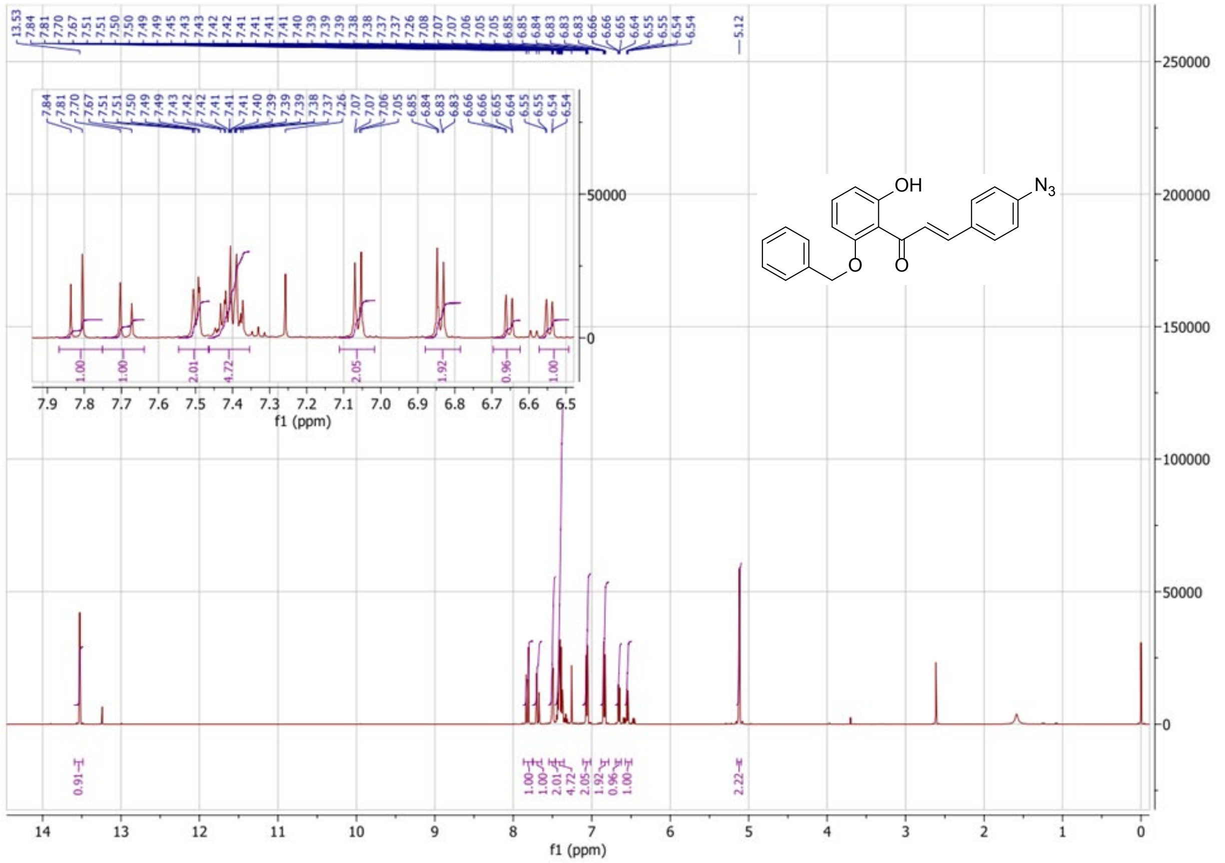

Figure S1: ¹H-NMR (500 MHz, $CDCl_3$) Spectrum of compound **3**

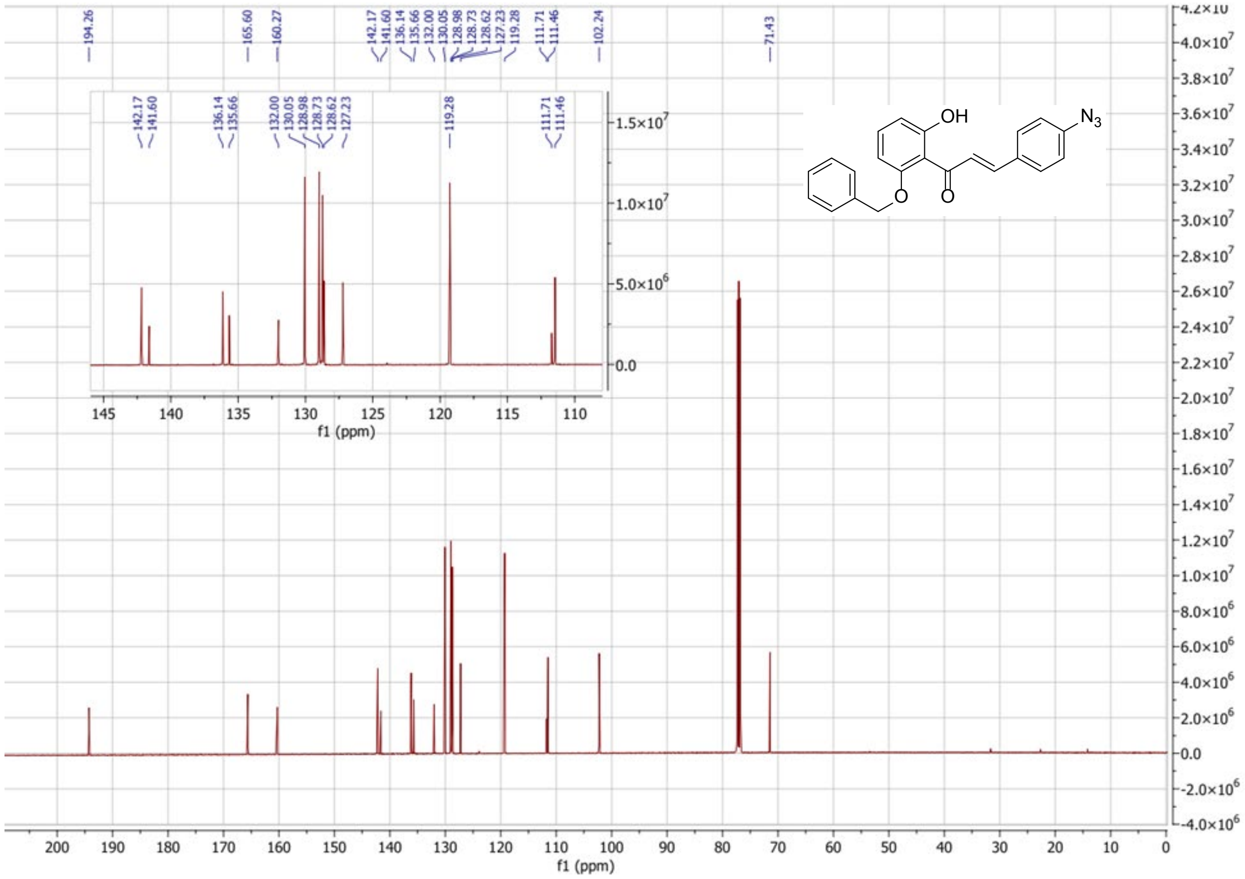

Figure S2: ¹³C-NMR (125 MHz, $CDCl_3$) Spectrum of compound **3**

Elemental Composition Report Page 1

Multiple Mass Analysis: 2 mass(es) processed
Tolerance = 5.0 PPM / DBE: min = -3.0, max = 120.0
Element prediction: Off
Number of isotope peaks used for i-FIT = 3

Monoisotopic Mass, Even Electron Ions
1095 formula(e) evaluated with 3 results within limits (all results (up to 1000) for each mass)
Elements Used:
C: 0-60 H: 0-60 N: 0-3 O: 0-16 Na: 0-1
Inaki
(ESI-19) (992) Inaki (IB-JP 22) 54 (2.331)

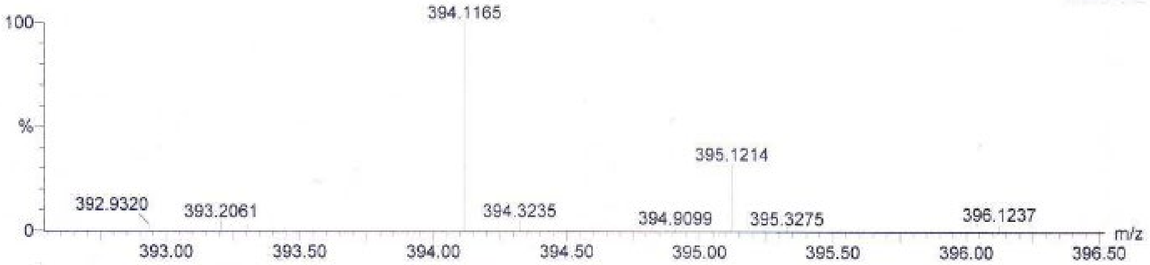

Minimum:	20.00				-3.0				
Maximum:	100.00		5.0	5.0	120.0				
Mass	RA	Calc. Mass	mDa	PPM	DBE	i-FIT	i-FIT (Norm)	Formula
394.1165	100.00	394.1168	-0.3	-0.8	15.5	32.6	0.0	C22 H17 N3 O3 Na
		394.1173	-0.8	-2.0	-2.5	37.6	5.0	C9 H25 N O14 Na
395.1214	32.01	395.1219	-0.5	-1.3	10.5	24.1	0.0	C19 H20 N2 O6 Na

Figure S3: HRESI-MS Spectrum of compound **3**

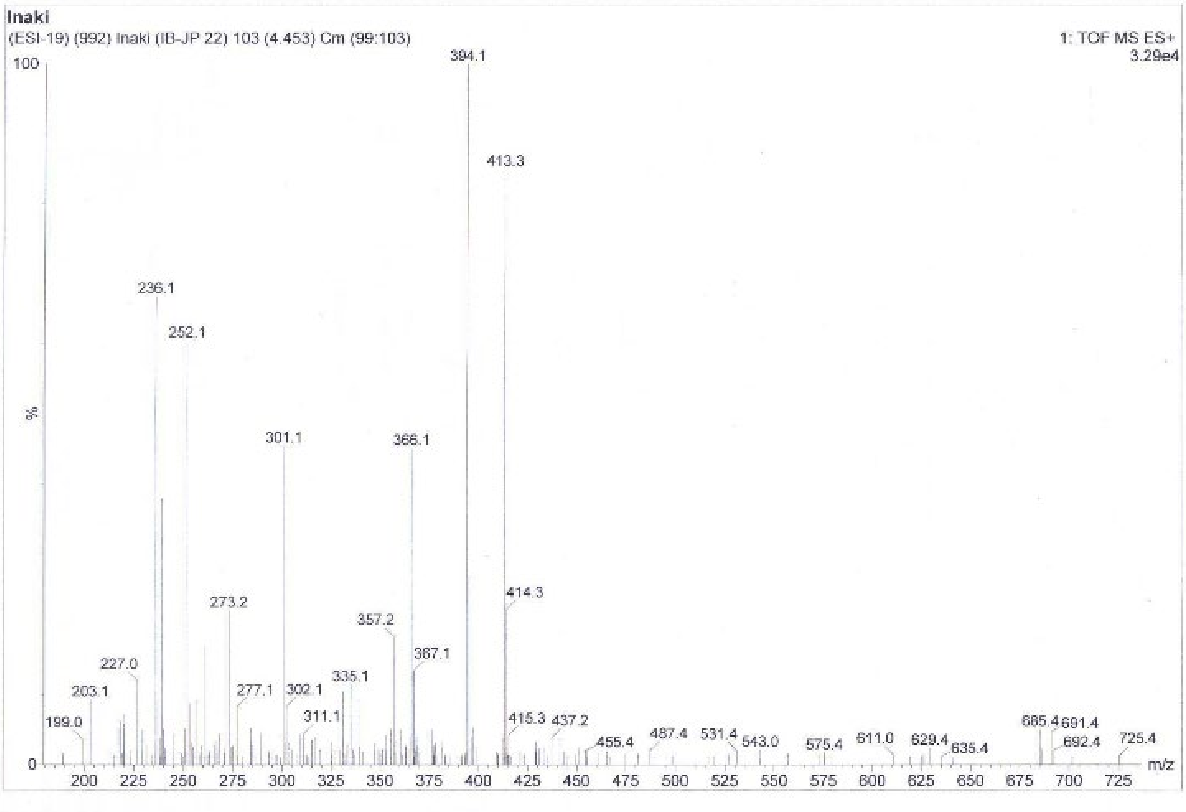

Figure S4: ESI-MS Spectrum of compound **3**

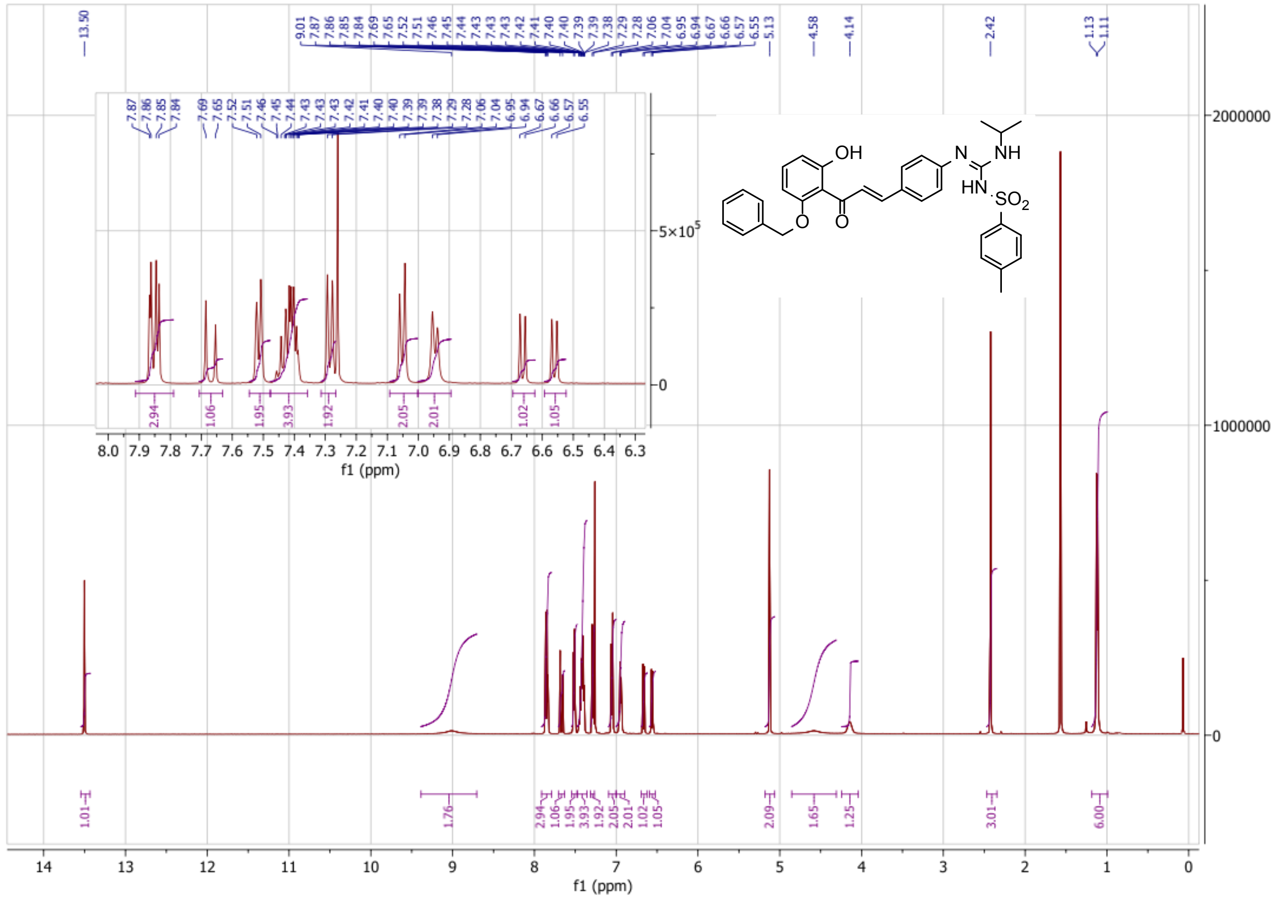

Figure S5: ^{1}H-NMR (500 MHz, $CDCl_3$) Spectrum of compound **6a**

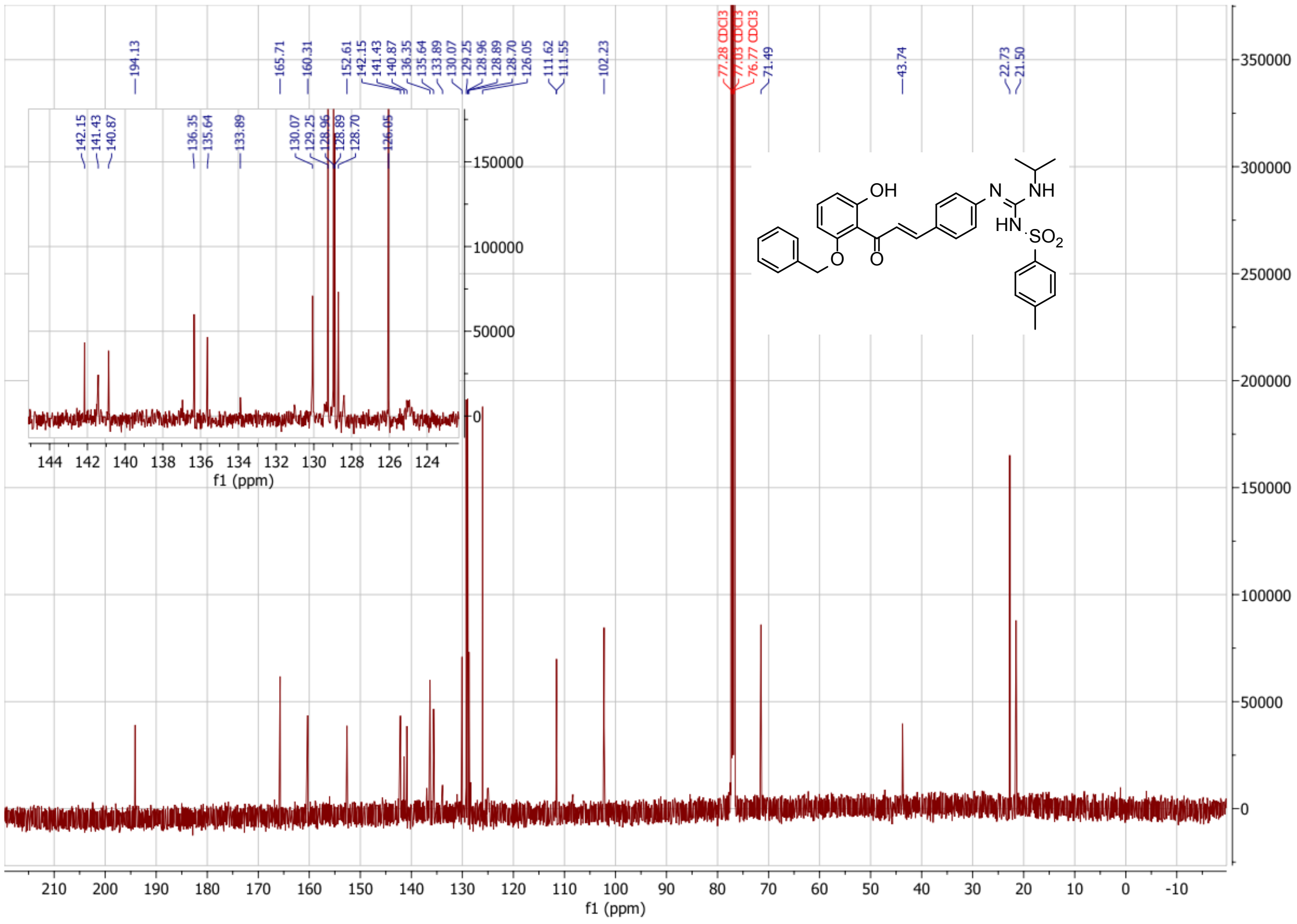

Figure S6: ^{13}C-NMR (125 MHz, $CDCl_3$) Spectrum of compound **6a**

Elemental Composition Report

Tolerance = 5.0 PPM / DBE: min = -10.0, max = 1000.0
Element prediction: Off
Number of isotope peaks used for i-FIT = 2

Monoisotopic Mass, Even Electron Ions
2930 formula(e) evaluated with 12 results within limits (all results (up to 1000) for each mass)
Elements Used:
C: 0-60 H: 0-100 N: 0-4 O: 0-12 Na: 0-1 S: 0-2

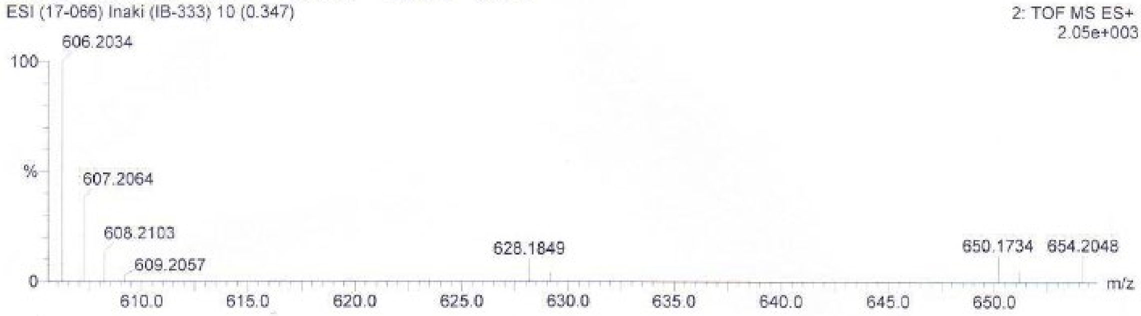

Minimum:	60.00				-10.0		
Maximum:	100.00		100.0	5.0	1000.0		
Mass	RA	Calc. Mass	mDa	PPM	DBE	i-FIT	Formula
606.2034	100.00	606.2038	-0.4	-0.7	25.5	13.3	C39 H32 N3 S2
		606.2029	0.5	0.8	26.5	6.9	C38 H28 N3 O5
		606.2039	-0.5	-0.8	18.5	0.5	C33 H33 N3 O5 Na S
		606.2043	-0.9	-1.5	7.5	7.4	C26 H40 N O11 S2
		606.2045	-1.1	-1.8	27.5	13.3	C41 H29 N O3 Na
		606.2019	1.5	2.5	4.5	14.4	C24 H41 N O11 Na S2
		606.2014	2.0	3.3	22.5	7.8	C37 H33 N3 Na S2
		606.2009	2.5	4.1	12.5	2.5	C29 H36 N O11 S
		606.2063	-2.9	-4.8	21.5	2.8	C35 H32 N3 O5 S
		606.2005	2.9	4.8	23.5	3.2	C36 H29 N3 O5 Na
		606.2004	3.0	4.9	30.5	21.2	C42 H28 N3 S
		606.2064	-3.0	-4.9	14.5	2.8	C29 H33 N3 O10 Na

Figure S7: HRESI-MS Spectrum of compound **6a**

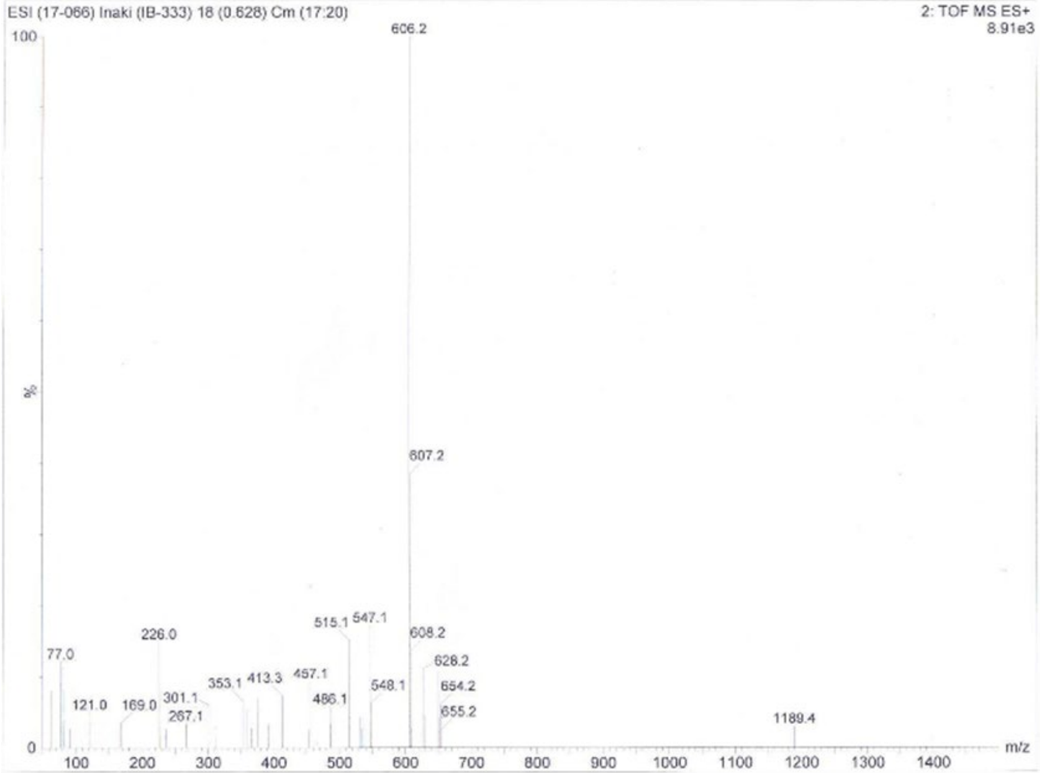

Figure S8: ESI-MS Spectrum of compound **6a**

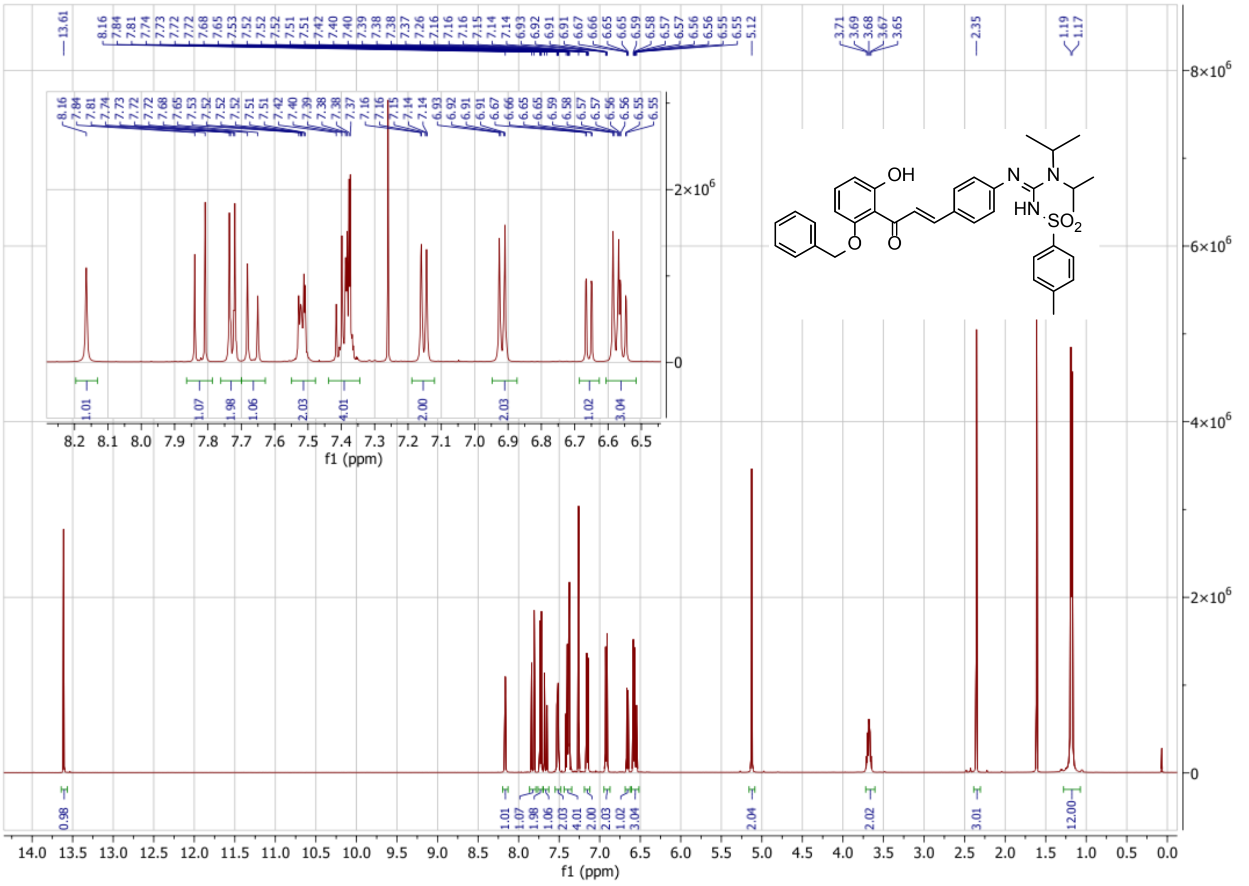

Figure S9: ^{1}H-NMR (500 MHz, $CDCl_3$) Spectrum of compound **6b**

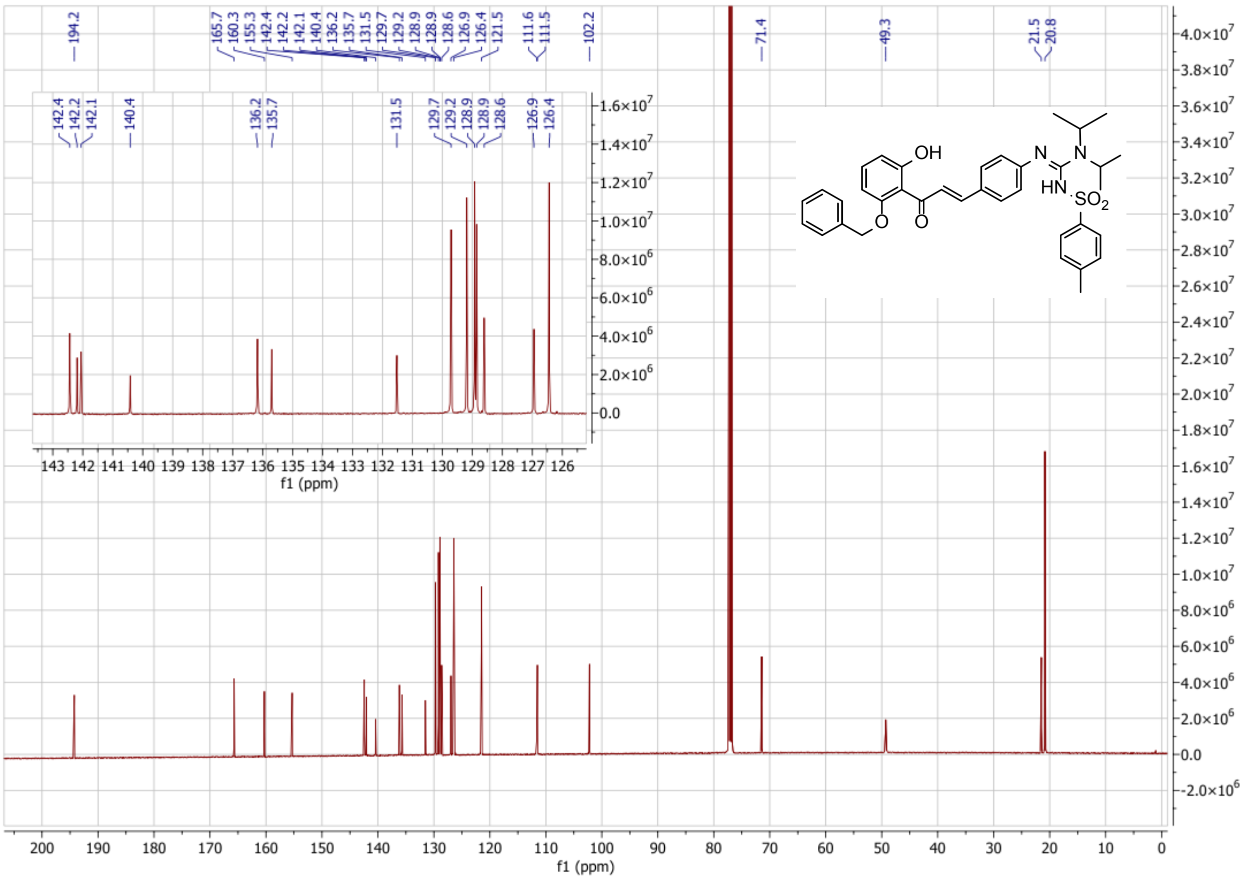

Figure S10: ^{13}C-NMR (125 MHz, $CDCl_3$) Spectrum of compound **6b**

Elemental Composition Report Page 1

Tolerance = 5.0 PPM / DBE: min = -10.0, max = 1000.0
Element prediction: Off
Number of isotope peaks used for i-FIT = 2

Monoisotopic Mass, Even Electron Ions
3090 formula(e) evaluated with 11 results within limits (all results (up to 1000) for each mass)
Elements Used:
C: 0-60 H: 0-100 N: 0-4 O: 0-12 Na: 0-1 S: 0-2

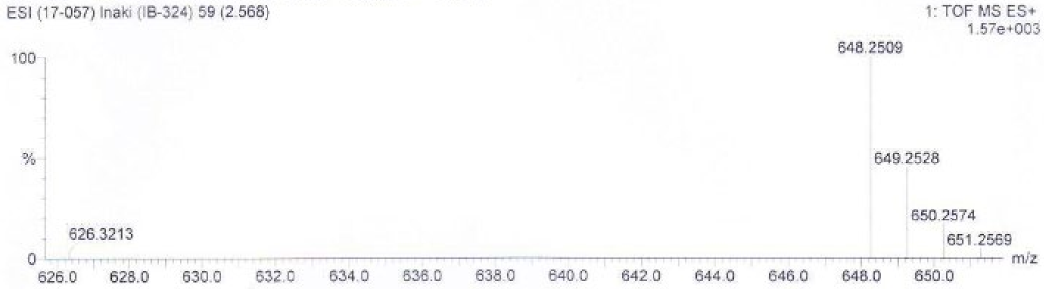

Minimum:	60.00				-10.0		
Maximum:	100.00		100.0	5.0	1000.0		

Mass	RA	Calc. Mass	mDa	PPM	DBE	i-FIT	Formula
648.2509	100.00	648.2508	0.1	0.2	18.5	0.7	C36 H39 N3 O5 Na S
		648.2507	0.2	0.3	25.5	2.8	C42 H38 N3 S2
		648.2512	-0.3	-0.5	7.5	12.8	C29 H46 N O11 S2
		648.2515	-0.6	-0.9	27.5	2.8	C44 H35 N O3 Na
		648.2498	1.1	1.7	26.5	0.7	C41 H34 N3 O5
		648.2488	2.1	3.2	4.5	19.8	C27 H47 N O11 Na S2
		648.2532	-2.3	-3.5	21.5	0.1	C38 H38 N3 O5 S
		648.2533	-2.4	-3.7	14.5	7.2	C32 H39 N3 O10 Na
		648.2483	2.6	4.0	22.5	1.0	C40 H39 N3 Na S2
		648.2479	3.0	4.6	12.5	7.0	C32 H42 N O11 S
		648.2539	-3.0	-4.6	30.5	5.8	C46 H34 N O3

Figure S11: HRESI-MS Spectrum of compound **6b**

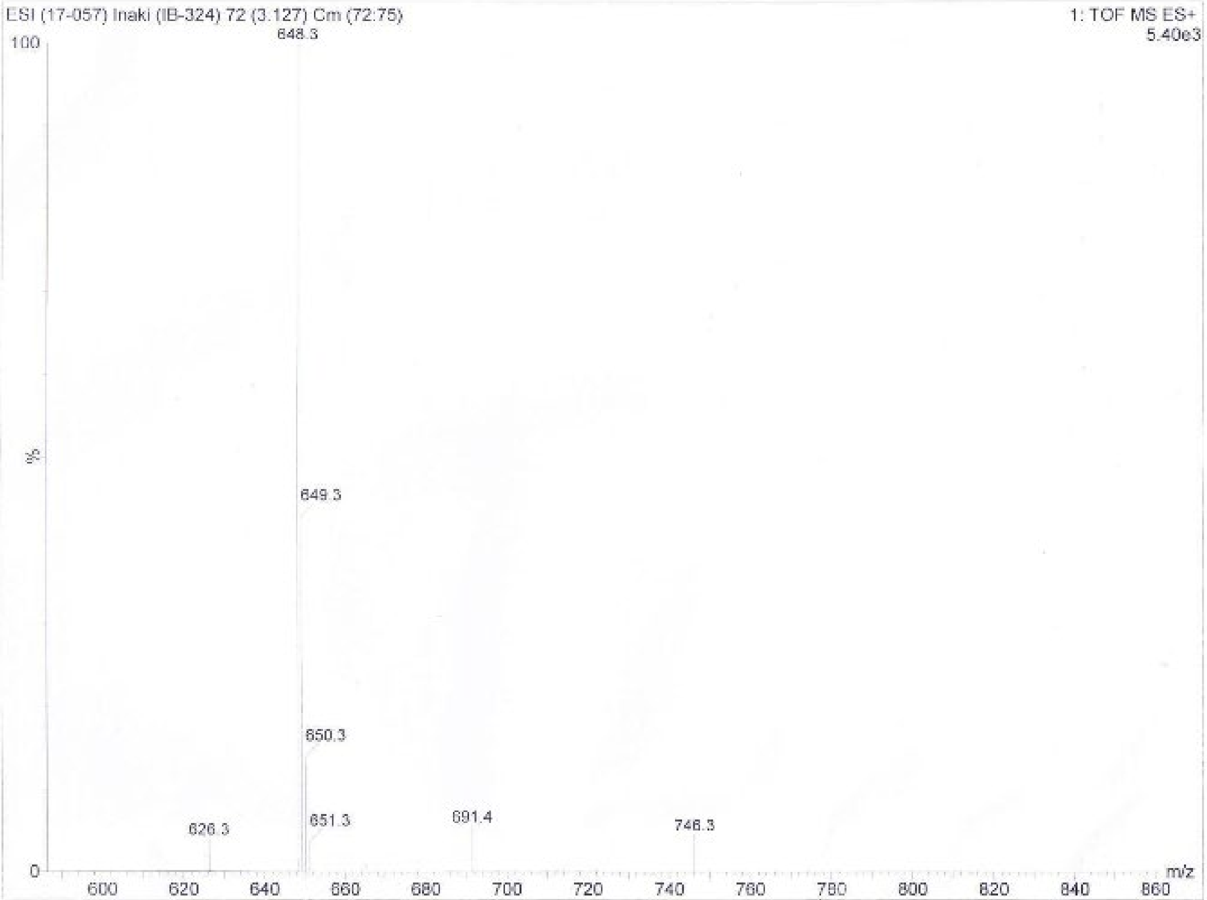

Figure S12: ESI-MS Spectrum of compound **6b**

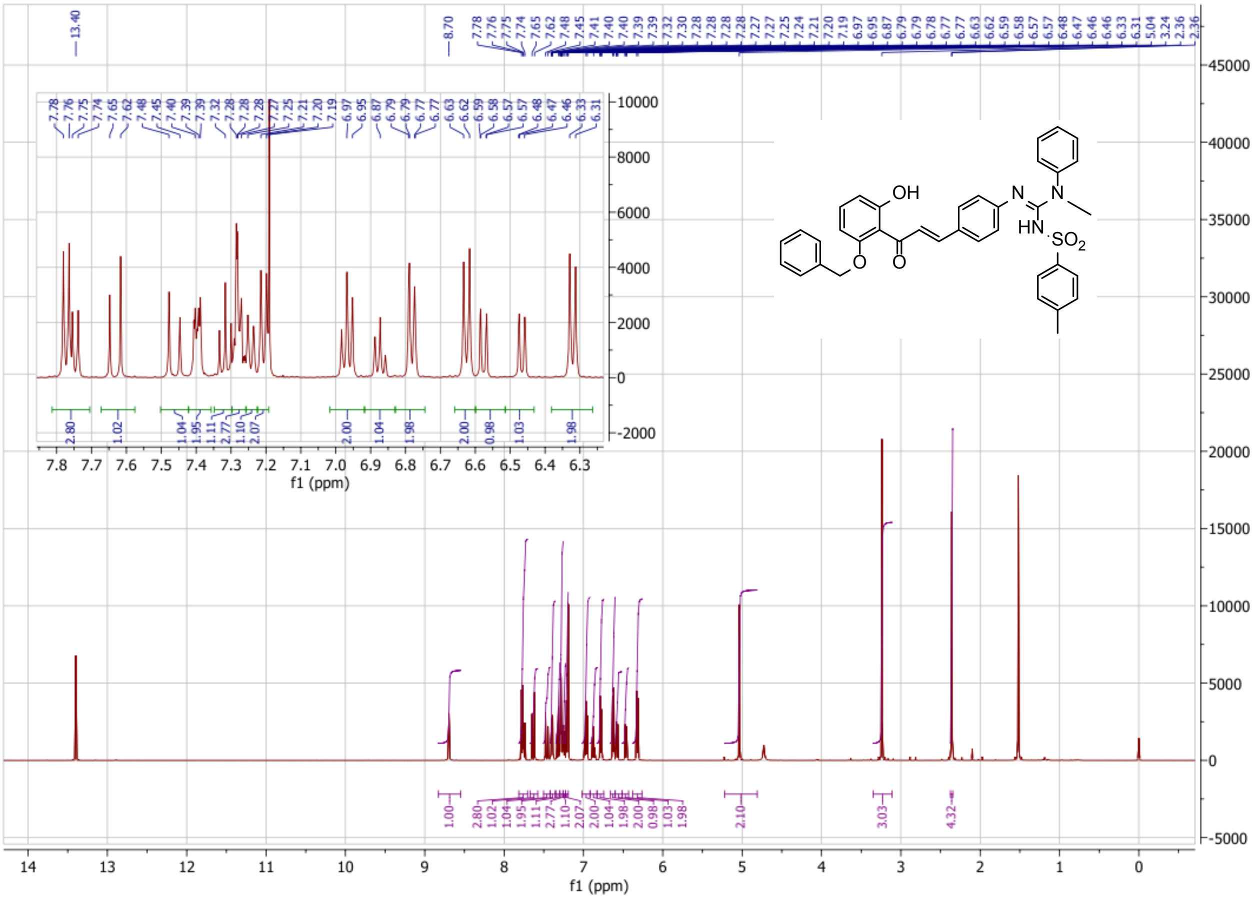

Figure S13: ^{1}H-NMR (500 MHz, CDCl3) Spectrum of compound **6c**

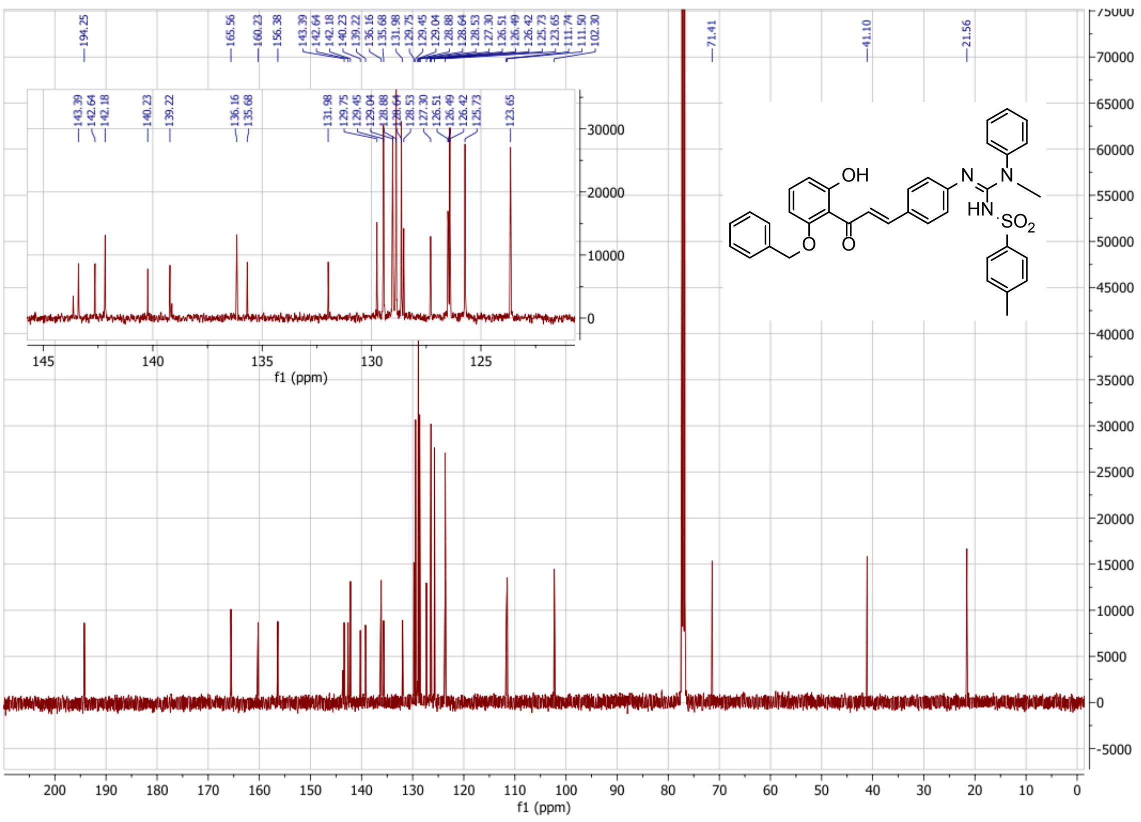

Figure S14: ^{13}C-NMR (125 MHz, CDCl3) Spectrum of compound **6c**

Elemental Composition Report **Page 1**

Tolerance = 5.0 PPM / DBE: min = -10.0, max = 1000.0
Element prediction: Off
Number of isotope peaks used for i-FIT = 2

Monoisotopic Mass, Even Electron Ions
3103 formula(e) evaluated with 12 results within limits (all results (up to 1000) for each mass)
Elements Used:
C: 0-60 H: 0-100 N: 0-4 O: 0-12 Na: 0-1 S: 0-2

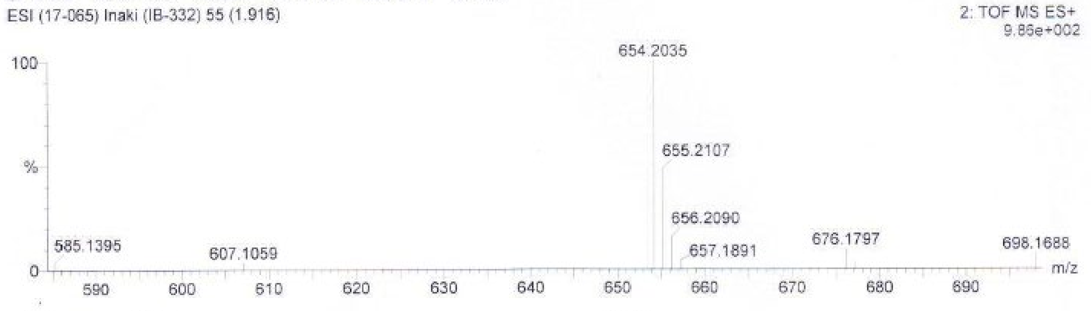

Minimum:	60.00				-10.0		
Maximum:	100.00		100.0	5.0	1000.0		
Mass	RA	Calc. Mass	mDa	PPM	DBE	i-FIT	Formula
654.2035	100.00	654.2038	-0.3	-0.5	29.5	0.7	C43 H32 N3 S2
		654.2039	-0.4	-0.6	22.5	1.3	C37 H33 N3 O5 Na S
		654.2029	0.6	0.9	30.5	0.1	C42 H28 N3 O5
		654.2043	-0.8	-1.2	11.5	10.8	C30 H40 N O11 S2
		654.2045	-1.0	-1.5	31.5	0.7	C45 H29 N O3 Na
		654.2019	1.6	2.4	8.5	15.9	C28 H41 N O11 Na S2
		654.2014	2.1	3.2	26.5	0.2	C41 H33 N3 Na S2
		654.2009	2.6	4.0	16.5	6.6	C33 H36 N O11 S
		654.2063	-2.8	-4.3	25.5	0.3	C39 H32 N3 O5 S
		654.2064	-2.9	-4.4	18.5	6.7	C33 H33 N3 O10 Na
		654.2005	3.0	4.6	27.5	0.4	C40 H29 N3 O5 Na
		654.2004	3.1	4.7	34.5	2.2	C46 H28 N3 S

Figure S15: HRESI-MS Spectrum of compound **6c**

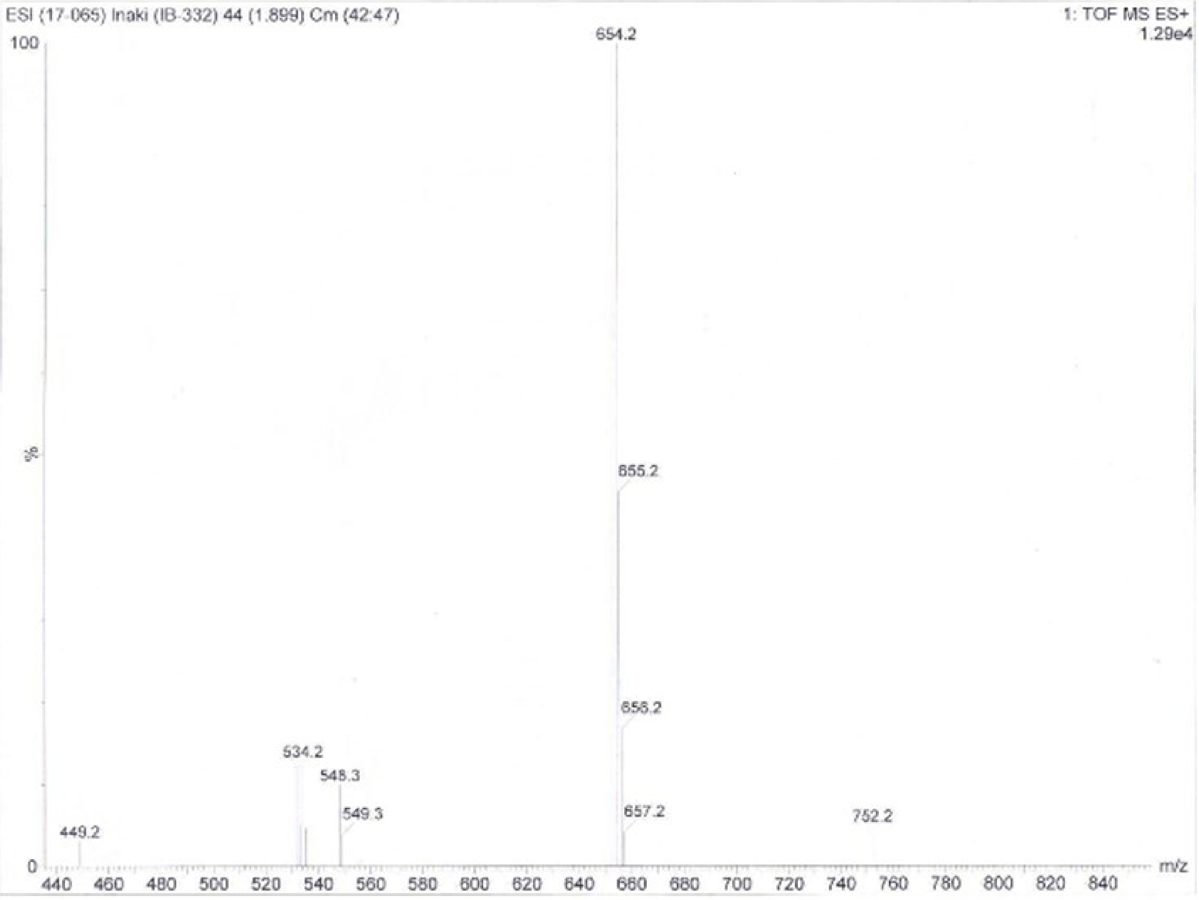

Figure S16: ESI-MS Spectrum of compound **6c**

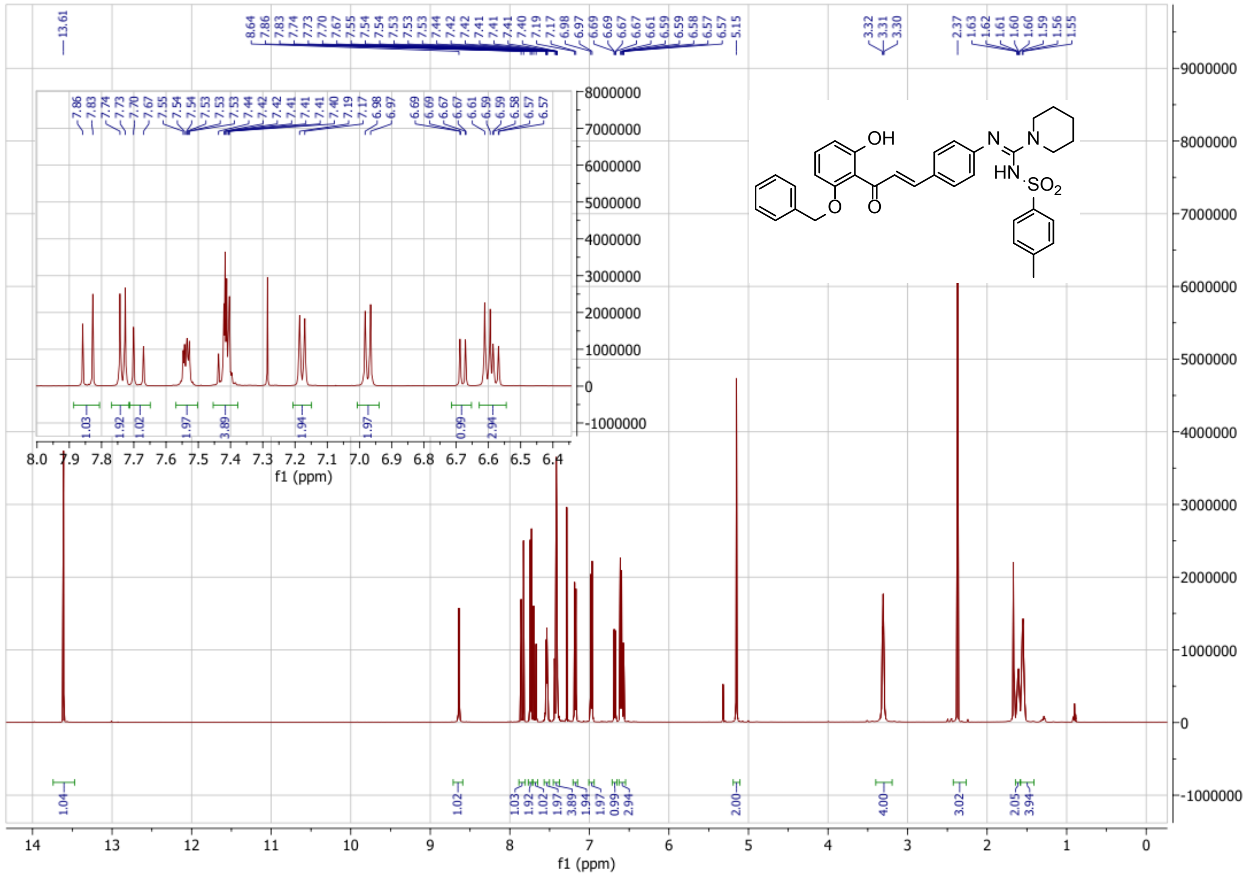

Figure S17: ¹H-NMR (500 MHz, CDCl₃) Spectrum of compound **6d**

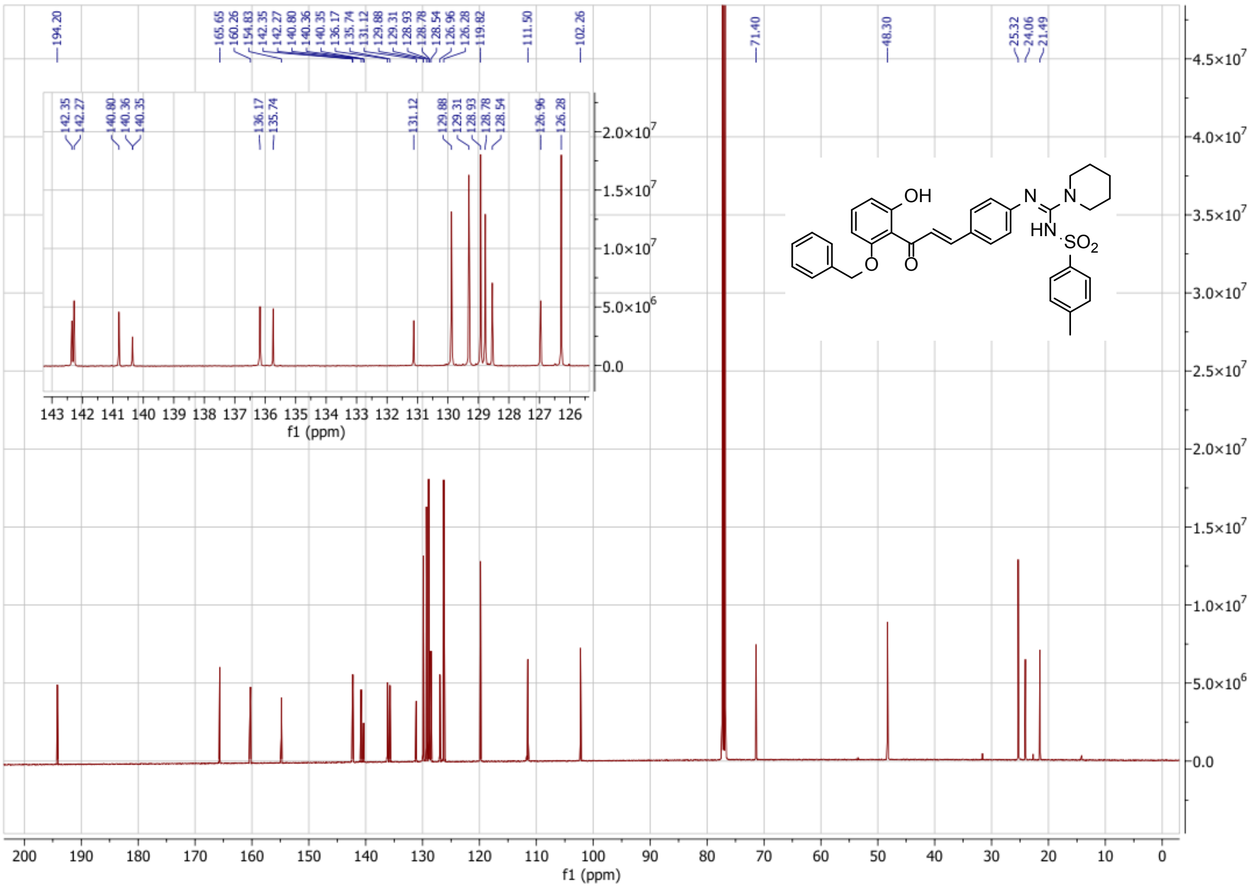

Figure S18: ¹³C-NMR (125 MHz, CDCl₃) Spectrum of compound **6d**

Elemental Composition Report

Tolerance = 5.0 PPM / DBE: min = -10.0, max = 1000.0
Element prediction: Off
Number of isotope peaks used for i-FIT = 2

Monoisotopic Mass, Even Electron Ions
3037 formula(e) evaluated with 11 results within limits (all results (up to 1000) for each mass)
Elements Used:
C: 0-60 H: 0-100 N: 0-4 O: 0-12 Na: 0-1 S: 0-2
ESI (17-058) Inaki (IB-325) 5 (0.172)

2: TOF MS ES+
1.35e+003

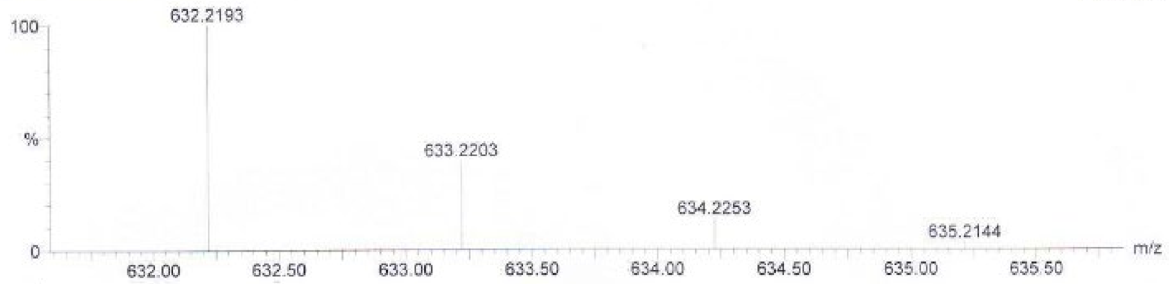

Minimum:	60.00				-10.0		
Maximum:	100.00		100.0	5.0	1000.0		
Mass	RA	Calc. Mass	mDa	PPM	DBE	i-FIT	Formula
632.2193	100.00	632.2194	-0.1	-0.2	26.5	9.3	C41 H34 N3 S2
		632.2195	-0.2	-0.3	19.5	0.5	C35 H35 N3 O5 Na S
		632.2199	-0.6	-0.9	8.5	3.8	C28 H42 N O11 S2
		632.2185	0.8	1.3	27.5	5.1	C40 H30 N3 O5
		632.2202	-0.9	-1.4	28.5	9.3	C43 H31 N O3 Na
		632.2175	1.8	2.8	5.5	7.8	C26 H43 N O11 Na S2
		632.2170	2.3	3.6	23.5	5.6	C39 H35 N3 Na S2
		632.2219	-2.6	-4.1	22.5	2.3	C37 H34 N3 O5 S
		632.2220	-2.7	-4.3	15.5	1.3	C31 H35 N3 O10 Na
		632.2166	2.7	4.3	13.5	1.1	C31 H38 N O11 S
		632.2161	3.2	5.1	24.5	2.5	C38 H31 N3 O5 Na

Figure S19: HRESI-MS Spectrum of compound **6d**

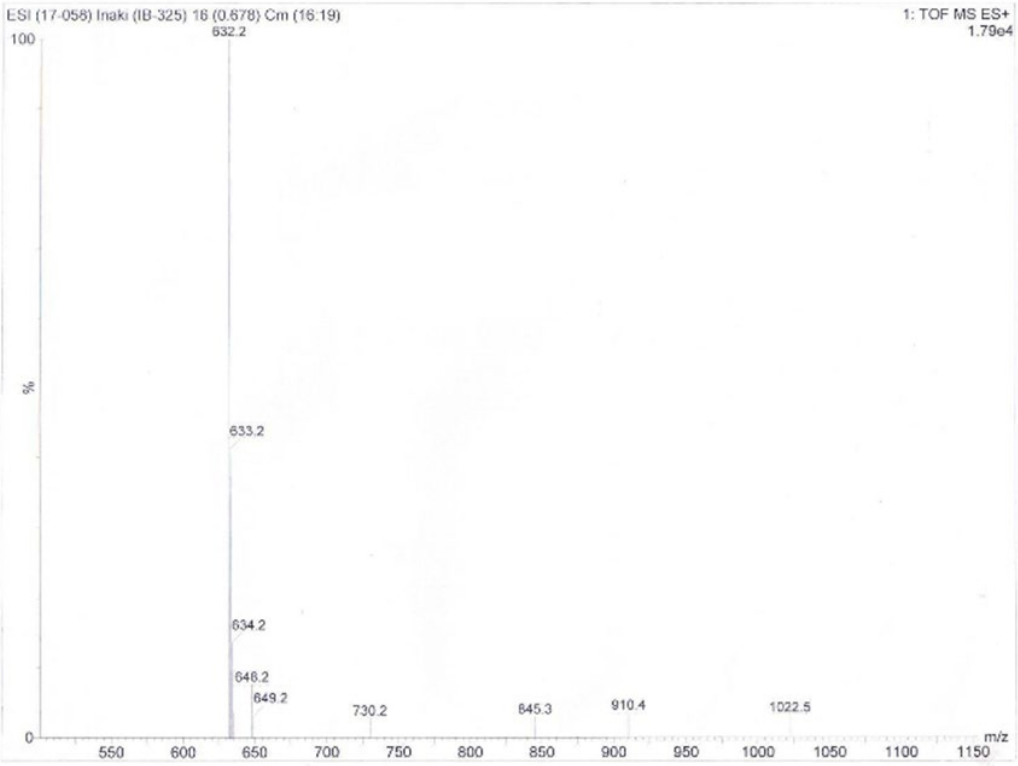

Figure S20: ESI-MS Spectrum of compound **6d**

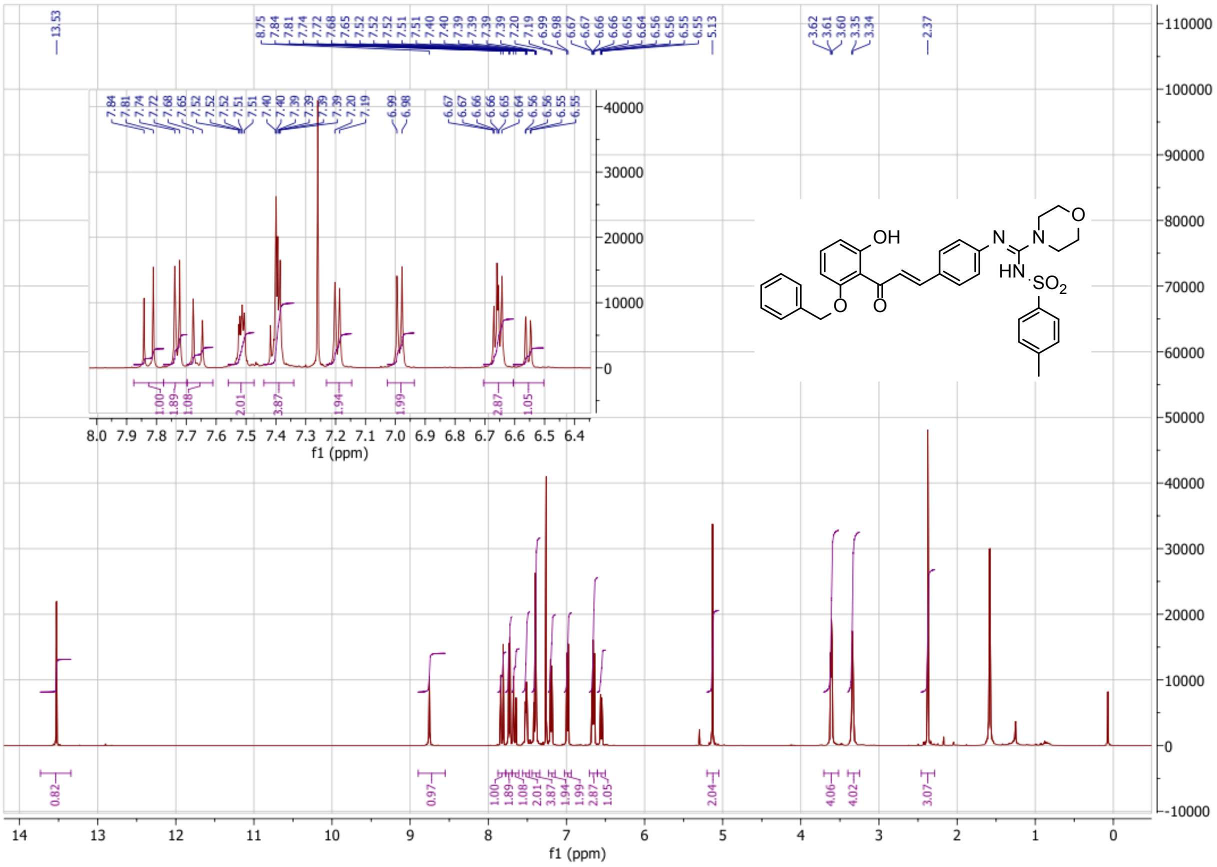

Figure S21: ¹H-NMR (500 MHz, $CDCl_3$) Spectrum of compound **6e**

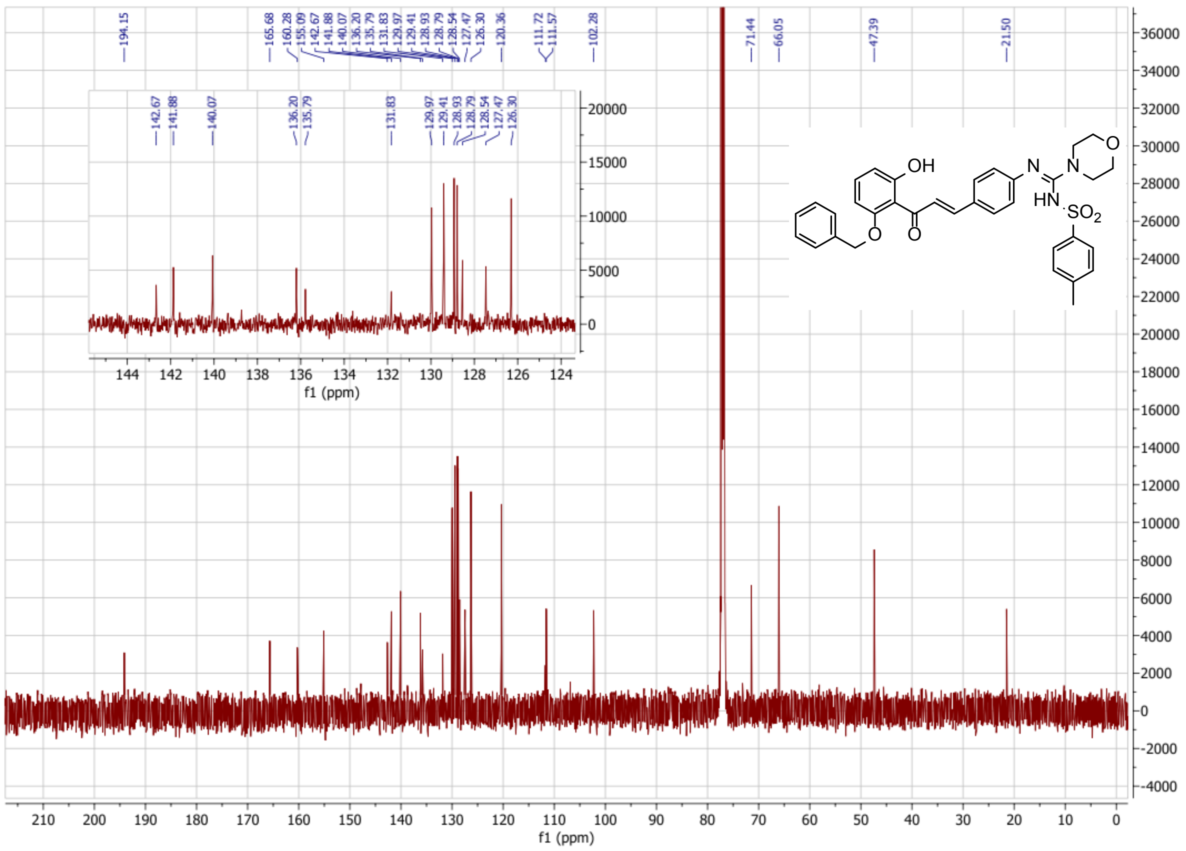

Figure S22: ¹³C-NMR (125 MHz, $CDCl_3$) Spectrum of compound **6e**

Elemental Composition Report

Page 1

Tolerance = 5.0 PPM / DBE: min = -10.0, max = 1000.0
Element prediction: Off
Number of isotope peaks used for i-FIT = 2

Monoisotopic Mass, Even Electron Ions
3043 formula(e) evaluated with 11 results within limits (all results (up to 1000) for each mass)
Elements Used:
C: 0-60 H: 0-100 N: 0-4 O: 0-12 Na: 0-1 S: 0-2

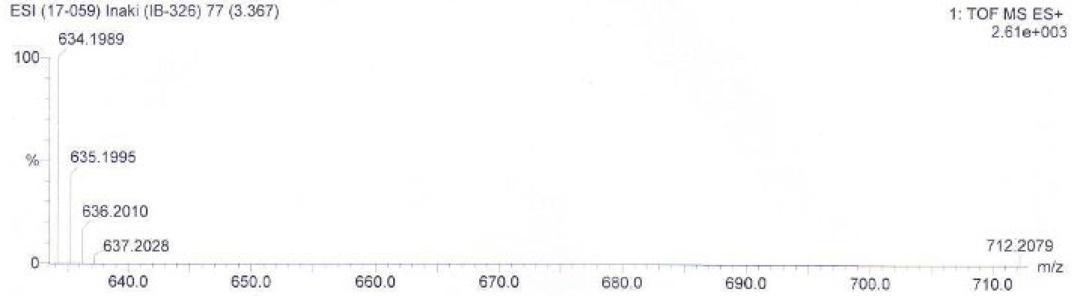

Mass	RA	Calc. Mass	mDa	PPM	DBE	i-FIT	Formula
Minimum:	60.00				-10.0		
Maximum:	100.00		100.0	5.0	1000.0		
634.1989	100.00	634.1988	0.1	0.2	19.5	1.3	C34 H33 N3 O6 Na S
		634.1987	0.2	0.3	26.5	4.7	C40 H32 N3 O S2
		634.1992	-0.3	-0.5	8.5	23.3	C27 H40 N O12 S2
		634.1994	-0.5	-0.8	28.5	4.8	C42 H29 N O4 Na
		634.1978	1.1	1.7	27.5	1.1	C39 H28 N3 O6
		634.1968	2.1	3.3	5.5	36.0	C25 H41 N O12 Na S2
		634.2012	-2.3	-3.6	22.5	0.3	C36 H32 N3 O6 S
		634.2013	-2.4	-3.8	15.5	13.4	C30 H33 N3 O11 Na
		634.1963	2.6	4.1	23.5	1.6	C38 H33 N3 O Na S2
		634.2018	-2.9	-4.6	31.5	10.0	C44 H28 N O4
		634.1958	3.1	4.9	13.5	12.7	C30 H36 N O12 S

Figure S23: HRESI-MS Spectrum of compound **6e**

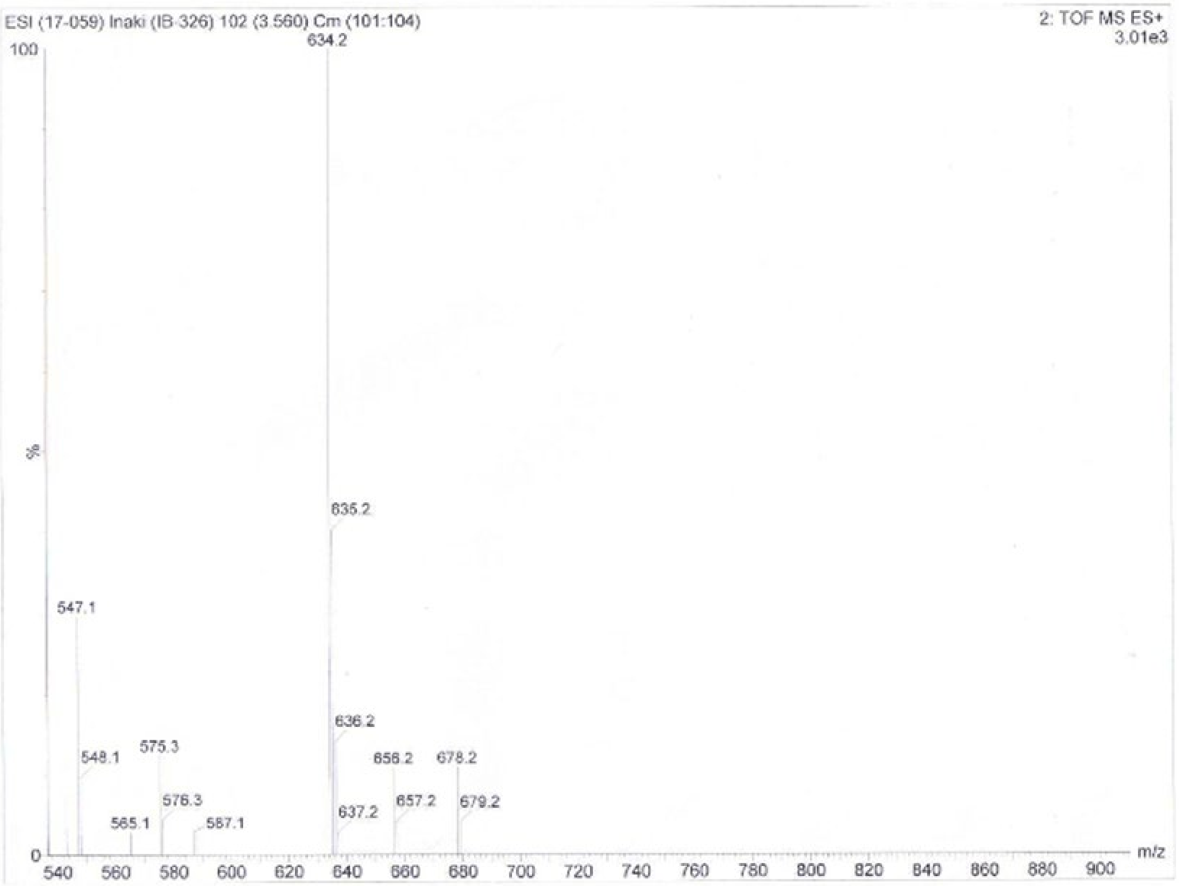

Figure S24: ESI-MS Spectrum of compound **6e**

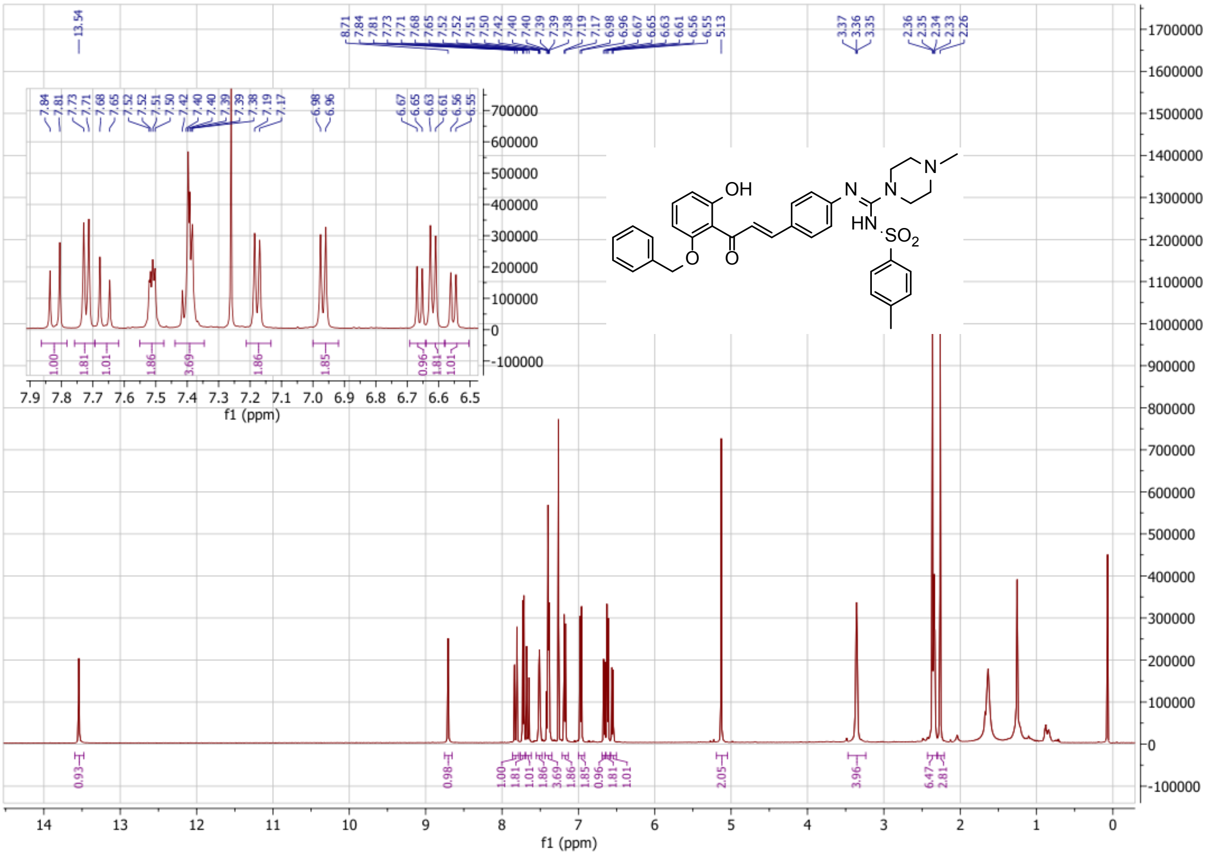

Figure S25: ^{1}H-NMR (500 MHz, $CDCl_3$) Spectrum of compound **6f**

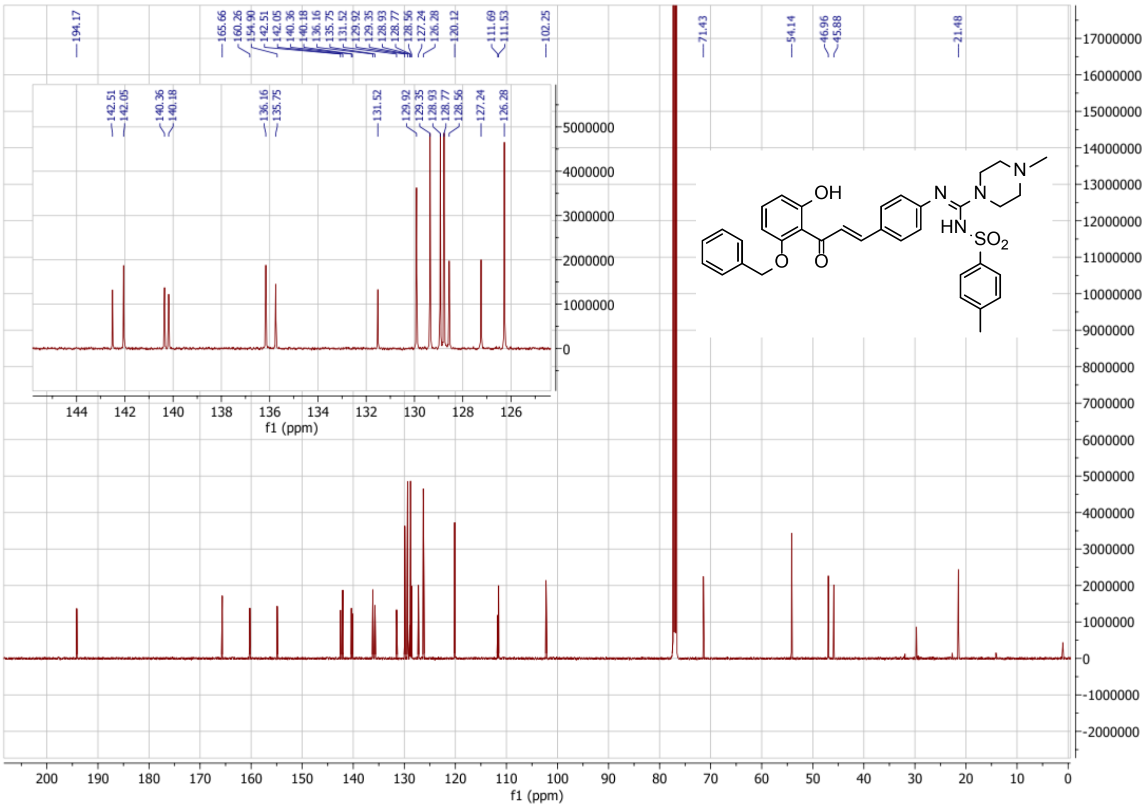

Figure S26: ^{13}C-NMR (125 MHz, $CDCl_3$) Spectrum of compound **6f**

Elemental Composition Report

Page 1

Multiple Mass Analysis: 2 mass(es) processed

Tolerance = 5.0 PPM / DBE: min = -10.0, max = 1000.0
Element prediction: Off
Number of isotope peaks used for i-FIT = 2

Monoisotopic Mass, Even Electron Ions
6921 formula(e) evaluated with 34 results within limits (all results (up to 1000) for each mass)
Elements Used:
C: 0-70 H: 0-90 N: 0-5 O: 0-20 Na: 0-1 S: 0-1

ESI (16-435) Inaki (IB JP-44 F5) 8 (0.330)

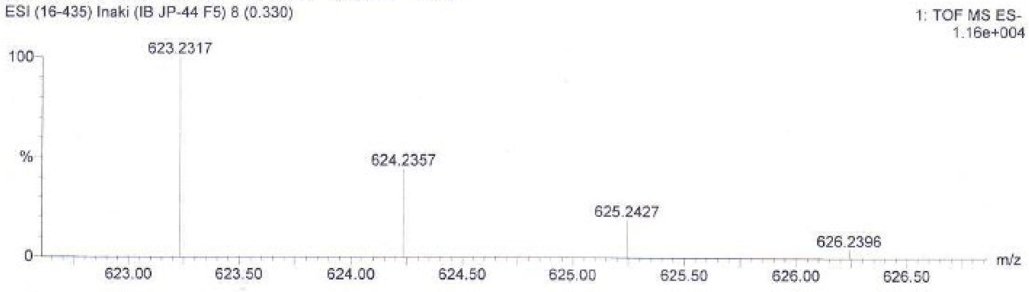

Minimum:	40.00				-10.0		
Maximum:	100.00		100.0	5.0	1000.0		
Mass	RA	Calc. Mass	mDa	PPM	DBE	i-FIT	Formula
623.2317	100.00	623.2316	0.1	0.2	8.5	148.2	C28 H40 O14 Na
		623.2315	0.2	0.3	15.5	20.1	C34 H39 O9 S
		623.2311	0.6	1.0	26.5	7.1	C41 H32 N2 O3 Na
		623.2309	0.8	1.3	-0.5	418.0	C20 H44 N2 O16 Na S
		623.2328	-1.1	-1.8	20.5	4.6	C35 H35 N4 O5 S
		623.2329	-1.2	-1.9	13.5	92.2	C29 H36 N4 O10 Na
		623.2304	1.3	2.1	17.5	19.0	C33 H36 N4 O5 Na S
		623.2333	-1.6	-2.6	2.5	319.5	C22 H43 N2 O16 S
		623.2300	1.7	2.7	7.5	226.3	C25 H39 N2 O16
		623.2335	-1.8	-2.9	29.5	22.0	C43 H31 N2 O3
		623.2340	-2.3	-3.7	11.5	95.9	C30 H39 O14
		623.2294	2.3	3.7	25.5	0.9	C38 H31 N4 O5
		623.2293	2.4	3.9	-1.5	550.9	C17 H43 N4 O18 S
		623.2291	2.6	4.2	12.5	46.6	C32 H40 O9 Na S
		623.2344	-2.7	-4.3	21.5	0.8	C38 H36 N2 O3 Na S
624.2357	44.09	624.2356	0.1	0.2	12.5	15.1	C30 H39 N3 O8 Na S
		624.2361	-0.4	-0.6	-5.5	227.1	C17 H47 N O19 Na S
		624.2361	-0.4	-0.6	28.5	29.8	C44 H34 N O S
		624.2362	-0.5	-0.8	21.5	1.2	C38 H35 N O6 Na
		624.2351	0.6	1.0	2.5	128.2	C22 H42 N O19
		624.2364	-0.7	-1.1	7.5	89.9	C23 H38 N5 O15
		624.2346	1.1	1.8	20.5	0.8	C35 H34 N3 O8

Figure S27: HRESI-MS Spectrum of compound **6f**

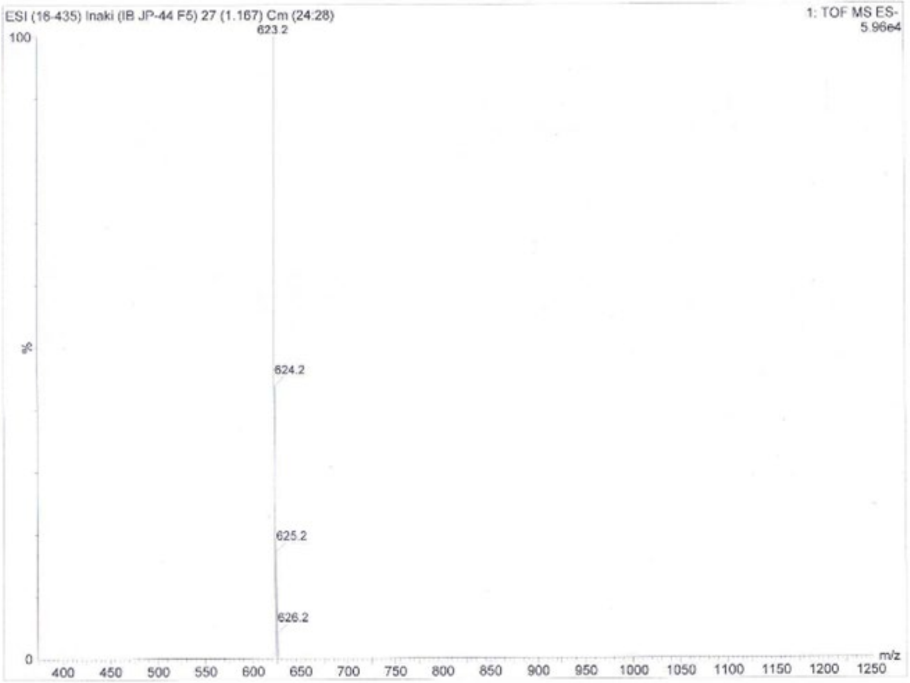

Figure S28: ESI-MS Spectrum of compound **6f**

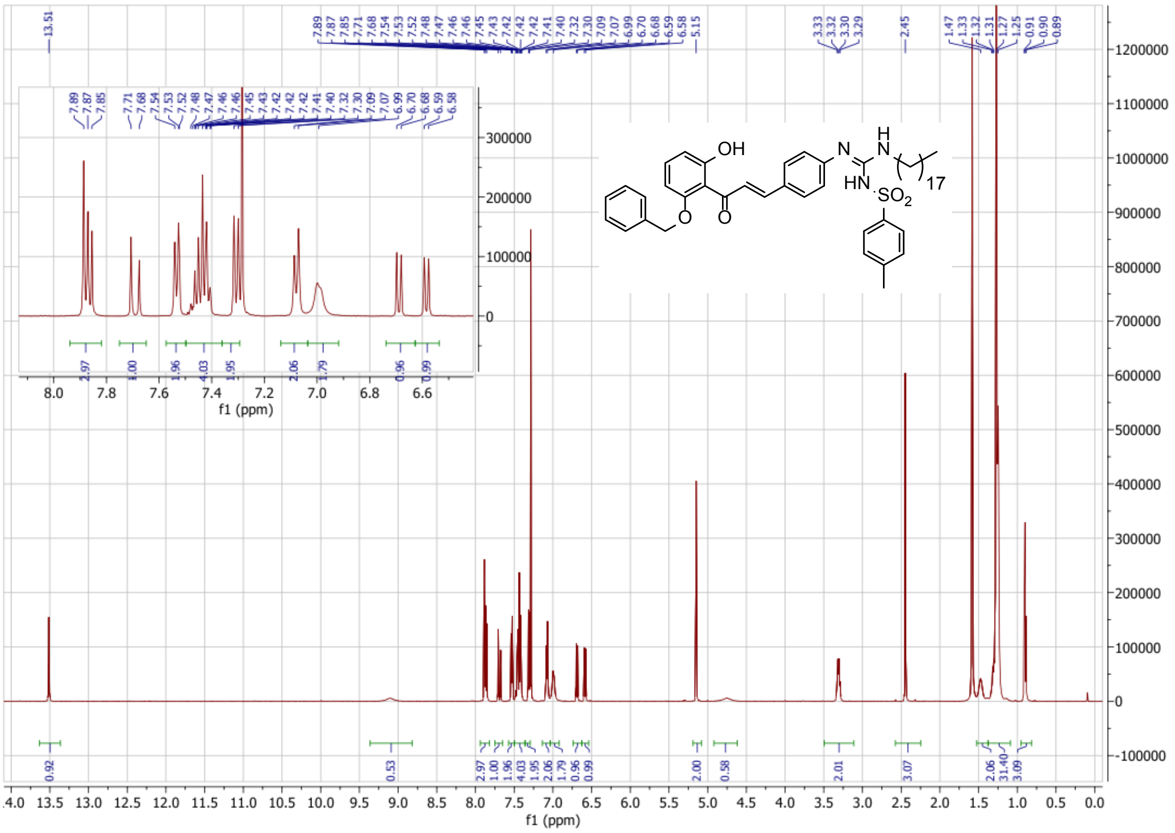

Figure S29: ^{1}H-NMR (500 MHz, $CDCl_3$) Spectrum of compound **6g**

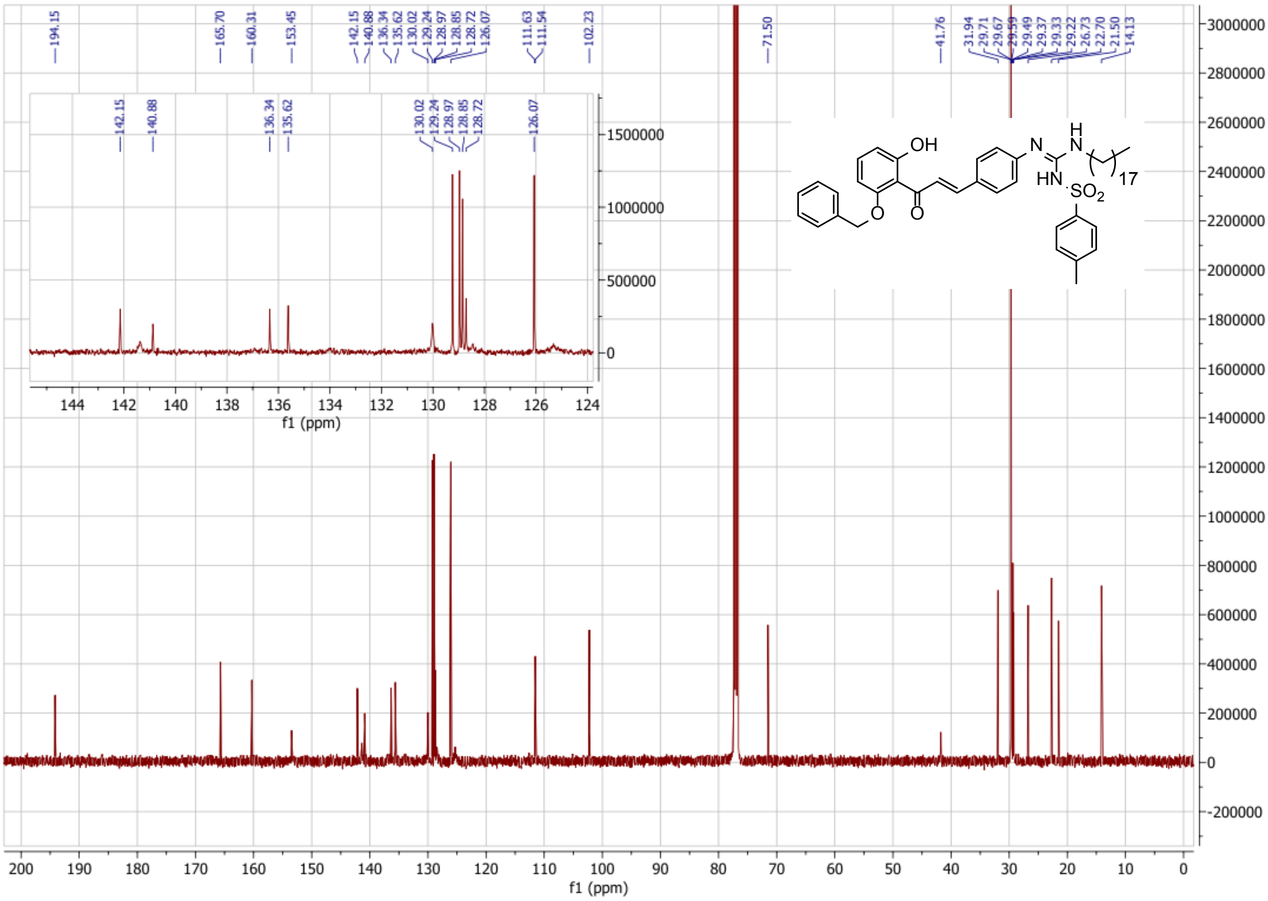

Figure S30: ^{13}C -NMR (125 MHz, $CDCl_3$) Spectrum of compound **6g**

Elemental Composition Report

Page 1

Tolerance = 5.0 PPM / DBE: min = -10.0, max = 1000.0
Element prediction: Off
Number of isotope peaks used for i-FIT = 2

Monoisotopic Mass, Even Electron Ions
3182 formula(e) evaluated with 14 results within limits (all results (up to 1000) for each mass)
Elements Used:
C: 0-60 H: 0-100 N: 0-4 O: 0-12 Na: 0-1 S: 0-2

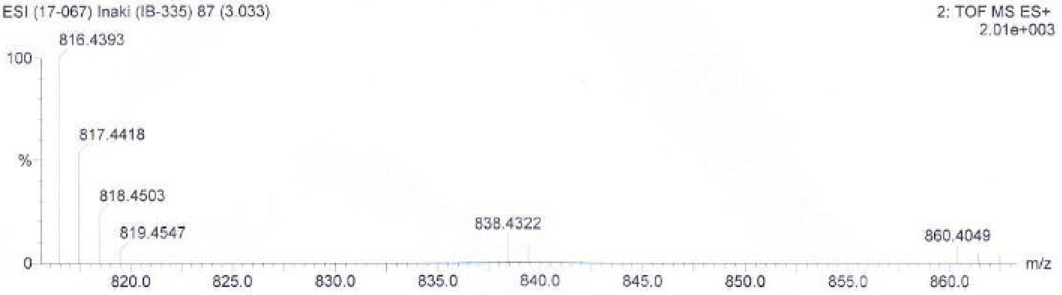

Mass	RA	Calc. Mass	mDa	PPM	DBE	i-FIT	Formula
Minimum:	60.00				-10.0		
Maximum:	100.00		100.0	5.0	1000.0		
816.4393	100.00	816.4393	0.0	0.0	27.5	10.2	C56 H59 N O3 Na
		816.4390	0.3	0.4	7.5	3.0	C41 H70 N O11 S2
		816.4386	0.7	0.9	18.5	0.8	C48 H63 N3 O5 Na S *
		816.4385	0.8	1.0	25.5	10.2	C54 H62 N3 S2
		816.4410	-1.7	-2.1	21.5	2.6	C50 H62 N3 O5 S
		816.4376	1.7	2.1	26.5	5.8	C53 H58 N3 O5
		816.4411	-1.8	-2.2	14.5	0.8	C44 H63 N3 O10 Na
		816.4417	-2.4	-2.9	30.5	14.9	C58 H58 N O3
		816.4366	2.7	3.3	4.5	6.7	C39 H71 N O11 Na S2
		816.4420	-2.7	-3.3	13.5	0.1	C45 H67 N3 O5 Na S2
		816.4361	3.2	3.9	22.5	6.4	C52 H63 N3 Na S2
		816.4426	-3.3	-4.0	22.5	6.0	C53 H63 N O3 Na S
		816.4357	3.6	4.4	12.5	0.8	C44 H66 N O11 S
		816.4352	4.1	5.0	23.5	3.1	C51 H59 N3 O5 Na

Figure S31: HRESI-MS Spectrum of compound **6g**

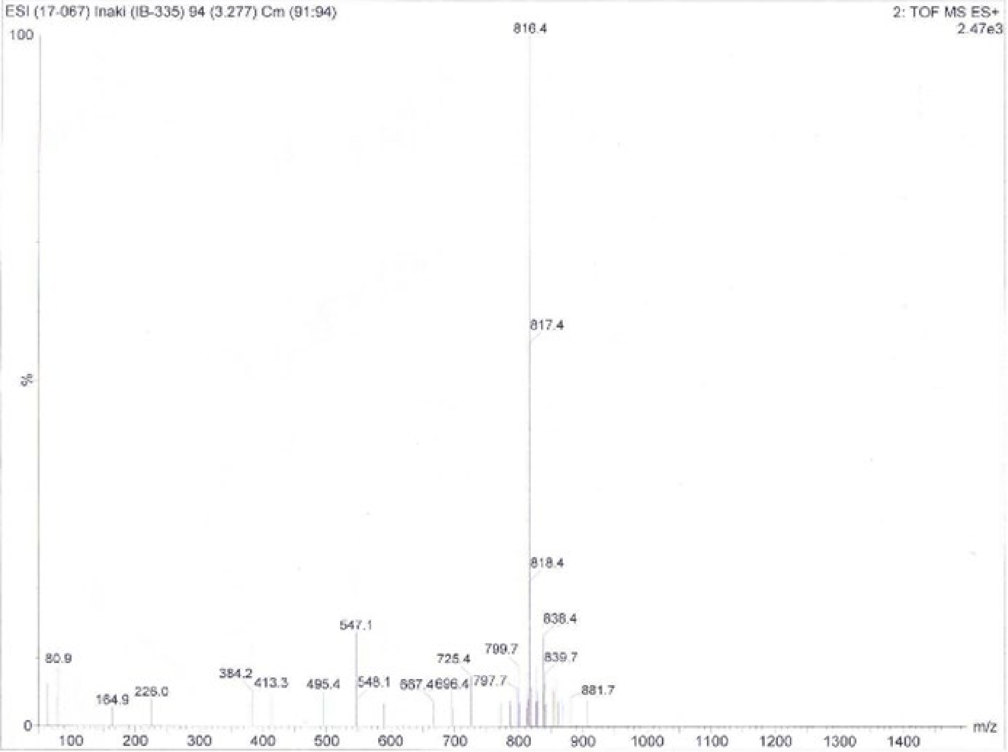

Figure S32: ESI-MS Spectrum of compound **6g**

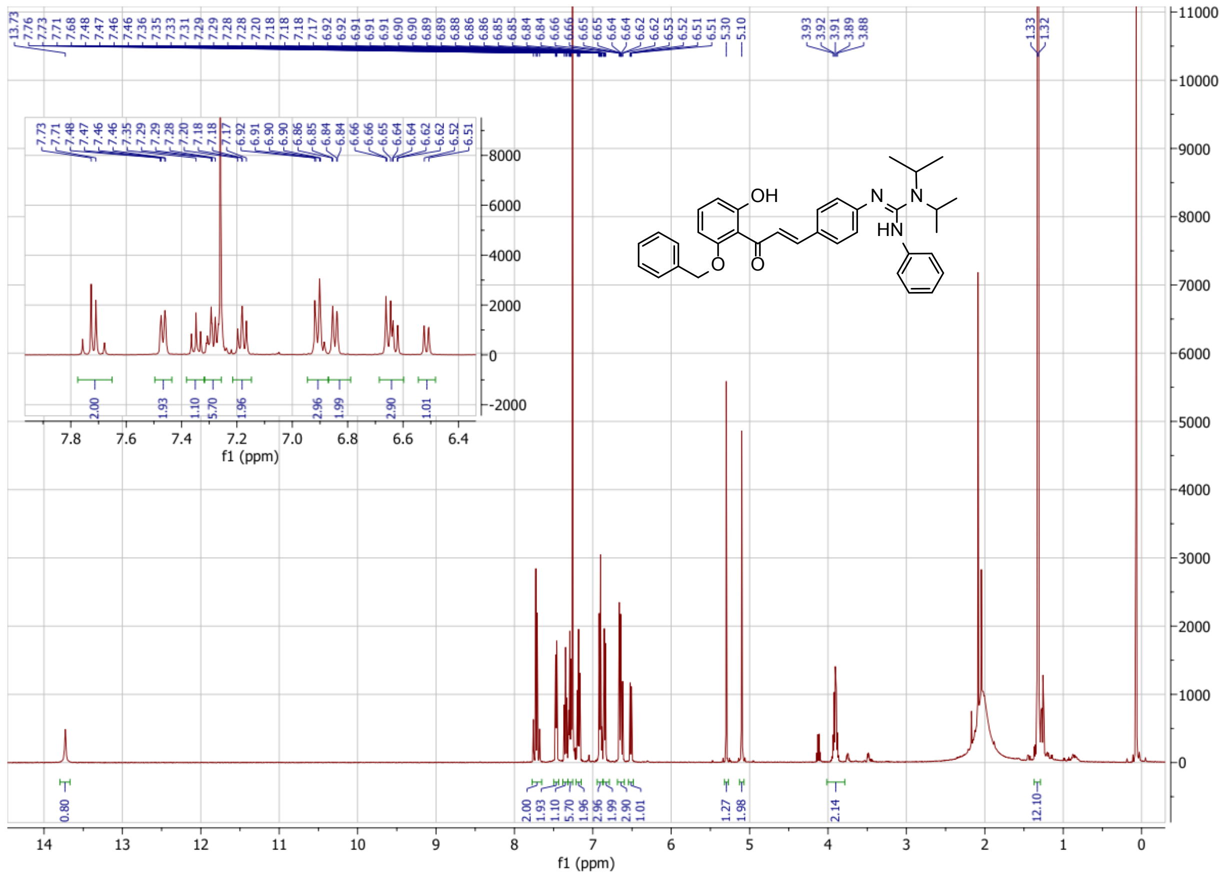

Figure S33: ^{1}H-NMR (500 MHz, $CDCl_3$) Spectrum of compound **6h**

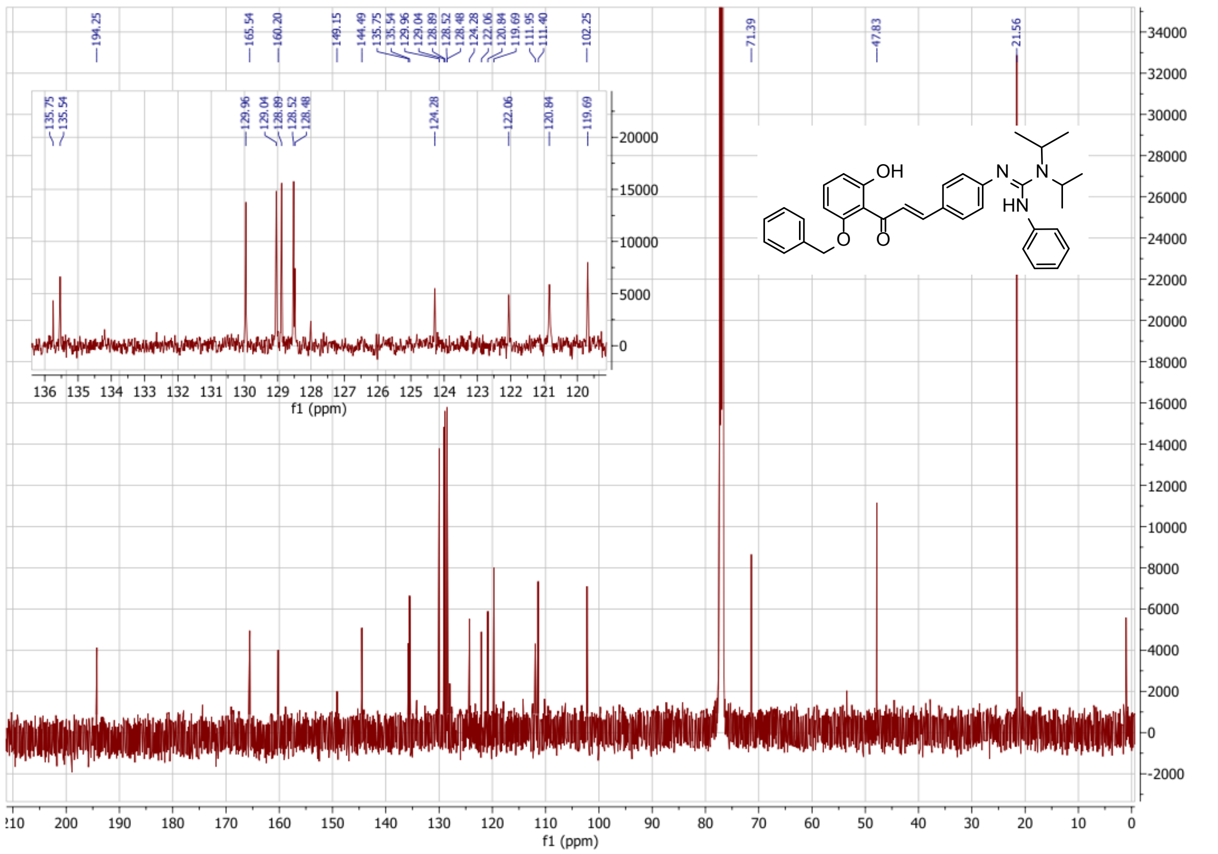

Figure S34: ^{13}C -NMR (125 MHz, $CDCl_3$) Spectrum of compound **6h**

Elemental Composition Report **Page 1**

Multiple Mass Analysis: 2 mass(es) processed
Tolerance = 5.0 PPM / DBE: min = -10.0, max = 1000.0
Element prediction: Off
Number of isotope peaks used for i-FIT = 2

Monoisotopic Mass, Even Electron Ions
5504 formula(e) evaluated with 21 results within limits (all results (up to 1000) for each mass)
Elements Used:
C: 0-60 H: 0-100 N: 0-4 O: 0-12 Na: 0-1 S: 0-2

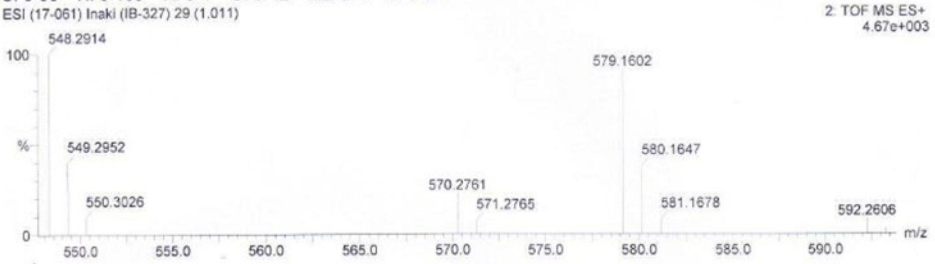

Minimum:	60.00				-10.0		
Maximum:	100.00		100.0	5.0	1000.0		

Mass	RA	Calc. Mass	mDa	PPM	DBE	i-FIT	Formula
548.2914	100.00	548.2913	0.1	0.2	18.5	0.2	C35 H38 N3 O3 •
		548.2923	-0.9	-1.6	10.5	7.2	C30 H43 N3 O3 Na S
		548.2903	1.1	2.0	-3.5	92.5	C21 H51 N O9 Na S2
		548.2927	-1.3	-2.4	-0.5	63.3	C23 H50 N O9 S2
		548.2929	-1.5	-2.7	19.5	4.2	C38 H39 N O Na
		548.2893	2.1	3.8	4.5	37.8	C26 H46 N O9 S
		548.2889	2.5	4.6	15.5	1.4	C33 H39 N3 O3 Na
		548.2887	2.7	4.9	-4.5	134.0	C18 H50 N3 O11 S2
579.1602	90.67	579.1600	0.2	0.3	13.5	21.2	C28 H32 N2 O6 Na S2
		579.1606	-0.4	-0.7	22.5	0.0	C36 H28 O4 Na S
		579.1596	0.6	1.0	30.5	7.4	C41 H23 O4
		579.1610	-0.8	-1.4	35.5	16.1	C42 H19 N4
		579.1591	1.1	1.9	14.5	39.9	C27 H28 N2 O11 Na
		579.1590	1.2	2.1	21.5	2.7	C33 H27 N2 O6 S
		579.1615	-1.3	-2.2	17.5	23.4	C29 H27 N2 O11
		579.1586	1.6	2.8	32.5	8.0	C40 H20 N4 Na
		579.1619	-1.7	-2.9	27.5	2.0	C37 H24 N4 Na S
		579.1583	1.9	3.3	12.5	40.0	C25 H31 N4 O8 S2
		579.1624	-2.2	-3.8	16.5	10.1	C30 H31 N2 O6 S2
		579.1625	-2.3	-4.0	9.5	64.7	C24 H32 N2 O11 Na S

Figure S35: HRESI-MS Spectrum of compound **6h**

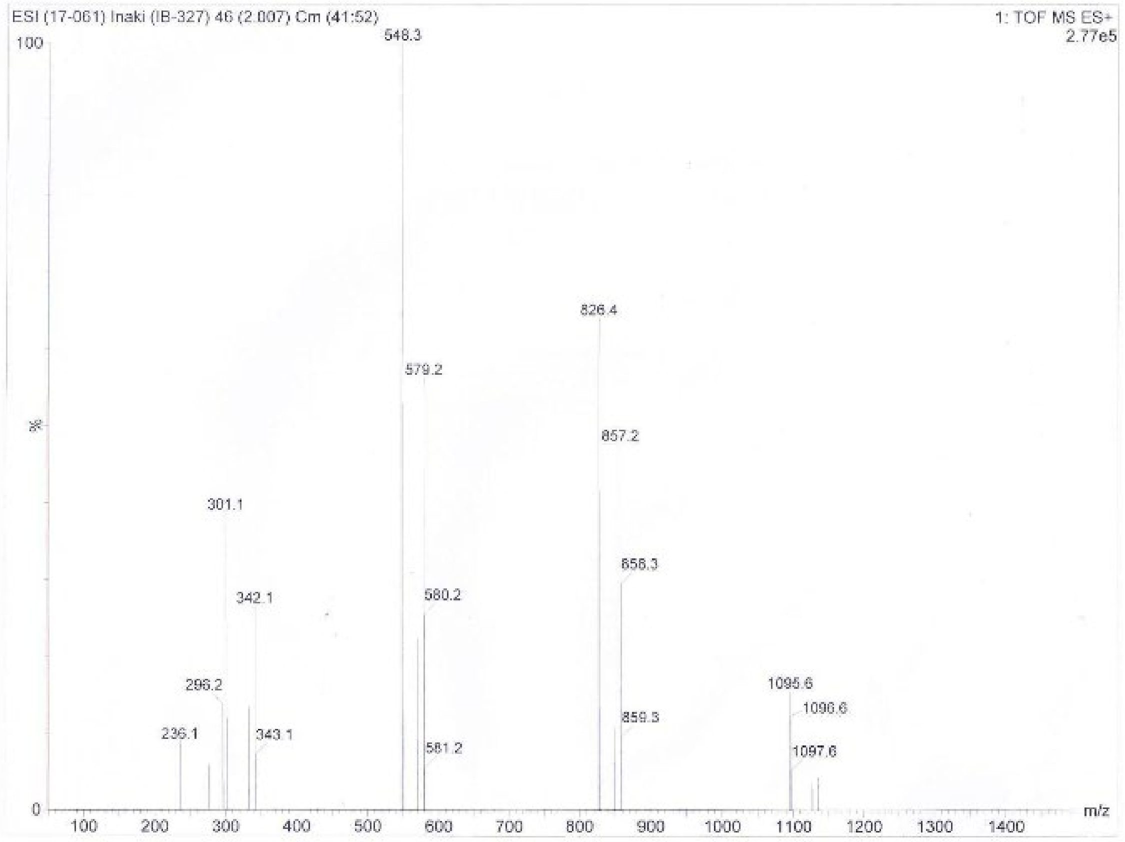

Figure S36: ESI-MS Spectrum of compound **6h**

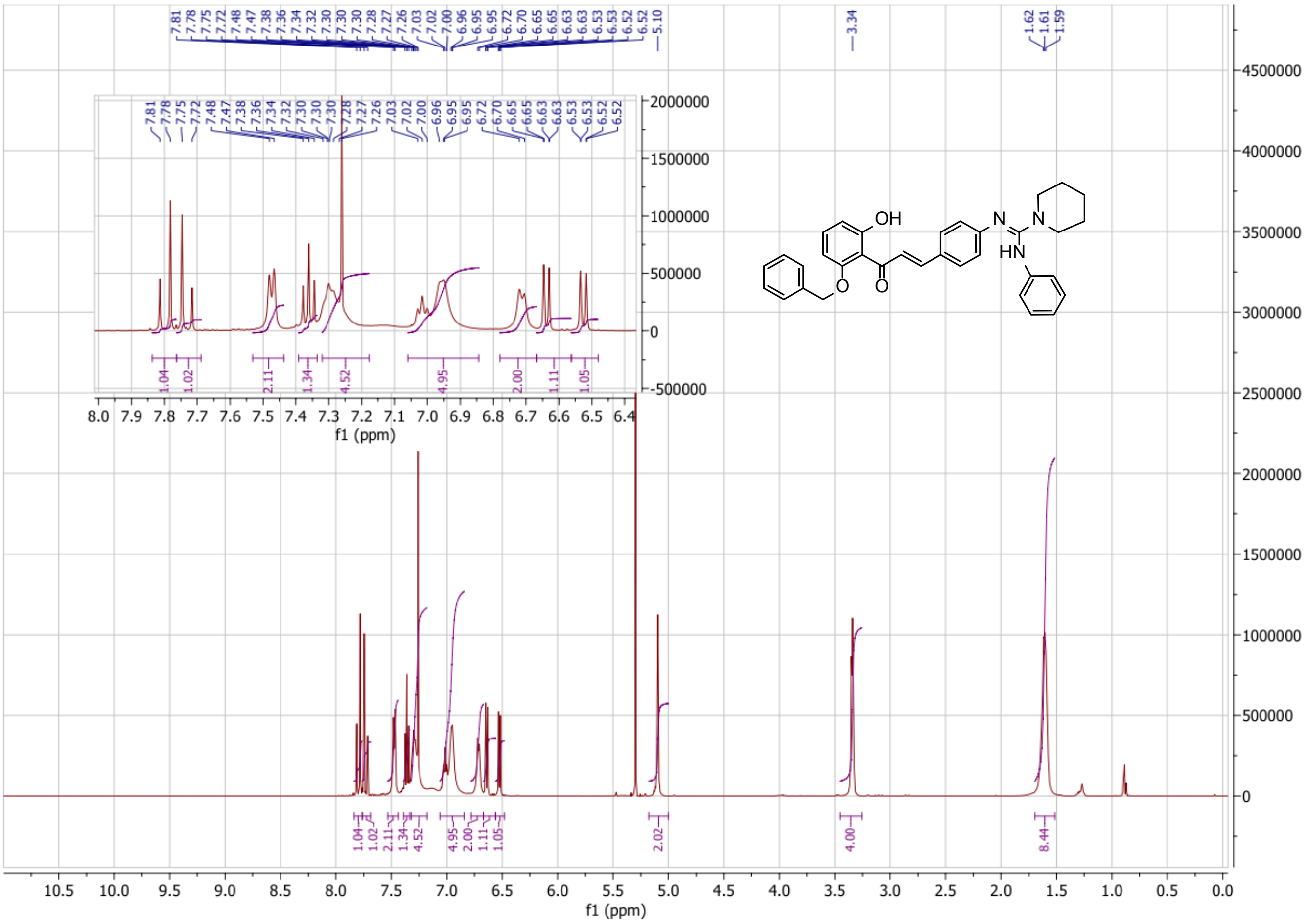

Figure S37: ¹H-NMR (500 MHz, $CDCl_3$) Spectrum of Compound **6i**

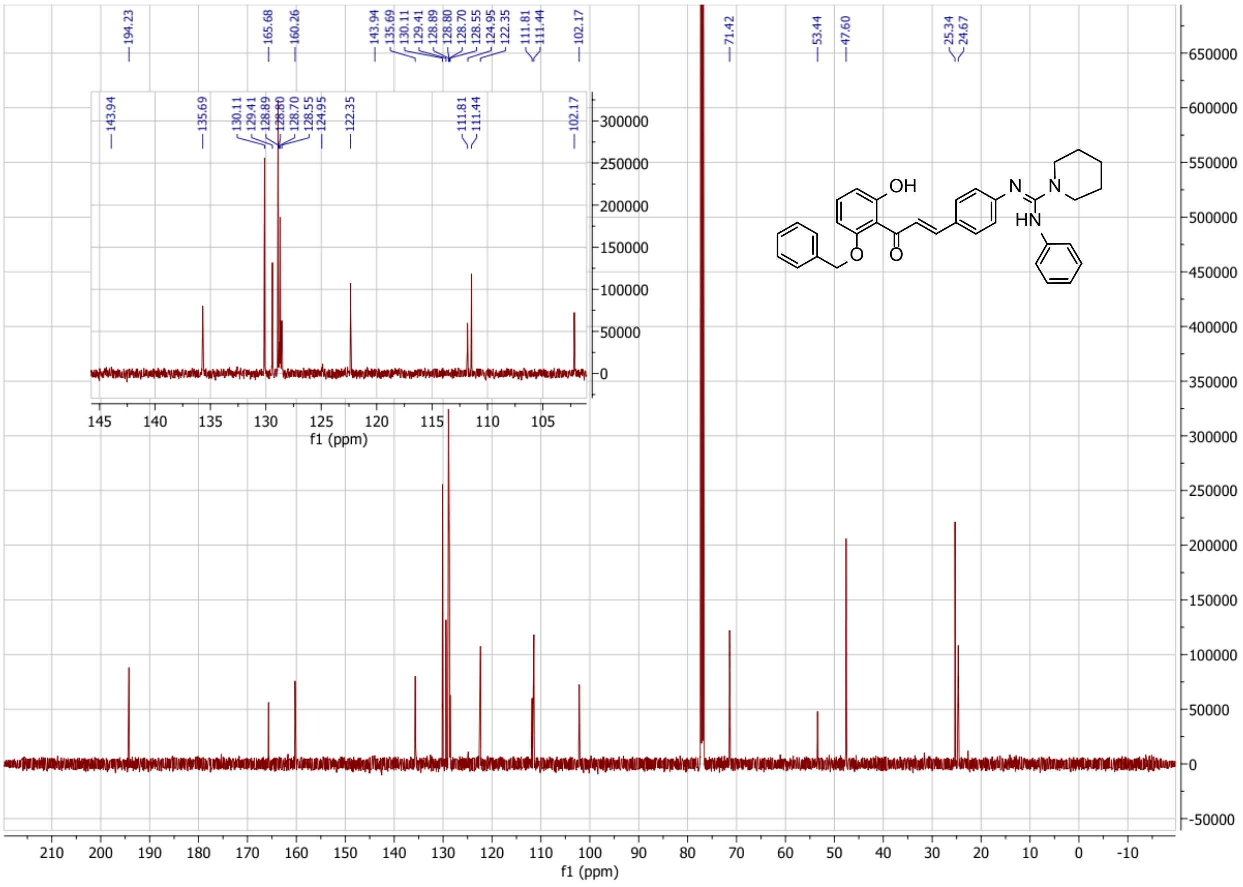

Figure S38: ¹³C-NMR (125 MHz, $CDCl_3$) Spectrum of Compound **6i**

Elemental Composition Report **Page 1**

Tolerance = 5.0 PPM / DBE: min = -10.0, max = 1000.0
Element prediction: Off
Number of isotope peaks used for i-FIT = 2

Monoisotopic Mass, Even Electron Ions
2601 formula(e) evaluated with 6 results within limits (all results (up to 1000) for each mass)
Elements Used:
C: 0-60 H: 0-100 N: 0-4 O: 0-12 Na: 0-1 S: 0-2

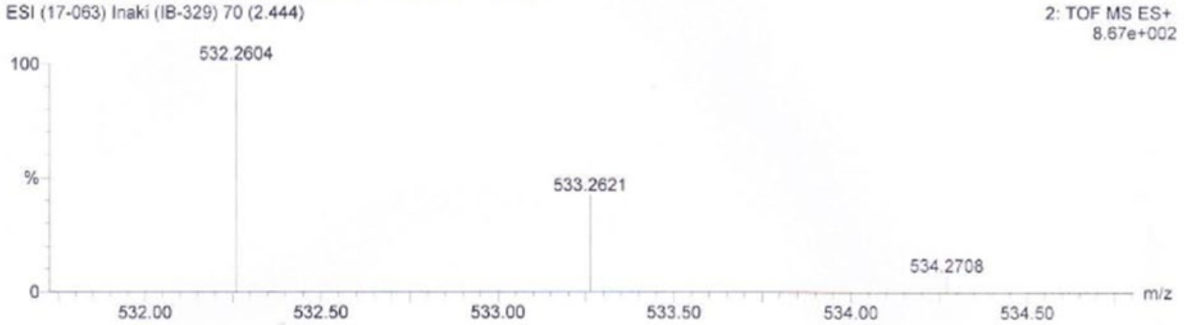

Minimum:	60.00				-10.0		
Maximum:	100.00		100.0	5.0	1000.0		
Mass	RA	Calc. Mass	mDa	PPM	DBE	i-FIT	Formula
532.2604	100.00	532.2600	0.4	0.8	19.5	0.5	C34 H34 N3 O3 *
		532.2610	-0.6	-1.1	11.5	4.2	C29 H39 N3 O3 Na S
		532.2614	-1.0	-1.9	0.5	18.6	C22 H46 N O9 S2
		532.2616	-1.2	-2.3	20.5	0.0	C37 H35 N O Na
		532.2590	1.4	2.6	-2.5	25.2	C20 H47 N O9 Na S2
		532.2580	2.4	4.5	5.5	12.5	C25 H42 N O9 S

Figure S39: HRESI-MS Spectrum of Compound **6i**

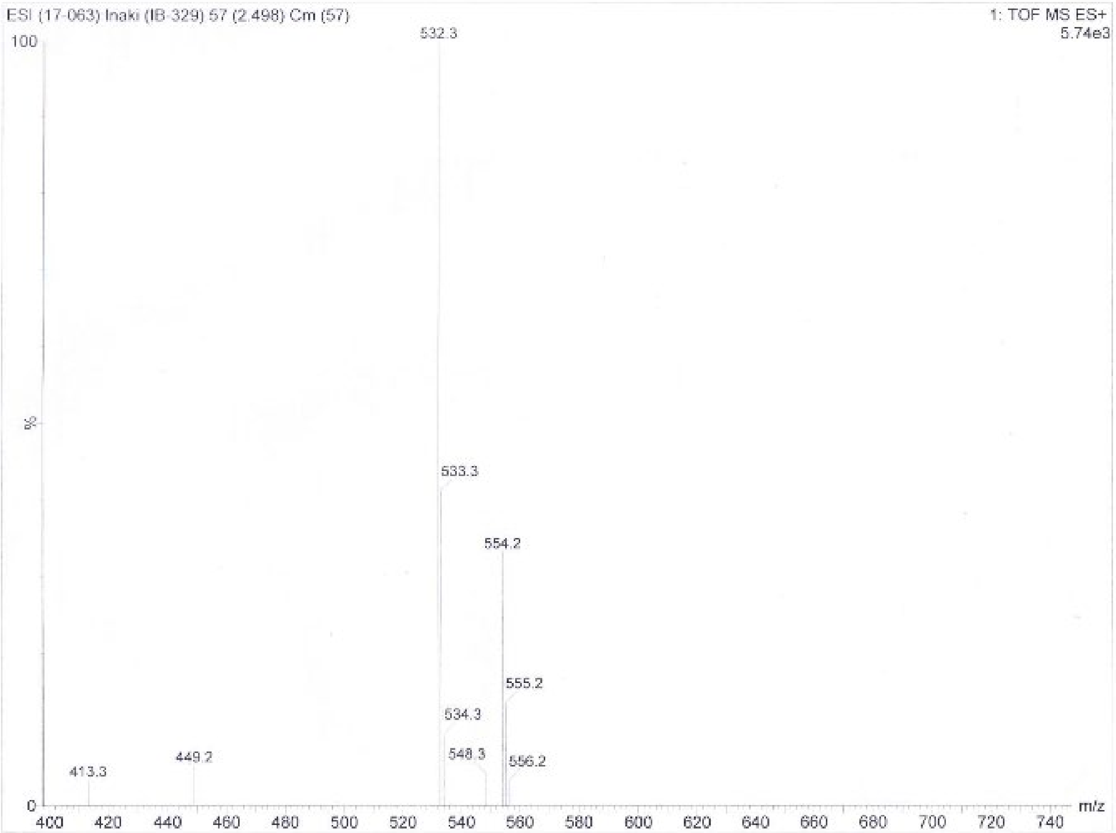

Figure S40: ESI-MS Spectrum of Compound **6i**

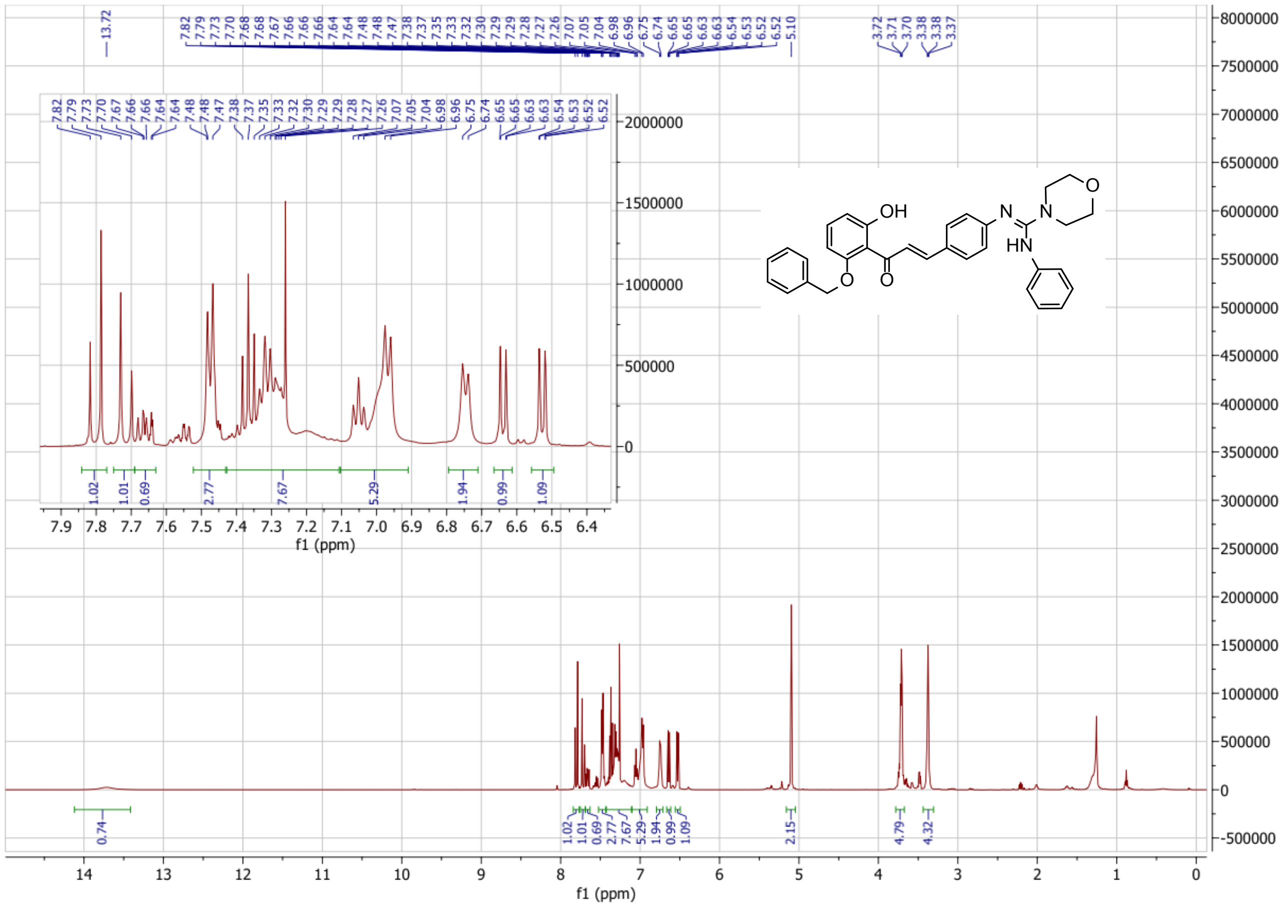

Figure S41: ^{1}H-NMR (500 MHz, $CDCl_3$) Spectrum of Compound **6j**

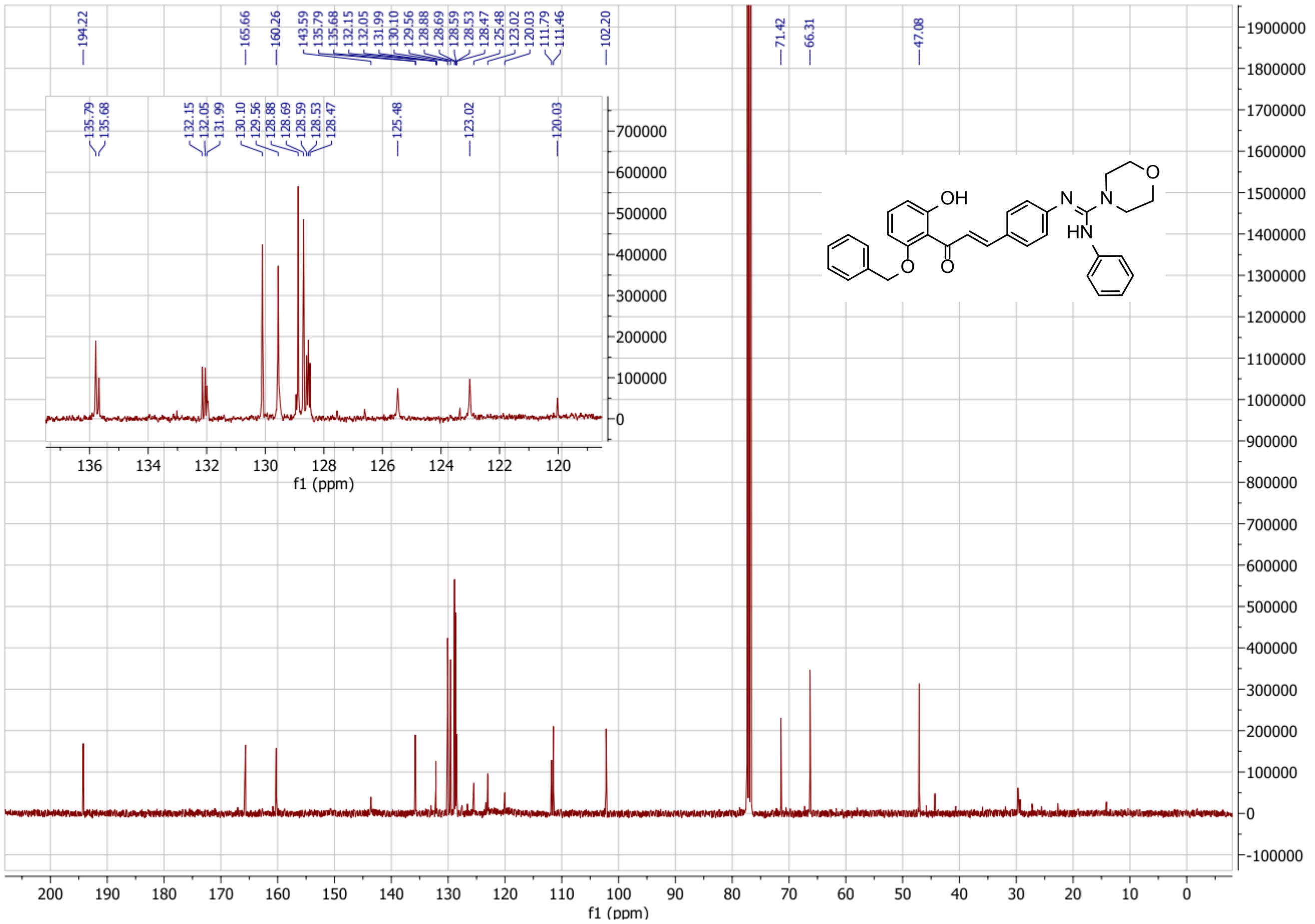

Figure S42: ^{13}C-NMR (125 MHz, $CDCl_3$) Spectrum of Compound **6j**

Elemental Composition Report **Page 1**

Tolerance = 5.0 PPM / DBE: min = -10.0, max = 1000.0
Element prediction: Off
Number of isotope peaks used for i-FIT = 2

Monoisotopic Mass, Even Electron Ions
2611 formula(e) evaluated with 6 results within limits (all results (up to 1000) for each mass)
Elements Used:
C: 0-60 H: 0-100 N: 0-4 O: 0-12 Na: 0-1 S: 0-2

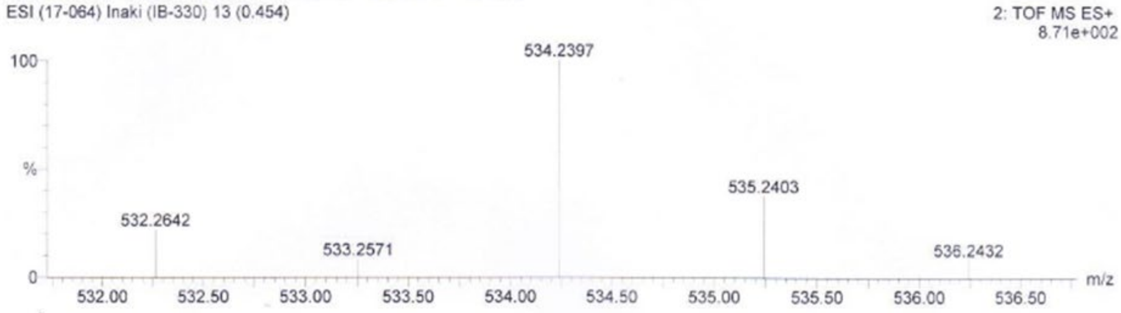

Mass	RA	Calc. Mass	mDa	PPM	DBE	i-FIT	Formula
Minimum:	60.00				-10.0		
Maximum:	100.00		100.0	5.0	1000.0		
534.2397	100.00	534.2393	0.4	0.7	19.5	0.1	C33 H32 N3 O4 *
		534.2402	-0.5	-0.9	11.5	1.2	C28 H37 N3 O4 Na S
		534.2407	-1.0	-1.9	0.5	11.9	C21 H44 N O10 S2
		534.2409	-1.2	-2.2	20.5	1.1	C36 H33 N O2 Na
		534.2383	1.4	2.6	-2.5	17.6	C19 H45 N O10 Na S2
		534.2373	2.4	4.5	5.5	6.9	C24 H40 N O10 S

Figure S43: HRESI-MS Spectrum of Compound **6j**

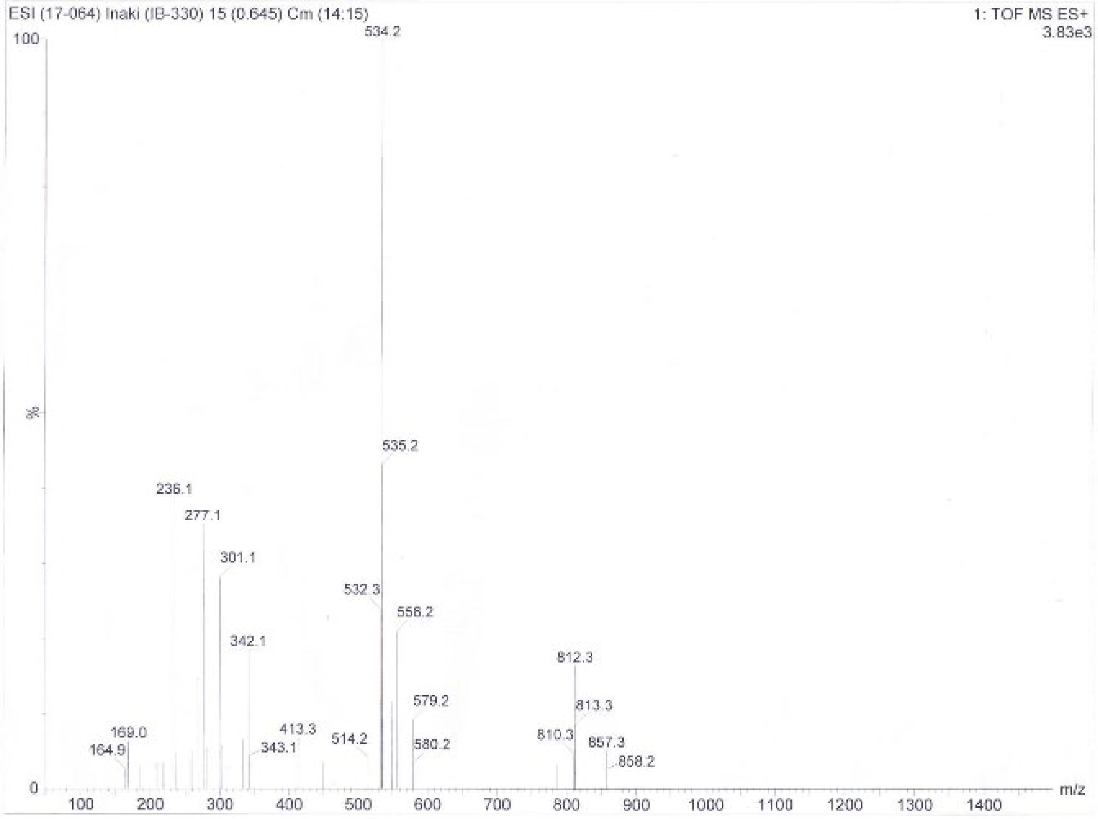

Figure S44: ESI-MS Spectrum of Compound **6j**

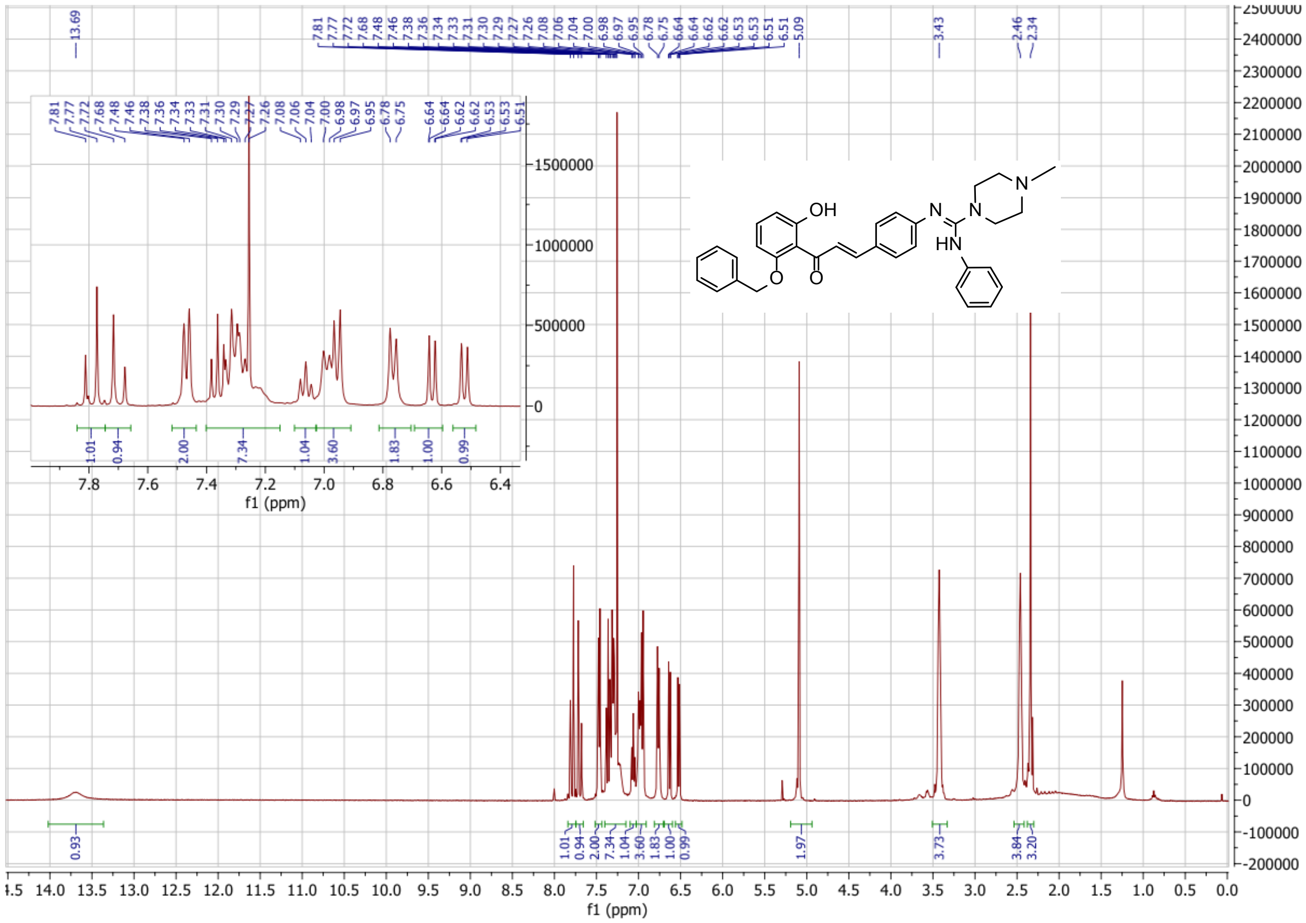

Figure S45: ^{1}H -NMR (500 MHz, $CDCl_3$) Spectrum of compound **6k**

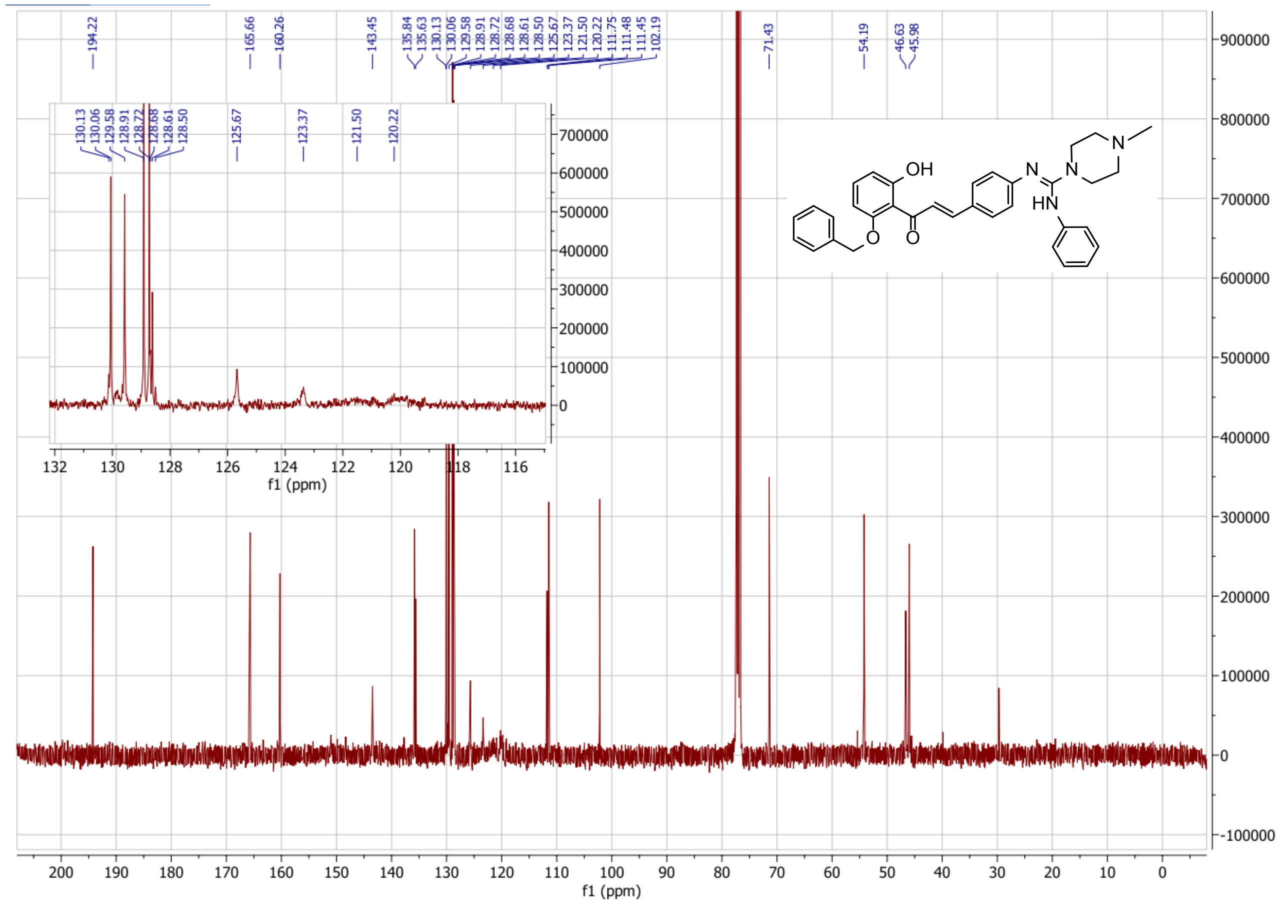

Figure S46: ^{13}C -NMR (125 MHz, $CDCl_3$) Spectrum of compound **6k**

Elemental Composition Report

Page 1

Tolerance = 5.0 PPM / DBE: min = -10.0, max = 1000.0
Element prediction: Off
Number of isotope peaks used for i-FIT = 2

Monoisotopic Mass, Even Electron Ions
2682 formula(e) evaluated with 13 results within limits (all results (up to 1000) for each mass)
Elements Used:
C: 0-60 H: 0-100 N: 0-4 O: 0-12 Na: 0-1 S: 0-2

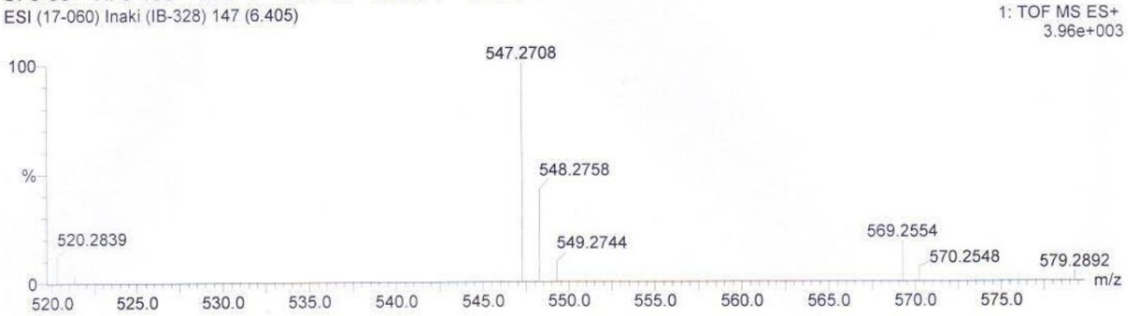

Mass	RA	Calc. Mass	mDa	PPM	DBE	i-FIT	Formula
Minimum:	60.00				-10.0		
Maximum:	100.00		100.0	5.0	1000.0		
547.2708	100.00	547.2709	-0.1	-0.2	19.5	2.0	C34 H35 N4 O3 *
		547.2705	0.3	0.5	6.5	33.5	C28 H44 O7 Na S
		547.2704	0.4	0.7	13.5	1.8	C34 H43 O2 S2
		547.2699	0.9	1.6	-2.5	113.3	C20 H48 N2 O9 Na S2
		547.2719	-1.1	-2.0	11.5	18.3	C29 H40 N4 O3 Na S
		547.2696	1.2	2.2	14.5	8.2	C33 H39 O7
		547.2723	-1.5	-2.7	0.5	83.2	C22 H47 N2 O9 S2
		547.2725	-1.7	-3.1	20.5	0.1	C37 H36 N2 O Na
		547.2689	1.9	3.5	5.5	55.9	C25 H43 N2 O9 S
		547.2730	-2.2	-4.0	2.5	83.6	C24 H44 O12 Na
		547.2730	-2.2	-4.0	9.5	19.3	C30 H43 O7 S
		547.2685	2.3	4.2	16.5	8.0	C32 H36 N4 O3 Na
		547.2683	2.5	4.6	-3.5	154.6	C17 H47 N4 O11 S2

Figure S47: HRESI-MS Spectrum of Compound **6k**

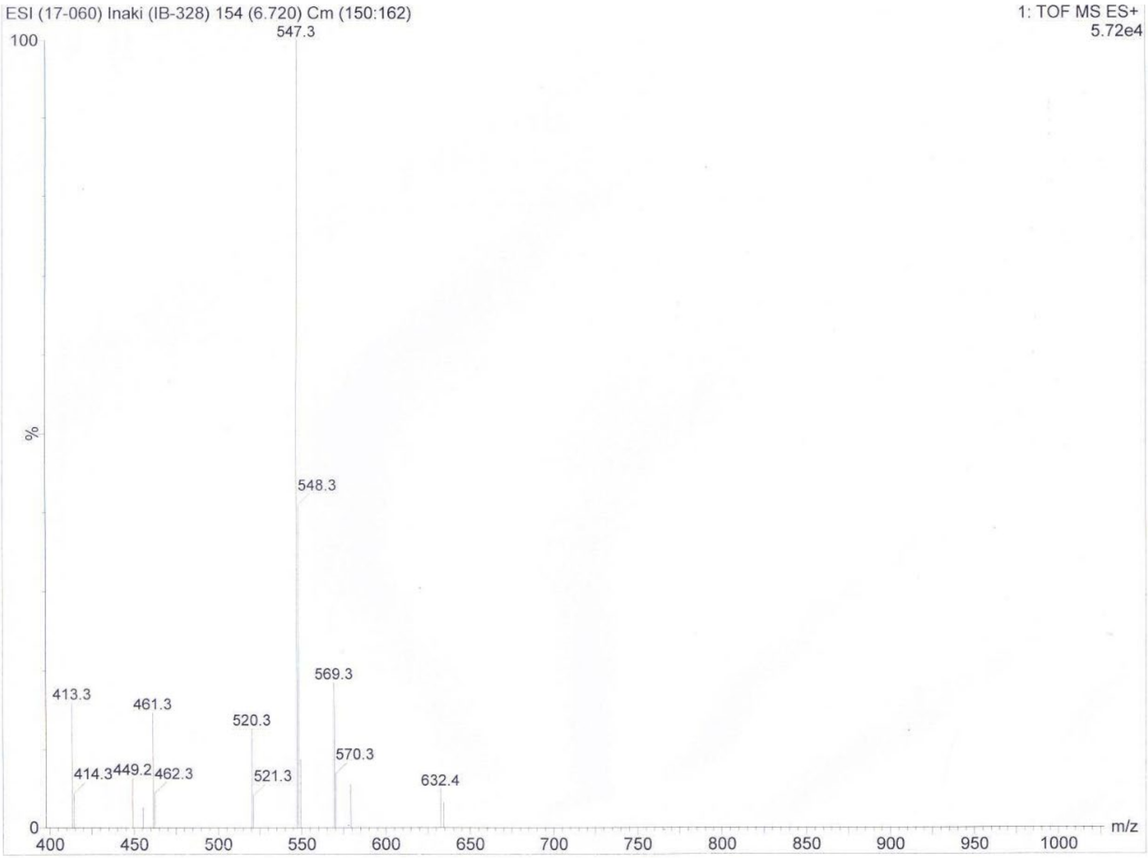

Figure S48: ESI-MS Spectrum of Compound **6k**
